# Tilianin improves cognition in a vascular dementia rodent model by targeting miR-193b-3p/CaM- and miR-152-3p/CaMKIIα-mediated inflammatory and apoptotic pathways

**DOI:** 10.3389/fimmu.2023.1118808

**Published:** 2023-04-19

**Authors:** Ting Sun, Linjie Tan, Mimin Liu, Li Zeng, Kaiyue Zhao, Zhongdi Cai, Shengnan Sun, Zhuorong Li, Rui Liu

**Affiliations:** Institute of Medicinal Biotechnology, Chinese Academy of Medical Sciences and Peking Union Medical College, Beijing, China

**Keywords:** calcium/calmodulin-dependent kinase II, calmodulin, inflammation, microRNA-152-3p, microRNA-193b-3p, tilianin, vascular dementia

## Abstract

**Introduction:**

Although vascular dementia (VaD) is the second most prevalent form of dementia, there is currently a lack of effective treatments. Tilianin, isolated from the traditional drug *Dracocephalum moldavica* L., may protect against ischemic injury by inhibiting oxidative stress and inflammation via the CaMKII-related pathways but with weak affinity with the CaMKII molecule. microRNAs (miRNAs), functioning in post-transcriptional regulation of gene expression, may play a role in the pathological process of VaD via cognitive impairment, neuroinflammatory response, and neuronal dysfunction. This study aimed to investigate the role of tilianin in VaD therapy and the underlying mechanism through which tilianin regulates CaMKII signaling pathways based on miRNA-associated transcriptional action.

**Methods:**

Rats with 2-vessel occlusion (2VO), a standard model of VaD, were treated with tilianin, vehicle control, and target overexpression or downregulation. High-throughput sequencing, qRT-PCR, and western blot analyses were utilized to identify the downstream target genes and signaling pathways of tilianin involved in VaD.

**Results:**

Our results showed that tilianin ameliorated cognitive deficits, neurodegeneration, and microglial and astrocytic activation in rats with 2VO. Subsequent high-throughput sequencing and qRT-PCR analyses revealed that tilianin increased the downregulated miR-193b-3p and miR-152-3p levels in the cortex and hippocampus of 2VO rats. Mechanistically, miR-193b-3p targeting CaM and miR-152-3p targeting CaMKIIα were identified to play a role in VaD-associated pathology, inhibiting the p38 MAPK/NF--κB p65 pathway and decreasing TNF-α and IL-6 levels. Further gain- and loss-of-function experiments for these key genes showed that tilianin-exerted cognitive improvement by activating the p38 MAPK/NF--κB p65 and Bcl-2/Bax/caspase-3/PARP pathways in the brain of 2VO rats was abolished by miR-193b-3p and miR-152-3p inhibition. Moreover, CaM and CaMKIIα overexpression eliminated the elevated effects of miR-193b-3p and miR-152-3p on tilianin’s protection against ischemic injury through increased inflammatory reactions and apoptotic signaling.

**Discussion:**

Together, these findings indicate that tilianin improves cognition by regulating the miR-193b-3p/CaM- and miR-152-3p/CaMKIIα-mediated inflammatory and apoptotic pathways, suggesting a potential small-molecule regulator of miRNA associated with inflammatory signaling for VaD treatment.

## 1 Introduction

Vascular dementia (VaD) is an age-related, heterogeneous brain disorder characterized by cognitive impairment attributed to cerebrovascular pathologies (1). VaD is responsible for 15–20% of dementia cases, following only Alzheimer’s disease (AD) as a cause of dementia (2). With the global population aging, the risk of VaD roughly doubles every 5.3 years, developing in around 15–30% of subjects three months after a stroke (3). As the underlying etiology of VaD is unclear, management is limited and there are currently no officially approved treatments. Drugs currently used for VaD, such as AChE inhibitors and antioxidants, only provide symptomatic treatment and cannot prevent progression (4). Therefore, continued research efforts are needed to investigate the pathogenesis of VaD and develop novel and effective drugs for treatment.

VaD is triggered and exacerbated by vascular lesions resulting from chronic cerebral hypoperfusion, manifesting as multiple pathophysiological changes, including oxidative stress, central cholinergic system dysfunction, neuroinflammation, and neuronal apoptosis (4–8). Excitotoxicity of hypoperfusion is considered to be one of the important factors in VaD pathology.

Calmodulin (CaM) is a ubiquitous calcium sensor protein involved in almost all intracellular events. Many neuropathological injuries associated with VaD are induced by calcium/calmodulin (Ca^2+^/CaM)‐dependent enzymes (9), among which Ca^2+^/CaM‐dependent protein kinase II (CaMKII) is a major mediator of abnormal excitotoxicity underlying hypoperfusion-induced cognitive deficits (10). Among the four subtypes (α, β, γ, and δ), CaMKIIα is expressed almost exclusively in the brain and comprises more than 1.3% of total proteins in the regions associated with learning and memory, including the cortex and hippocampus (11). CaMKII is highly sensitive to ischemic insults. When Ca^2+^/CaM acts *via* CaMKII, CaMKII acts like a molecular switch to exert various effects implicated in the regulation of neuronal survival and cell death (12, 13). In such cases, post-synaptic injury can lead to impaired learning and memory as well as extra-synaptic hyperphosphorylation cascades resulting in oxidative stress, mitochondrial damage, apoptosis, and inflammation (14, 15). Therefore, CaMKII is a potential drug target for post-insult brain protection (16).

*Dracocephalum moldavica* L. (*D. moldavica*) is used as a traditional medicine in China to treat coronary disease, hypertension, headache, colds, asthma, and dermatitis, among other conditions (17). We previously reported that tilianin extracted from the total flavonoids of *D. moldavica* (TFDM) has neuroprotective effects against cognitive deficits (9, 18, 19). Tilianin has extensive pharmacological properties, including cardioprotective, anti-atherogenic, and anti-asthmatic effects (20–23). The molecular mechanisms of tilianin involve many key components of cellular signaling transduction pathways associated with oxidative stress-mediated inflammation, apoptosis, and angiogenesis (20, 23). We found that tilianin exerted neuroprotective and cardioprotective effects by inhibiting CaMKII-dependent apoptosis and inflammatory pathways (18, 24). We also demonstrated that tilianin ameliorated neuronal and cardiomyocytic damage against multiple ischemic injuries by inhibiting phosphorylated (p-) and oxidative (ox-) CaMKII expression, rather than by directly binding to specific active sites of CaMKII protein molecules (9). However, how tilianin inhibits CaMKII expression for anti-ischemic effects has not been clarified.

Prior research has shown that CaM and CaMKII mRNA expression levels are consistent (25). *CAM* is transcribed in a gene-specific manner in rodent brains (26) and involved in the Ca^2+^-mediated regulation of gene expression, playing a role in the Ca^2+^/CaM/CaMKII signaling pathway in response to ischemic stimuli. In turn, CaMKII participates in mRNA delivery and translation of CaM (27). These observations indicate that post-transcriptional control contributes to the regulation of CaM/CaMKII cascade transduction. Therefore, the effects and the underlying mechanism of tlianin based on transcriptional regulation of the CaM/CaMKII signaling pathway warrants further research.

MicroRNAs (miRNAs) are post-transcriptional regulators of gene expression (5). Some miRNAs, such as miR-15a/16-1 (28), miR-93 (29), miR-3184-3p, miR-6875-5p (30), and miR-126 (31), play a role in the pathological process of VaD since they have been involved in cognitive impairment, vascular dysfunction, neuroinflammatory response, blood-brain barrier destruction, and synaptic loss. There is also evidence that miRNAs play a regulatory role in aberrant CaM/CaMKII cascades in ischemia and reperfusion. For example, miR-181a has been shown to participate in the CaM/CaMKII pathway during the action of the Fragile X mental retardation protein in controlling the proliferation and angiogenesis of human umbilical vein endothelial cells (32). CaM and CaMKII expression is affected at the post-transcriptional level by miR-26b (33), miR-145 (34), and miR-148a (35), thereby contributing to the progression of ischemic injury. However, the spatiotemporal mechanisms of these specific miRNAs in regulating the CaM/CaMKII pathway in the context of VaD are not fully understood.

In the present study, we investigated the protective effects of tilianin in experimental VaD using 2-vessel occlusion (2VO) rat model and an *in vitro* oxygen-glucose deprivation (OGD) cell model. Furthermore, we used high-throughput sequencing analyses, qRT-PCR, western blot, dual-luciferase reporter assay, and gene gain- and loss-of-function approaches to explore the function and molecular mechanisms of tilianin for regulating CaM/CaMKIIα pathways involved in VaD. We observed that two miRNAs, namely miR-193b-3p and miR-152-3p, played a prominent protective role in the cognitive improvement of tilianin against VaD, which inhibited the CaM/CaMKIIα mediated inflammatory and apoptotic pathways.

## 2 Material and methods

### 2.1 Drugs

**Figure 1:**
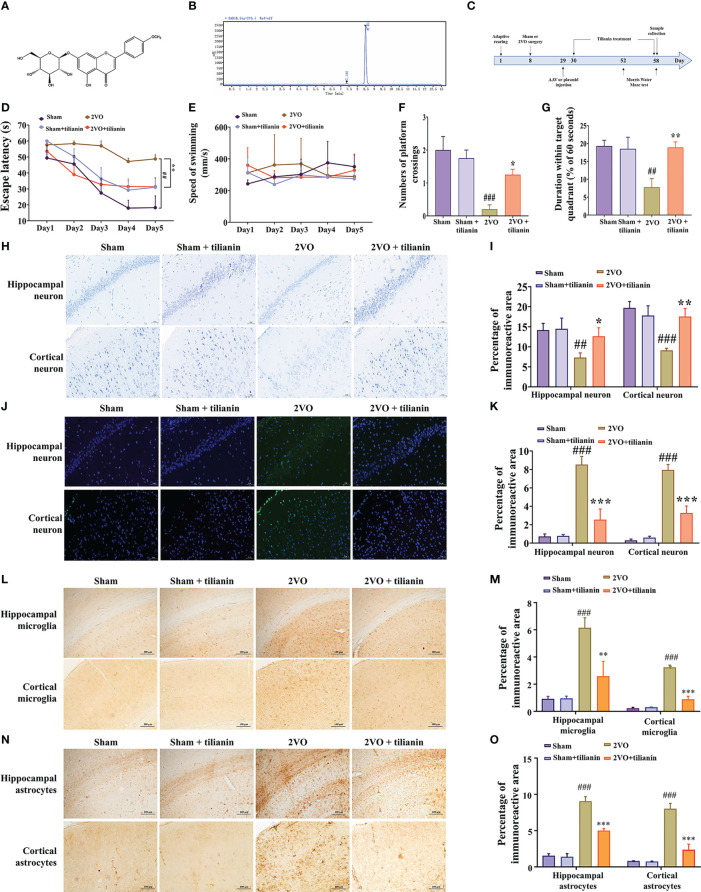
Effects of tilianin on cognitive decline, neurodegeneration, and neuroinflammation in 2VO rats. **(A)** Chemical structure of tilianin. **(B)** HPLC chromatogram of tilianin. The purity of tilianin is 99.02%. The retention time of tilianin peak is 8.464 min. **(C)** Overview of the experimental design, including the surgical procedures, tilianin treatment, and gene modification administration. **(D)** Escape latency of 2VO rats in the MWM navigation test (*n* = 8). **(E)** Swimming speed of rats in the navigation test (*n* = 8). **(F)** Number of platform crossings by 2VO rats (*n* = 8). **(G)** Duration within the target quadrant for 2VO rats (*n* = 8). **(H)** Representative images of Nissl-stained cortical and hippocampal neurons (*n* = 3). Scale bar = 50 μm. **(I)** Quantitative analysis of immunoreactivity of Nissl staining in the cerebral cortex and hippocampus of 2VO rats (*n* = 3). **(J)** Representative images of TUNEL-stained cortical and hippocampal neurons (*n* = 3). Scale bar = 50 μm. **(K)** Quantitative analysis of immunoreactivity of TUNEL staining in the cerebral cortex and hippocampus of 2VO rats (*n* = 3). **(L)** Representative images of IBA1-stained cortical and hippocampal microglia (*n* = 3). Scale bar = 100 μm. **(M)** Quantitative analysis of immunoreactivity of IBA1 staining in the cerebral cortex and hippocampus of 2VO rats (*n* = 3). **(N)** Representative images of GFAP-stained cortical and hippocampal astrocytes (*n* = 3). Scale bar = 100 μm. **(O)** Quantitative analysis of immunoreactivity of GFAP staining in the cerebral cortex and hippocampus of 2VO rats (*n* = 3). The results indicate the mean ± SD. ^##^ *P*<0.01, ^###^ *P*<0.001 *vs.* sham, ^*^ *P*<0.05, ^**^ *P*<0.01, ^***^ *P*<0.001 *vs.* 2VO.

Tilianin with 99.02% purity, shown by high-performance liquid chromatography (HPLC) was isolated from *D. moldavica* (30, 36). Briefly, the above-ground parts of *D. moldavica* were ground to powder and refluxed three times with 40% ethanol at 100 °C. The combined ethanol solution was filtered and evaporated at low pressure to obtain the crude extract, which was then separated by column chromatography using HPD600 resin and eluted with water, 50%, and 70% ethanol. Impurities were removed using a silica gel column and the pure compound was collected. The structure and HPLC image of tilianin were shown in **Figures 1A, B**.

### 2.2 Animals

Male Sprague–Dawley rats aged seven weeks and weighing 220 ± 20 g were purchased from Vital River Laboratory Animal Technology Co., Ltd. (Beijing, China). A total of 136 rats were included in this study. All experiments were approved by the Experimental Animal Care and Use Committee of the Institute of Medicinal Biotechnology (No. IMB-202102-D7).

### 2.3 Animal model and treatment

The two-vessel occlusion (2VO) model is an established model of VaD. Following the standard procedure, rats were anesthetized with pentobarbital sodium (30 mg/kg). We then separated the bilateral common carotid arteries and ligated them with a No.4–0 surgical suture. Rats in the sham group underwent the same surgery but without ligating the bilateral common carotid arteries. During the operation, the anal temperature of the rats was maintained at 37 °C. The rats were tested 21 days post-surgery.

Next, the rats were randomly divided into three first-level groups: tilianin treatment group, target overexpression or downregulation group, and target overexpression or downregulation with tilianin treatment group. Within the tilianin treatment group, rats were subgrouped into sham (*n* = 8), sham+40 mg/kg tilianin (*n* = 8), 2VO (*n* = 8), and 2VO+40 mg/kg tilianin (*n* = 8) groups. In the target overexpression or downregulation group, rats were subgrouped as sham+negative control mimics (NCM) (*n* = 8), sham+miR-152-3p mimics (152M) (*n* = 8), sham+miR-193b-3p mimics (193M) (*n* = 8), 2VO+NCM (*n* = 8), 2VO+152M (*n* = 8), 2VO+193M (*n* = 8), 2VO+152M+CaMKIIα (*n* = 8), and 2VO+193M+CaM (*n* = 8) groups. In the target overexpression or downregulation with tilianin treatment group, rats were subgrouped into sham+negative control inhibitor (NCI) (*n* = 8), 2VO+NCI (*n* = 8), 2VO+NCI+tilianin (*n* = 8), 2VO+miR-152-3p inhibitor (152I)+tilianin (*n* = 8), and 2VO+miR-193b-3p inhibitor (193I)+ tilianin (*n* = 8) groups. Rats in each group received the corresponding operations and drugs. The experimental design is shown in **Figure 1C**.

Group I (negative control group) received a daily corn oil dose (5 mL/kg/body weight, BW) through a gastric tube for one month.

Rats in the tilianin treatment group received a daily dose of 40 mg/kg tilianin that was dissolved in 0.5% carboxymethylcellulose sodium (CMC-Na) (5 mL/kg/body weight) with a gastric tube for four weeks. Rats in the sham and 2VO groups were given 0.5% CMC-Na vehicle (5 mL/kg/body weight) over the same period. For the stereotactic injection procedures, rats were anesthetized with isoflurane, placed in an animal stereotaxic apparatus (RWB Life Science Co, Ltd., China), and the bregma was marked. The injection coordinates were as follows: anteroposterior, -1.0 mm; mediolateral, ± 1.5 mm; and dorsoventral, -4.5 mm. A total of 8 μL adeno-associated viruses (AAVs) were injected into the lateral ventricle. After the injection, the needle was left in place for 10 min to allow the liquid to be fully absorbed. The miR-193b-3p mimics (AAV-CAG-miR-193b-3p), miR-152-3p mimics (AAV-CAG-miR-152-3p), miR-193b-3p inhibitor (AAV-CAG-miR-193b-3p inhibitor), miR-152-3p inhibitor (AAV-CAG-miR-152-3p inhibitor), CAML (AAV-CAG-CALM), and CAMK2A (AAV-CAG-CAMK2A) were synthesized by Sangon Biotech (Shanghai, China); titer, ≥ 1×VG^12^ vg/mL. The transfection efficiency of AAV-miRNAs is shown in **Figure S1**.

### 2.4 Cell culture and treatment

Human neuroblastoma SH-SY5Y cells and human embryonic kidney cells (HEK293) (ATCC, USA) were cultured in high-glucose Dulbecco’s Modified Eagle Medium (DMEM) with 10% fetal bovine serum (Gibco, USA) and penicillin at 37°C in an atmosphere of 5% CO_2_. The OGD injury model was used to simulate the ischemic injury associated with VaD *in vitro* (9). SH-SY5Y cells were subjected to OGD, similarly as previously described (37). SH-SY5Y and HEK293 cells were transfected using Lipofectamine 2000 according to the manufacturer’s protocols (Invitrogen, Carlsbad, CA, USA). SH-SY5Y cells in individual wells were transfected with 152M, 193M, NCM, NCI, pCMV6-CaMKIIα, and pCMV6-CaM (Sangon Biotech, Shanghai, China). HEK293 cells were co-transfected with recombinant PGL3 plasmids and miRNA sequences to validate the miRNA targets.

### 2.5 Morris water maze (MWM) test

The MWM is a classic experiment used to evaluate spatial cognition and memory in rats. The test consisted of positioning navigation for the first 5 days and a probe trial on day 6. For positioning navigation, the rats were placed in the water in the four quadrants of the maze for four tests. The time taken for the rats to successfully find the hidden platform (escape latency) and swimming speed were recorded. The average time and average swimming speed of the four tests were used for the analysis. If the rats did not find the platform within 60 s, they were directed to the platform and allowed to stay there for 10 s to learn its location. In the probe trial, the rats entered the water from the quadrant furthest from the original platform. The time spent within the target quadrant (position of the original platform) and number of times crossing the original platform were recorded.

### 2.6 Histological experiments

Rats were sacrificed using pentobarbital sodium and perfused with 4% paraformaldehyde. The brains were dehydrated with graded concentrations of ethanol (50%, 70%, 85%, 95%, 100%, and 100%), and then transparentized with xylene and ethanol (50% xylene + 50% ethanol, 100% xylene, 100% xylene). Next, the brains were placed in paraffin-xylene solutions (50% paraffin, 100% paraffin) at 60 °C, followed by embedding in paraffin. The embedding blocks were fixed on the specimen table, cut into 4 μm sections, and subsequently placed in warm water at 45 °C. After the sections were unfolded, they were attached to the slides. Subsequently, the sections of the cortex and hippocampus were processed for Nissl staining (Servicebio, Wuhan, China) and TUNEL staining (Norvan, Nanjing, China) according to the manufacturers’ instructions.

### 2.7 Immunohistochemistry

After blocking with 5% bovine serum albumin (BSA) for 1 h, the cortex and hippocampus sections were immunohistochemically stained for GFAP and IBA1 with primary antibodies (**Table S1**) overnight at 4°C, followed by goat anti-rabbit IgG (H+L)-HRP secondary antibody (**Table S1**) for 1 h at room temperature. Both primary and secondary antibodies were diluted in phosphate buffer saline containing 0.3% Triton X-100 (PBST) and 1% BSA. Histological images were collected under a bright microscope (Olympus CX33-LV2000, Tokyo, Japan) using a 20× objective with the same condition at a resolution of 1280 × 960 pixels. The percentage area of the GFAP or IBA1 positive cells in the hippocampus and cortex in each sections were calculated by ImageJ software (NIH, MD, USA). The mean percentage area were calculated from 3 random microscopic fields from each section for each animal by one person unaware of treatment history. A total of 3 animals per group were analyzed.

### 2.8 High-throughput sequencing and bioinformatics analyses

Brain tissues were screened for abnormal miRNAs and mRNAs by high-throughput sequencing analysis (Sangon Biotech, Shanghai, China). Differentially expressed miRNAs and mRNAs in the brain of rats in the sham, 2VO, and tilianin groups were screened using the criteria of *P*<0.05 and |log2Fold Change|>1. The miRDB (http://www.mirdb.org/), miRWalk (http://mirwalk.umm.uni-heidelberg.de/), and TargetScan (https://www.targetscan.org/vert_80/) databases were used to predict the target genes of the miRNAs. Gene Ontology (GO) and Kyoto Encyclopedia of Genes and Genomes (KEGG) enrichment were used to explore the molecular mechanisms and signaling pathways of VaD pathogenesis. A protein-protein interaction (PPI) network was constructed using STRING 11.0 software to visualize the PPI network of differentially expressed genes.

### 2.9 RNA isolation and qRT-PCR analysis

Total RNA in the hippocampus and cortex of rats, SH-SY5Y cells, HEK293 cells, or plasma was extracted with TRIzol reagent from Invitrogen. For mRNA, cDNA was synthesized by HiscriptIII RT SuperMix and the qRT-PCR was completed using SYBR Master Mix kits (Vazyme, Nanjing, China). For miRNA and U6, miRNA Synthesis Kit and miRNA SYBR Master Mix were used (Vazyme, Nanjing, China). Sangon Biotech (Shanghai, China) produced all primers (**Table S2**). Each experiment contained three technical replicates and four biological replicates.

### 2.10 Cell viability assay

SH-SY5Y cells were cultured in 96-well plates at a density of 1×10^4^ cells/well and transfected as described above. After 24 h, the cells were cultured with 100 μL Cell Counting Lite (CCL) solution (CellCounting-Lite 2.0 Luminescent Cell Viability Assay, Novazan Co.LTD, Nanjing) for 15 min. A Spark 20M multimode micrometer (Tecan Group Ltd., Mannedorf, Switzerland) was used to determine the optical density (OD). Each assay contained three technical replicates and three biological replicates.

### 2.11 Apoptosis assay

The degree of apoptosis in SH-SY5Y cells was determined using the Annexin V (Annexin V-PE)/7-amino-actinomycin D (7-AAD) Apoptosis Detection Kit (Vazyme, Nanjing, China). Flow cytometry was used to determine the percentages of apoptotic cells. Each assay contained three technical replicates and three biological replicates.

### 2.12 Western blot

Total protein was extracted from the hippocampus and cortex of rats, SH-SY5Y cells, or HEK293 cells using RIPA Buffer (CWBio, Beijing, China) and then quantified using the bicinchoninic acid (BCA) Protein Assay Kit (Thermo Fisher, USA). The total protein was separated by sodium dodecyl sulfate-polyacrylamide gel electrophoresis (SDS-PAGE) and transferred to nitrocellulose membranes (Millipore, Billerica, MS, USA). After being blocked with 5% skimmed milk for 1 h at 25 °C, the membranes were incubated with primary antibodies (**Table S1**) at 4°C for 8 h. The membranes were then washed three times by Tris-Buffered Saline with Tween-20 (TBST) buffer and incubated with goat anti- (rabbit or mouse) secondary antibody (**Table S1**) for 1 h at 25°C. A Fusion-FX6 imaging system (Vilber Lourmat, Marne-la-Vallée, France) was used for chemiluminescence color rendering and band analysis. Each analysis contained three biological replicates.

### 2.13 Enzyme-linked immunosorbent assay (ELISA)

The tumor necrosis factor-alpha (TNF-α), interleukin-6 (IL-6), and caspase-3 levels in tissues and cells were measured using the corresponding ELISA kits (ImmunoWay Biotechnology Company, Plano, TX, USA) following the manufacturers’ instructions. Each analysis contained three technical replicates and three biological replicates.

### 2.14 Dual-luciferase reporter assay

The miR-152b-3p specific binding sites in the 3’-untranslated regions (3’-UTR) of CAMKIIA and the miR-193-3p binding sites in the 3’-UTR region of CALM were predicted using TargetScanHuman. About 150 bp before and after the binding site of CALM 3’-UTR and miR-193b-3p, CAMKIIA 3’-UTR and miR-152-3p were copied into a GP-miRGLO vector to construct luciferase wild-type (WT) and mutant (MUT) plasmids, in which the MUT plasmid only mutates the binding site. The wild-type (pGL-3’-UTR-CAMKIIA-WT and pGL-3’-UTR-CALM -WT) and mutant (pGL-3’-UTR-CAMKIIA-MUT and pGL-3’-UTR-CALM-MUT) plasmids were then transfected into HEK293 cells using the transfection methods described above. The miR-152-3p mimics, miR-193b-3p mimics, and NC were simultaneously co-transfected. The Dual-Luciferase Reporter Assay Kit (Vanzyme, Nanjing, China) was used 36 h post-transfection and the *Renilla* luciferase report system was used as a control. Each analysis contained three technical replicates and four biological replicates.

### 2.15 Statistical analysis

Statistical analyses were performed using SPSS software (Version 18.0, SPSS, Inc., Chicago, USA). One-way repeated measures analysis of variance (ANOVA) was used to compare the escape latency and swimming speed in the MWM test between groups. One-way ANOVA with Tukey’s *post hoc* test or Student’s unpaired *t*-test was used to analyze other data. Data are reported as the mean ± standard deviation (S.D.). Differences with a *P*-value < 0.05 were considered statistically significant.

## 3 Results

### 3.1 Tilianin treatment attenuates spatial cognitive decline, neurodegeneration, and neuroinflammation in VaD model rats

The 2VO rats exhibited impaired learning compared with sham rats, as manifested by longer escape latency in the orientation navigation test (**Figure 1D**, *P* < 0.01 *vs.* sham). Treatment with 40 mg/kg tilianin significantly reduced the escape latency of 2VO rats (**Figure 1D**, *P* < 0.01 *vs.*2VO). However, there was no significant difference in swimming speed among the groups, indicating no impairment in motor ability (**Figure 1E**). In the probe trial, 2VO rats showed fewer crossings of the position where the previous platform was located and a shorter time spent in the quadrant without the previous platform (**Figures 1F, G**, *P* < 0.01, *P* < 0.001 *vs.* sham). Tilianin treatment significantly improved memory, as demonstrated by an increased number of platform crossings and longer duration within the target quadrant compared with 2VO rats (**Figures 1F, G**, *P*<0.05, *P*<0.01, respectively, *vs.* 2VO). It should be noted that 40 mg/kg tilianin did not significantly affect learning and memory in sham rats.

Nissl and TUNEL staining were used to evaluate histopathological changes in the brain. The number of Nissl bodies was greatly reduced in the hippocampus and cortex of 2VO rats (**Figures 1H, I**, *P* < 0.01, *P* < 0.001 *vs.* sham). However, tilianin increased the number of Nissl bodies in these regions compared with 2VO rats (**Figures 1H, I**, *P* < 0.05, *P* < 0.01 *vs.* 2VO). TUNEL staining showed that there was a significant increase in apoptosis in the 2VO model group (**Figures 1J, K**, both *P* < 0.001 *vs.* sham) and a significant reduction of apoptosis in response to tilianin treatment (**Figures 1J, K**, both *P* < 0.001 *vs.* 2VO).

Activation of glial cells is one of the major features of neuroinflammation, of which activation of microglia and astrocytes are typical symbols. Immunohistochemistry of IBA-1 and GFAP staining showed an increased microglial and astrocytic reaction in the 2VO model group (**Figures 1L–O**, *P* < 0.001 *vs.* sham). Similarly, the activation of microglia and astrocytes was attenuated in response to tilianin treatment (**Figures 1L–O**, *P* < 0.01, *P* < 0.001 *vs.* 2VO). Together, these results showed that tilianin effectively alleviated cognitive deficits, neuronal degeneration, and glial neuroinflammation in 2VO rats.

### 3.2 Identification of miRNAs and mRNAs linked to tilianin treatment in VaD model rats

**Figure 2:**
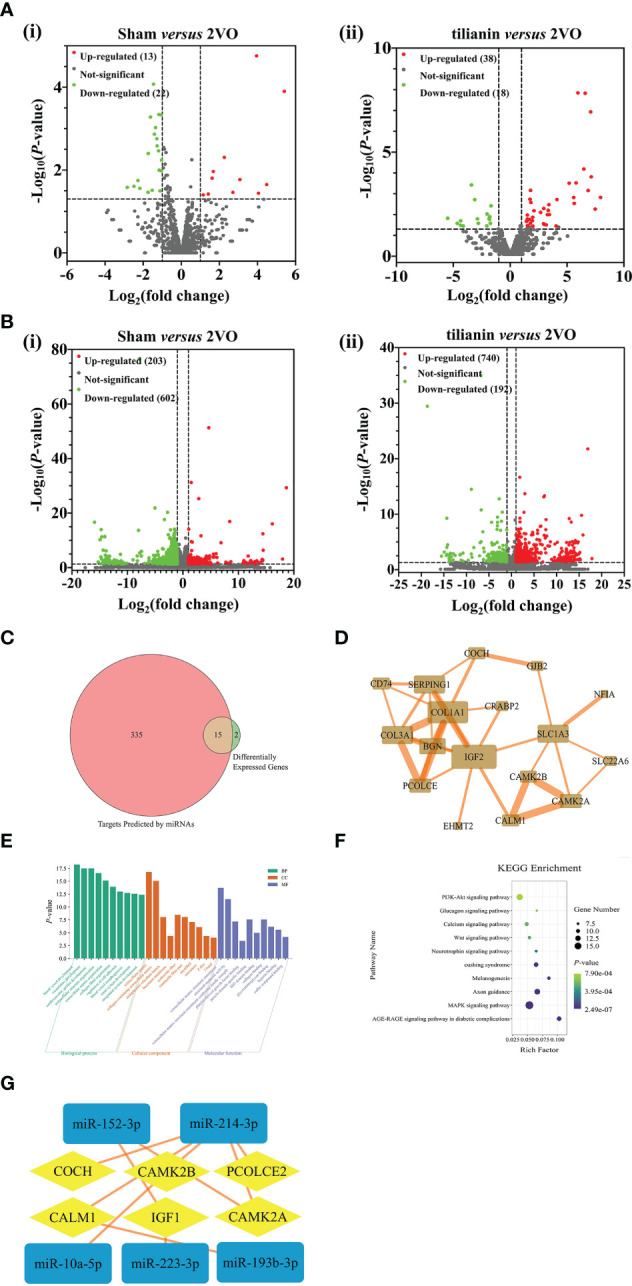
Identification of miR-193b-3p, miR-152-3p, CaM, and CaMKIIα as potential targets for tilianin treatment in 2VO rats using high-throughput sequencing analysis. **(A)** Volcano plots of differentially expressed miRNAs in the cortical tissues of 2VO rats after tilianin treatment. **(i)** Comparison between the 2VO model and sham groups. **(ii)** Comparison between the tilianin treatment and 2VO model groups. **(B)** Volcano plots of differentially expressed mRNAs in the cortical tissues of 2VO rats after tilianin treatment. **(i)** Comparison between the 2VO model and sham groups. **(ii)** Comparison between the tilianin treatment and 2VO groups. **(C)** Venn diagram of the intersection of miRNA-predicted targets and differentially expressed genes. **(D)** Network diagram of protein-protein interactions of 15 core targets. **(E)** Top 10 significantly enriched GO terms. **(F)** Top 10 significant KEGG pathway terms. **(G)** Interaction network diagram of differentially expressed miRNA and mRNA after tilianin treatment in VaD.

High-throughput sequencing of rat brains identified 13 upregulated miRNAs and 22 downregulated miRNAs in the 2VO model group (as compared to the sham group; **Figure 2Ai**). Furthermore, there were 38 upregulated miRNAs and 18 downregulated miRNAs in the tilianin-treated 2VO group compared with the 2VO group (**Figure 2Aii**). Moreover, 203 upregulated mRNAs and 602 downregulated mRNAs were detected in the 2VO model group compared with the sham group (**Figure 2Bi**), while 704 upregulated mRNAs and 192 downregulated mRNAs were identified in the tilianin treatment group (compared with the 2VO group; **Figure 2Bii**). Through analysis of the miRNAs described above, we screened 7 miRNAs (**Table S3**) and 17 mRNAs (**Table S4**) that showed opposite trends between the 2VO and tilianin-treated 2VO groups. These miRNAs and mRNAs may contribute to the molecular mechanism underlying the treatment effects of tilianin for VaD.

Using the miRDB, miRWalk, and TargetScan databases, 350 mRNAs were predicted to be the target genes of the candidate miRNAs. Fifteen common targets were obtained by merging the 17 screened potential mRNAs and predicted mRNAs, which were considered core targets closely related to tilianin functions (**Figure 2C**). A protein-protein interaction (PPI) network diagram was drawn to deeply analyze these core targets using the STRING database (**Figure 2D**). The network comprised 15 nodes (targets) and 34 edges (interactive relationships between targets). The key targets identified included *CALM1*, *CAMK2A*, *CAMK2B*, insulin-like growth factor 2 (*IGF2*), collagen α 1 (*COL1A1*), and excitatory amino acid transporter 1 (*SLC1A3*).

Next, GO and KEGG enrichments were used to analyze the biological functions and signaling pathways of the 15 core target genes. In GO terms, the biological functions focused mainly on vascular development, cardiovascular system development, vascular morphogenesis, cell adhesion regulation, etc. The cell component was concentrated primarily on the extracellular matrix, basement membrane, and contractile fibers containing collagen. The molecular functions focused on collagen binding, protein domain-specific binding, extracellular matrix structural components, and platelet-derived growth factor binding (**Figure 2E**). KEGG pathway analysis revealed that these core targets involved in the effects of tilianin were significantly enriched in some neurofunction-associated signaling pathways, namely the PI3K/AKT, Ca^2+^, neurotrophin, and MAPK signaling pathways (**Figure 2F**). Furthermore, a deep interaction network of dysregulated miRNAs and mRNAs revealed interactive relationships for miR-193b-3p, miR-152-3p, CaM, and CaMKIIα, highlighting them as potential targets for tilianin action and deserving of further investigation (**Figure 2G**).

### 3.3 Tilianin restores miR-193b-3p and miR-152-3p expression in VaD model rats

**Figure 3:**
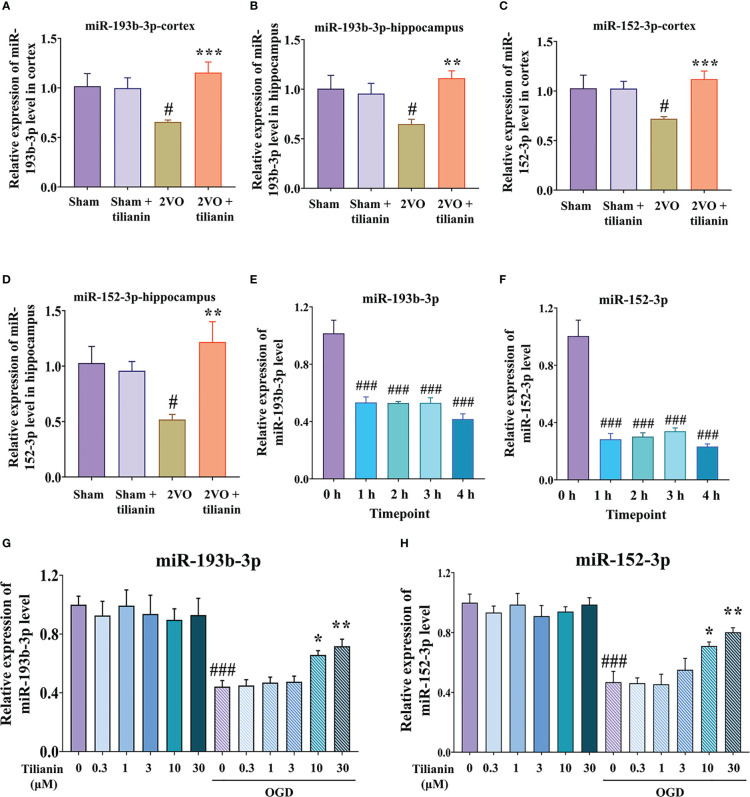
Expression of miR-193b-3p and miR-152-3p in VaD-related models. **(A, B)** Expression of miR-193b-3p in the cortex **(A)** and hippocampus **(B)** of 2VO rats. **(C, D)** Expression of miR-152-3p in the cortex **(C)** and hippocampus **(D)** of 2VO rats. **(E, F)** Expression of miR-193b-3p I and miR-152-3p **(F)** in OGD-injured cells at different time points. **(G, H)** Expression of miR-193b-3p **(G)** and miR-152-3p **(H)** in OGD-injured cells treated with tilianin. Results represent the mean ± SD, *n*=4. ^#^ *P*<0.05, ^###^ *P*<0.001 *vs.* corresponding controls, ^*^ *P*<0.05, ^**^ *P*<0.01, ^***^ *P*<0.001 *vs.* OGD or 2VO.

After determining the involvement of miR-193b-3p and miR-152-3p in the effects of tilianin against VaD, we confirmed that their expression was significantly decreased in the cortex and hippocampus of 2VO rats (**Figures 3A–D**, *P*<0.05, *P*<0.01 *vs.* sham, respectively), but increased in response to tilianin treatment (**Figures 3A–D**, *P*<0.01, *P*<0.001 *vs.* 2VO, respectively). The expression of miR-193b-3p and miR-152-3p was also reduced in OGD-injured SH-SY5Y cells at different time points (**Figures 3E, F**, all *P*<0.001 *vs.* 0 *h*), but increased by 10 μM and 30 μM tilianin treatment for 24 h (**Figures 3G, H**, *P*<0.05, *P*<0.01 *vs.* OGD, respectively); notably, there were no expression changes in control cells treated with tilianin.

### 3.4Overexpression of miR-193b-3p and miR-152-3p improves cognition and neurodegeneration in VaD model rats

**Figure 4:**
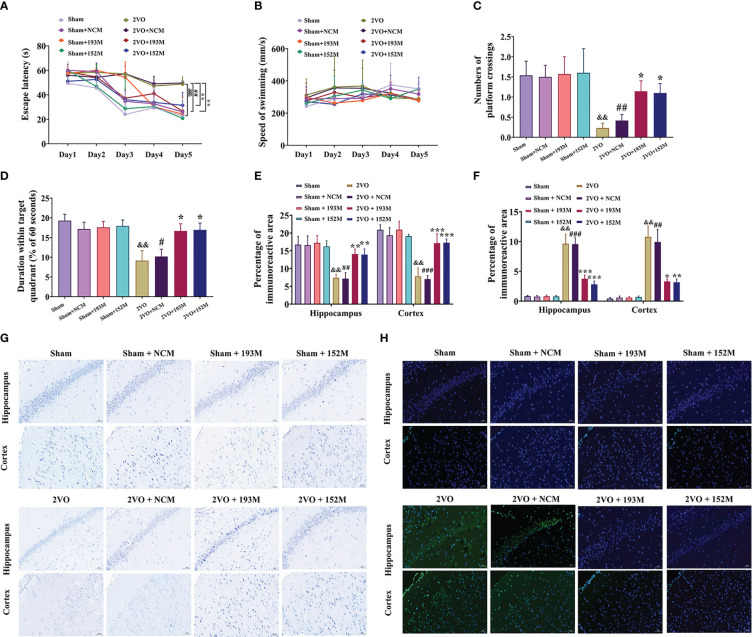
Effects of miR-193b-3p and miR-152-3p on cognitive decline and neurodegeneration in 2VO rats. **(A)** Comparison of the latency to find the platform during the five training days for the Morris water maze test (*n* = 8). **(B)** Swimming speed during the five training days (*n* = 8). **(C)** Percentage of time spent in the target quadrant in the probe test (*n* = 8). **(D)** Number of crossings where the platform was previously located in the probe test (*n* = 8). **(E)** Quantitative analysis of immunoreactivity of Nissl staining in the cerebral cortex and hippocampus of 2VO rats (*n* = 3). **(F)** Quantitative analysis of immunoreactivity of TUNEL staining in the cerebral cortex and hippocampus of 2VO rats (*n* = 3). **(G)** Representative images of Nissl-stained cortical and hippocampal neurons (*n* = 3). Scale bar = 50 μm. **(H)** Representative images of TUNEL-stained cortical and hippocampal neurons (*n* = 3). Scale bar = 50 μm. Results represent the mean ± SD, ^&&^ *P*<0.01, ^&&&^ *P*<0.001 *vs.* sham, ^#^ *P*<0.05, ^##^ *P*<0.01, ^###^ *P*<0.001 *vs.* sham+NCM, ^*^ *P*<0.05, ^**^ *P*<0.01, ^***^ *P*<0.001 *vs.* 2VO+NCM.

Subsequently, we investigated whether miR-193b-3p and miR-152-3p impacted VaD. As expected, overexpression of miR-193b-3p and miR-152-3p loaded by AAVs improved spatial learning and memory in 2VO rats (**Figures 4A, C, D**, *P*<0.05, *P*<0.01 *vs.* 2VO+NCM, respectively). There was no significant influence of miR-193b-3p and miR-152-3p on learning and memory in sham rats compared with sham rats infused with NCMs (**Figures 4A, C, D**). Motor function was not affected by the various treatments, as reflected by similar swimming speeds (**Figure 4B**).

In terms of histopathological changes, overexpression of miR-193b-3p and miR-152-3p increased the percentage area of Nissl bodies in the cortex and hippocampus of 2VO rats, indicating attenuated neuronal loss (**Figures 4E, G**, *P*<0.01, *P*<0.001 *vs.* 2VO+NCM, respectively). Similarly, TUNEL staining revealed significantly reduced apoptosis by miR-152-3p and miR-193b-3p mimics in 2VO rats (**Figures 4F, H**, *P*<0.05, *P*<0.01, *P*<0.001 *vs.* 2VO+NC, respectively). These findings suggest that upregulation of miR-193b-3p and miR-152-3p alleviates neuronal degeneration and apoptosis in VaD model rats.

### 3.5 miR-193b-3p targets CaM and miR-152-3p targets CaMKIIα directly

**Figure 5:**
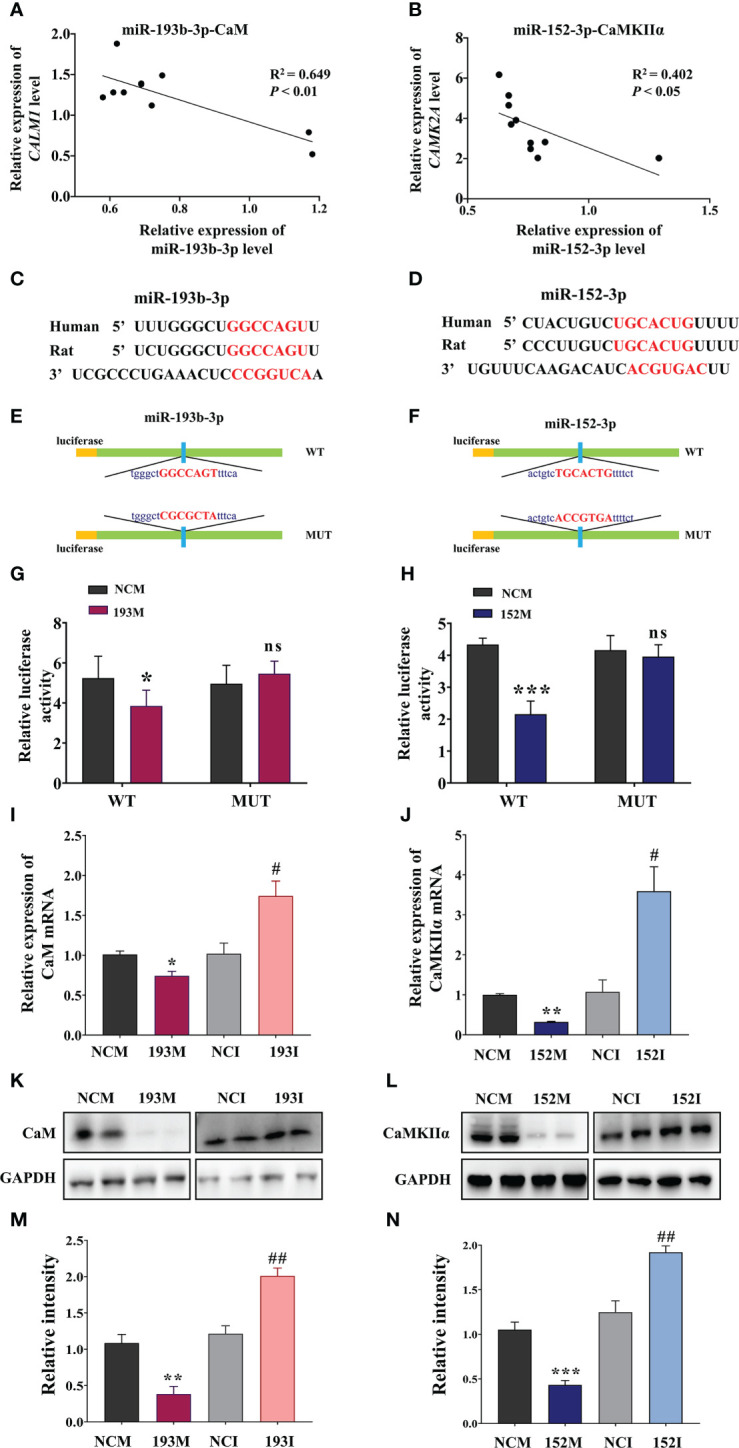
miR-193b-3p and miR-152-3p target CaM and CaMKIIɑ, respectively, by directly binding to 3′-UTR. **(A, B)** Correlation between CaM mRNA and miR-193b-3p **(A)**, as well as between CaMKIIɑ mRNA and miR-152-3p **(B)**, in the brains of 2VO rats. **(C, D)** Predicted binding sites of miR-193b-3p and CaM **(C)** and miR-152-3p and CaMKIIɑ **(D)**. **(E, F)** Construction of the Luc-CAM-WT and Luc-CAM-MUT **(E)** and Luc-CAMK2A-WT and Luc-CAMK2A-MUT **(F)** luciferase plasmids. **(G)** Relative luciferase activity for target validation of CaM. **(H)** Relative luciferase activity for target validation of CaMKIIɑ. **(I)** Expression of CaM mRNA after transfection with miR-193b-3p mimics/inhibitor. **(J)** Expression of CaMKIIɑ mRNA after transfection with miR-152-3p mimics/inhibitor. **(K, L)** Representative Western blot bands of CaM **(K)** and CaMKIIɑ **(L)**. **(M, N)** Quantitative analysis of the ratios of CaM **(M)** and CaMKIIɑ **(N)**. Results represent the mean ± SD, *n*=4, ^*^ *P*<0.05, ^**^ *P*<0.01, ^***^ *P*<0.001 *vs.* NCM, ^#^ *P*<0.05, ^##^ *P*<0.01 *vs.* NCI. NCI, Negative control inhibitor; NCM, Negative control mimics.

According to the RNA sequencing data and bioinformatics results, CaM and CaMKIIα are potential targets for the functions of miR-193b-3p and miR-152-3p in VaD. Subsequent analyses indicated that miR-193b-3p expression was negatively correlated with CaM expression, whereas miR-152-3p expression was negatively correlated with CaMKIIɑ expression, in the brains of 2VO rats (**Figures 5A, B**, *P*<0.05, *P*<0.01, R^2 =^ 0.402, R^2 =^ 0.649). The online bioinformatics analyses identified the specific binding sites of miR-193b-3p and miR-152-3p as CaM mRNA 3’-UTR and CaMKIIɑ mRNA 3’-UTR, respectively (**Figures 5C, D**). These target sequences for binding are conserved in humans, rats, mice, chimpanzees, and other mammals, etc. Based on these results, plasmids containing wild-type and mutant-type binding sites were constructed (**Figures 5E, F**). Luciferase activity significantly decreased in cells co-transfected with CALM-wild-type plasmids and miR-193b-3p mimics (**Figures 5G**, *P*<0.05 vs. NCM), as well as in cells co-transfected with CAMK2A-wild-type plasmids and miR-152-3p mimics (**Figure 5H**, *P*<0.001 vs. NCM). In contrast, there was no change in luciferase activity between miR-193b-3p/miR-152-3p mimics and NCM in cells transfected with mutant plasmids. Moreover, CaM expression at the mRNA and protein level was significantly suppressed by miR-193b-3p mimics and elevated by miR-193b-3p inhibitors (**Figures 5I, K, M**, *P*<0.05, *P*<0.01, *P*<0.001 *vs.* NCM/NCI). Similar results were observed for the effects of miR-152-3p mimics and inhibitors on CaMKIIɑ mRNA and protein expression levels (**Figures 5J, L, N**, *P*<0.05, *P*<0.01 *vs.* NCM/NCI). Taken together, these findings indicate that miR-193b-3p and miR-152-3p directly bind to and regulate CaM and CaMKIIɑ expression.

### 3.6 miR-193b-3p and miR-152-3p attenuate cognitive decline dependent on CaM- and CaMKIIɑ-mediated apoptosis and inflammation

**Figure 6:**
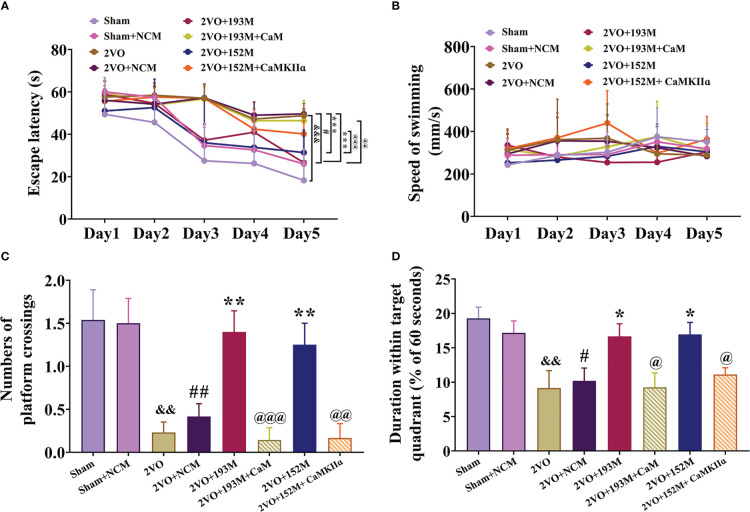
miR-193b-3p and miR-152-3p attenuated cognitive decline in VaD model rats by targeting CaM and CaMKIIɑ. **(A)** Escape latency in the Morris water maze navigation test. **(B)** Swimming speed. **(C)** Number of platform crossings. **(D)** Duration spent within the target quadrant. Results represent the mean ± SD, *n*=8, ^&&^ *P*<0.01, ^&&&^ *P*<0.001 *vs.* sham, ^#^ *P*<0.05, ^##^ *P*<0.01 *vs.* sham+NCM, ^*^ *P*<0.05, ^**^ *P*<0.01, ^***^ *P*<0.001 *vs.* 2VO+NCM, ^@^ *P*<0.05, ^@@^ *P*<0.01, ^@@@^ *P*<0.001 *vs.* 2VO+193M/152M.

Next, we explored whether the protective effects of miR-193b-3p and miR-152-3p in VaD are dependent on CaM and CaMKII targeting. The MWM results showed that miR-193b-3p- and miR-152-3p-mediated improvement in spatial learning (**Figure 6A**, *P*<0.05, *P*<0.001 *vs.* 2VO+NCM) and memory (**Figures 6C, D**, *P*<0.05, *P*<0.01 *vs.* 2VO+NCM) in 2VO rats was significantly blocked by overexpression of CaM and CaMKIIα (**Figures 6A, C, D**, *P*<0.05, *P*<0.01, *P*<0.001 *vs.* 2VO+193M/2VO+152M). No rats showed motor disability, as indicated by no differences in swimming speed between the CaM and CaMKIIα overexpression groups and other treatment groups (**Figure 6B**).

**Figure 7:**
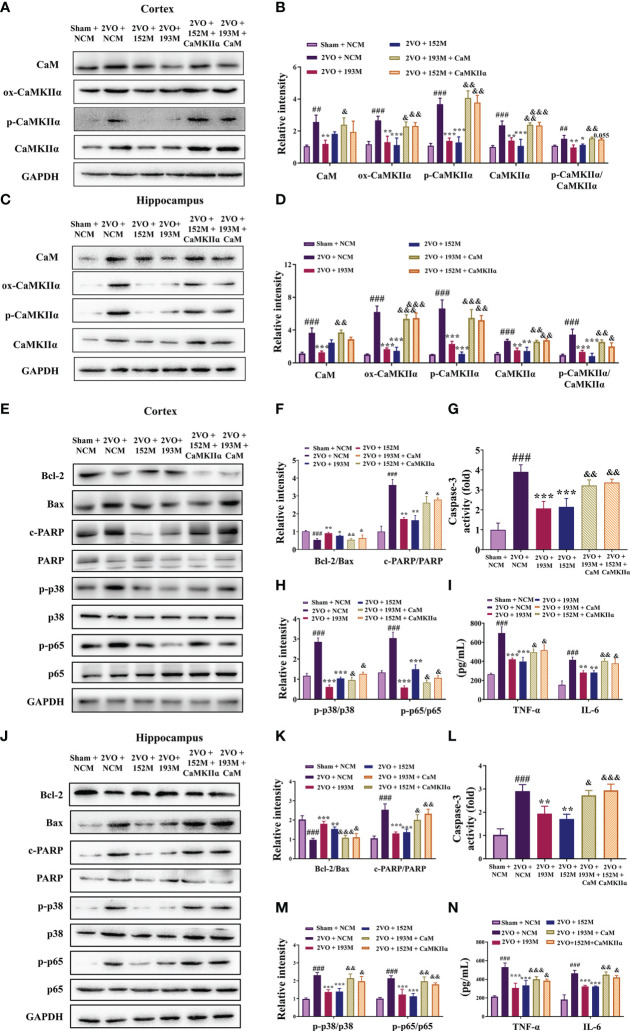
miR-193b-3p and miR-152-3p inhibited apoptosis and inflammation by targeting CaM/CaMKIIα *in vivo*. **(A, C)** Representative Western blot bands for CaM, ox-CaMKIIα, p-CaMKIIα, and CaMKIIα in the cortex **(A)** and hippocampus **(C)**. **(B, D)** Quantitative analysis of CaM, ox-CaMKIIα, p-CaMKIIα, CaMKIIα, and p-CaMKIIα/CaMKIIα in the cortex **(B)** and hippocampus **(D)**. **(E, J)** Representative Western blot bands for Bcl-2, Bax, c-PARP, total PARP, p-p38, p38, p-p65, and p65 in the cortex **(E)** and hippocampus **(J)**. **(F, K)** Quantitative analysis of Bcl-2/Bax and c-PARP/PARP in the cortex **(F)** and hippocampus **(K)**. **(G, L)** Caspase-3 activity in the cortex **(G)** and hippocampus **(L)** assessed by ELISA. **(H, M)** Quantitative analysis of p-p38/p38 and p-p65/p65 in the cortex **(H)** and hippocampus **(M)**. **(I, N)** Levels of TNF-α and IL-6 in the cortex **(I)** and hippocampus **(N)** assessed by ELISA. Results represent the mean ± SD, *n*=3, ^##^ *P*<0.01, ^###^ *P*<0.001 *vs.* sham+NC, ^*^ *P*<0.05, ^**^ *P*<0.01, ^***^ *P*<0.001 *vs.* 2VO+NC, ^&^ *P*<0.05, ^&&^ *P*<0.01, ^&&&^ *P*<0.001 *vs.* 2VO+152M/193M.

Western blot results revealed that the expression of ox-CaMKII, p-CaMKII, and CaMKIIα, along with the ratio of p-CaMKII/CaMKIIα, in the rat cortex and hippocampus were significantly suppressed by miR-193b-3p and miR-152-3p (**Figures 7A–D**, *P*<0.05, *P*<0.01, *P*<0.001 *vs.* 2VO+NC), and that such downregulation was reversed by CaM and CaMKIIα overexpression (**Figures 7A–D**, *P*<0.05, *P*<0.01, *P*<0.001 *vs.* 2VO+193M/2VO+152M). Furthermore, CaM expression was suppressed by miR-193b-3p mimics, but unaffected by miR-152-3p mimics (**Figures 7A–D**, *P*<0.05, *P*<0.01 *vs.* 2VO+NC).

Additional western blot and ELISA analyses revealed that the inhibition of apoptosis caused by miR-193b-3p and miR-152-3p was prevented by the corresponding CaM and CaMKIIα overexpression, as reflected by reduction of the increased Bcl-2/Bax ratio induced by miR-193b-3p and miR-152-3p in the cortex and hippocampus of 2VO rats and an increase in the decreased caspase-3 and PARP activity levels that had been induced by the two miRNAs (**Figures 7E–G, J–L**, *P*<0.05, *P*<0.01, *P*<0.001 *vs.* 2VO+152M/2VO+193M). Overexpression of CaM and CaMKIIα also reversed the anti-inflammatory effects of miR-193b-3p and miR-152-3p in the cortex and hippocampus of 2VO rats, as demonstrated by elevation of miRNA-induced decreased levels of p-p38/p38, p-p65/p65, TNF-ɑ, and IL-6 (**Figures 7E, H–J, M, N**, *P*<0.05, *P*<0.01 *vs.* 2VO+193M/2VO+152M).

In OGD-injured cells, miR-193b-3p mimics suppressed the expression of CaM, ox-CaMKII, p-CaMKII, and CaMKII, as well as the ratio of p-CaMKII/CaMKIIα, whereas CaM overexpression blocked these changes (**Figures S2A, B**, *P*<0.05, *P*<0.01, *P*<0.001 *vs.* 193M). The expression of ox-CaMKII, p-CaMKII, and CaMKIIα, as well as the ratio of p-CaMKII/CaMKIIα, was decreased by miR-152-3p mimics (**Figures S2E, F**, *P*<0.05, *P*<0.01, *P*<0.001 *vs.* NCM), but increased by co-transfection with miR-152-3p mimics and CaMKIIα (**Figures S2E, F**, *P*<0.05, *P*<0.01, *P*<0.001 *vs.* 152M). Moreover, the suppressed levels of p-p38/p38, p-p65/p65, TNF-ɑ, and IL-6 due to the miR-193b-3p and miR-152-3p mimics were significantly elevated by corresponding CaM and CaMKIIα overexpression (**Figures S2A, C–E, G, H**, *P*<0.05, *P*<0.01, *P*<0.001 *vs.* 193M/152M). Collectively, these results indicate that miR-193b-3p and miR-152-3p improved cognition in the VaD model rats by attenuating inflammation *via* CaM and CaMKII.

### 3.7 Tilianin ameliorates cognitive deficits and neurodegeneration in VaD model rats dependent on miR-193b-3p and miR-152-3p

**Figure 8:**
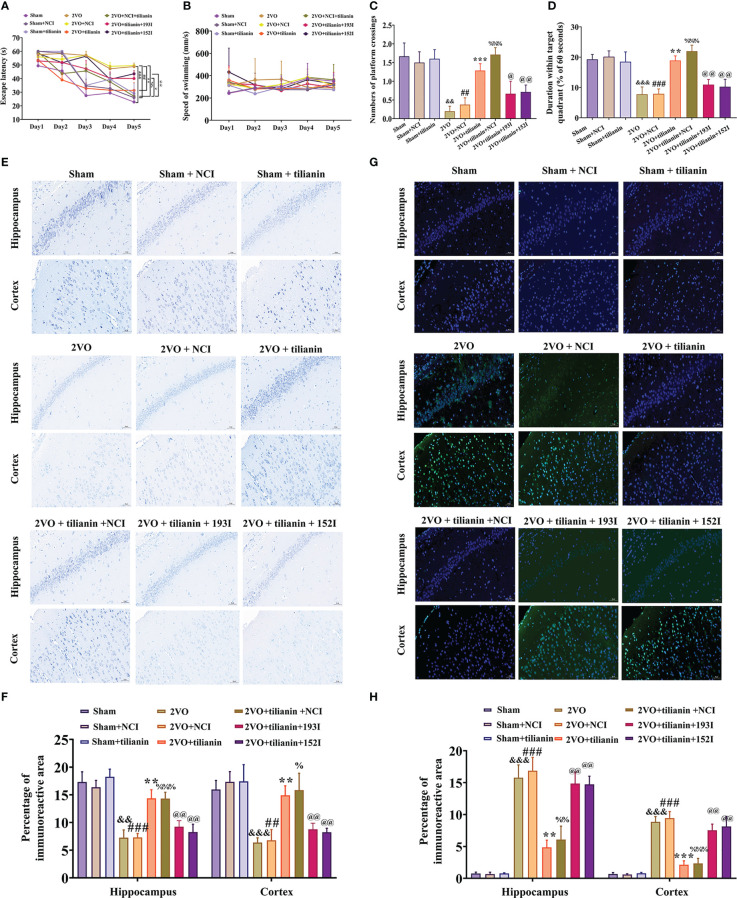
The ameliorative effects of tilianin on cognitive decline and neurodegeneration in VaD model rats were blocked by the miR-193b-3p or miR-152-3p inhibitor. **(A)** Escape latency in the Morris water maze navigation test (*n*=8). **(B)** Swimming speed in the navigation test (*n*=8). **(C)** Percentage of time spent in the target quadrant in the probe test (*n*=8). **(D)** Number of crossings where the platform was previously located in the probe test (*n*=8). **(E)** Representative images of Nissl-stained cortical and hippocampal neurons (*n*=3). Scale bar=50 μm. **(F)** Quantitative analysis of immunoreactivity of Nissl staining in the cerebral cortex and hippocampus of 2VO rats (*n*=3). **(G)** Representative images of TUNEL-stained cortical and hippocampal neurons (*n*=3). Scale bar=50 μm. **(H)** Quantitative analysis of immunoreactivity of TUNEL staining in the cerebral cortex and hippocampus of 2VO rats (*n*=3). Results represent the mean ± SD. ^&&^ *P*<0.01, ^&&&^ *P*<0.001 *vs.* sham group, ^##^ *P*<0.01, ^###^ *P*<0.001 *vs.* sham+NCI, ^**^ *P*<0.01, ^***^ *P*<0.001 *vs.* 2VO, ^%^ *P*<0.05, ^%%^ *P*<0.01, ^%%%^ *P*<0.001 *vs.* 2VO+NCI, ^@^ *P*<0.05, *P*<0.01 *vs.* 2VO+NCI+tilianin.

To verify the roles of miR-193b-3p and miR-152-3p in the anti-VaD effects of tilianin, miR-193b-3p and miR-152-3p were downregulated and behavior was then assessed. The MWM results revealed that tilianin treatment with NCI substantially improved learning (**Figure 8A**, *P*<0.01 *vs.* 2VO+NCI) and memory (**Figures 8C, D**, *P*<0.001 *vs.* 2VO+NCI) in 2VO rats. However, the miR-193b-3p or miR-152-3p inhibitor blocked tilianin-associated cognitive improvement (**Figures 8A, C, D**, *P*<0.05, *P*<0.01 *vs.* 2VO+NCI+tilianin). Non-significant changes in swimming speed between groups indicated that miR-193b-3p and miR-152-3p inhibitors had minimal effect on motor motivation (**Figure 8B**).

In the histopathological analyses, Nissl staining revealed that the miR-193b-3p or miR-152-3p inhibitor decreased the percentage area of Nissl bodies (**Figures 8E, F**, *P*<0.01 *vs.* 2VO+NCI+tilianin) in the cortex and hippocampus of 2VO rats, which had been increased by tilianin treatment. Similarly, the TUNEL staining results showed that the tilianin-associated reductions in apoptosis in 2VO rats were reversed by the miR-193b-3p and miR-152-3p inhibitor (**Figures 8G, H**, *P*<0.01 *vs.* 2VO+NCI+tilianin). Together, these findings suggest that the protective effects of tilianin against cognitive decline and neurodegeneration rely on miR-193b-3p and miR-152-3p.

### 3.8 Tilianin regulates miR-193b-3p/CaM- and miR-152-3p/CaMKII-mediated apoptotic and inflammatory pathways against VaD

**Figure 9:**
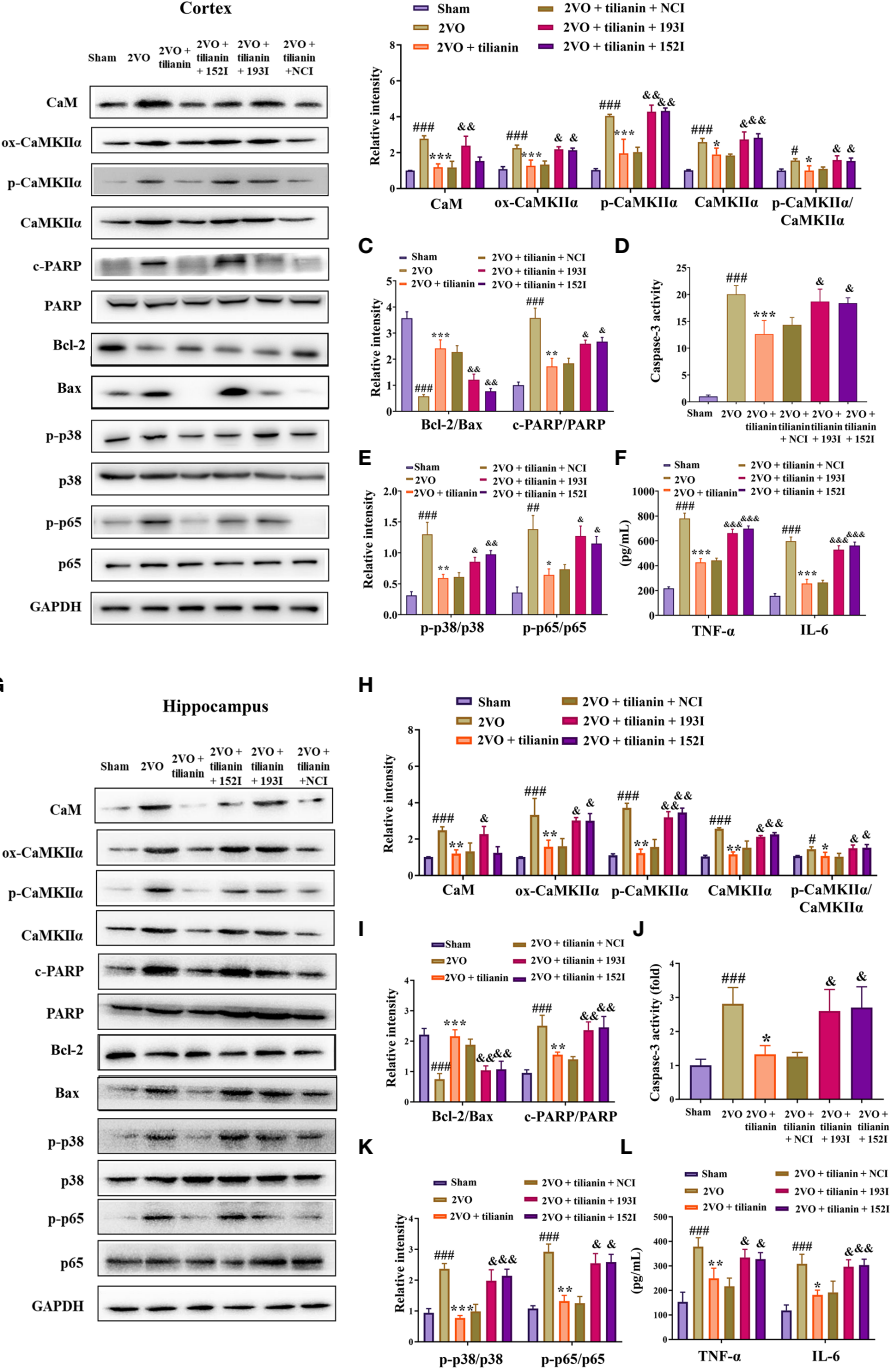
The miR-193b-3p and miR-152-3p inhibitors reversed tilianin-induced apoptotic and inflammatory inhibition *in vivo*. **(A, G)** Representative Western blot bands for CaM, ox-CaMKIIα, p-CaMKIIα, CaMKIIα, Bcl-2, Bax, c-PARP, total PARP, p-p38, p38, p-p65, and p65 in the cortex **(A)** and hippocampus **(G)**. **(B, H)** Quantitative analysis of the ratios of CaM, ox-CaMKIIα, p-CaMKIIα, CaMKIIα, and p-CaMKIIα/CaMKIIα in the cortex **(B)** and hippocampus **(H)**. **(C, I)** Quantitative analysis of the ratios of Bcl-2/Bax and c-PARP/PARP in the cortex **(C)** and hippocampus **(I)**. **(D, J)** Caspase-3 activity in the cortex **(D)** and hippocampus **(J)** assessed by ELISA. **(E, K)** Quantitative analysis of the ratios of p-p38/p38 and p-p65/p65 in the cortex **(E)** and hippocampus **(K)**. **(F, L)** Levels of TNF-α and IL-6 in the cortex **(F)** and hippocampus **(L)** assessed by ELISA. Results represent the mean ± SD, *n*=3, ^#^ *P*<0.05, ^##^ *P*<0.01, ^###^ *P*<0.001 *vs.* sham, ^*^ *P*<0.05, ^**^ *P*<0.01, ^***^ *P*<0.001 *vs.* 2VO, ^&^ *P*<0.05, ^&&^ *P*<0.01, ^&&&^ *P*<0.001 *vs.* 2VO+tilianin+NCI.

CaM expression in the cortex and hippocampus of 2VO rats was significantly decreased by tilianin; this effect was blocked by the miR-193b-3p inhibitor, but not the miR-152-3p inhibitor (**Figures 9A, B, G, H**, *P*<0.05, *P*<0.01, *P*<0.001 *vs.* 2VO or 2VO+tilianin+NCI). The expression of ox-CaMKII, p-CaMKII, and CaMKIIα, as well as the p-CaMKII/CaMKIIα ratio, in these two brain regions in 2VO rats was significantly suppressed by tilianin (**Figures 9A, B, G, H**, *P*<0.05, *P*<0.01, *P*<0.001 *vs.* 2VO); these suppressive effects were blocked by the miR-193b-3p and miR-152-3p inhibitor (**Figures 9A, B, G, H**, *P*<0.05, *P*<0.01 *vs.* 2VO+tilianin+NCI). The NC inhibitors did not affect the beneficial effects of tilianin.

Furthermore, tilianin attenuated 2VO-induced apoptosis in rats (**Figures 9A, C, D, G, I, J**, *P*<0.05, *P*<0.01, *P*<0.001 *vs.* 2VO), and these reductions were reversed by the miR-193b-3p and miR-152-3p inhibitors (**Figures 9A, C, D, G, I, J**, *P*<0.05, *P*<0.01 *vs.* 2VO+tilianin+NCI). As expected, tilianin attenuated inflammatory reactions, including inhibition of p-p38/p38, p-p65/p65, TNF-ɑ, and IL-6 levels in 2VO rats (**Figures 9A, E, F, G, K, L**, *P*<0.01, *P*<0.001 *vs.* 2VO). These effects were blocked by the miR-193b-3p inhibitor and miR-152-3p inhibitor (**Figures 9A, E–G, K, L**, *P*<0.05, *P*<0.01, *P*<0.001 *vs.* 2VO*+*tilianin+NCI).

**Figure 10:**
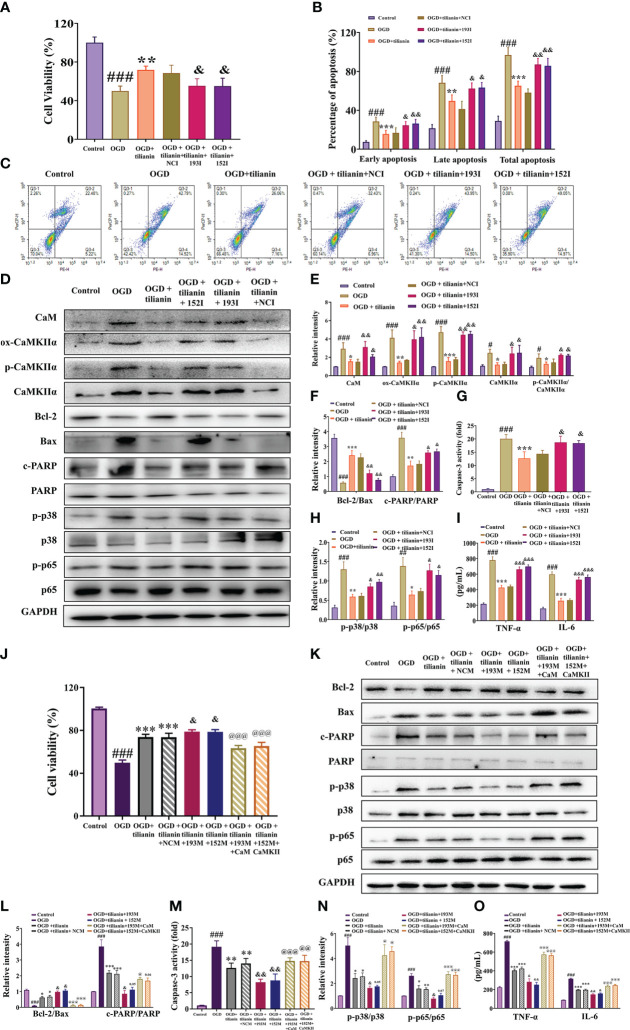
Effects of miR-193b-3p/CaM and miR-152-3p/CaMKII dysregulation on the neuroprotective effects of tilianin *in vitro*. **(A)** The miR-193b-3p and miR-152-3p inhibitors reversed the positive effect of tilianin on the viability of OGD cells. **(B)** Quantitative analysis of the proportion of apoptosis. **(C)** Representative images of the apoptosis of OGD cells among different groups. **(D)** Representative Western blot bands for CaM, ox-CaMKIIα, p-CaMKIIα, CaMKIIα, Bcl-2, Bax, c-PARP, total PARP, p-p38, p38, p-p65, and p65 in the different groups. **(E)** Quantitative analysis of the ratios of CaM, ox-CaMKIIα, p-CaMKIIα, CaMKIIα, and p-CaMKIIα/CaMKIIα **(F)** Quantitative analysis of the ratios of Bcl-2/Bax and c-PARP/PARP. **(G)** Caspase-3 activity in OGD-injured cells assessed by ELISA. **(H)** Quantitative analysis of the ratios of p-p38/p38 and p-p65/p65. **(I)** Levels of TNF-α and IL-6 in OGD-injured cells assessed by ELISA. **(J)** Effects of overexpression of miR-193b-3p, miR-152-3p, CaM, and CaMKII on the viability of OGD-injured cells. **(K)** Representative Western blot bands for Bcl-2, Bax, c-PARP, total PARP, p-p38, p38, p-p65, and p65 in OGD-injured cells. **(L)** Quantitative analysis of Bcl-2/Bax and c-PARP/PARP. **(M)** Caspase-3 activity in OGD-injured cells assessed by ELISA. **(N)** Quantitative analysis of the p-p38/p38 and p-p65/p65 ratios. **(O)** Levels of TNF-α and IL-6 in OGD-injury cells assessed by ELISA. Results represent the mean ± SD, *n*=3, ^##^ *P*<0.01, ^###^ *P*<0.001 *vs.* control, ^*^ *P*<0.05, ^**^ *P*<0.01, ^***^ *P*<0.001 *vs.* OGD, ^&^ *P*<0.05, ^&&^ *P*<0.01 *vs.* OGD+tilianin+NCI/NCM, ^@^ *P*<0.05, ^@@^ *P*<0.01, ^@@@^ *P*<0.001 *vs.* OGD+ tilianin+193M/152M.

The occlusive mechanism of miR-193b-3p/CaM and miR-152-3p/CaMKII dysregulation on the protective effects of tilianin was further investigated using OGD-injured cells. When miR-193b-3p and miR-152-3p were downregulated, the protective effect of tilianin on the viability and apoptotic rates of OGD-injured cells was significantly inhibited (**Figures 10A–C**, *P*<0.05, *P*<0.01 *vs.* OGD+tilianin+NCI). Notably, downregulation of miR-193b-3p and miR-152-3p inhibited the tilianin-induced expression decreases of CaM, ox-CaMKII, p-CaMKII, and CaMKII, along with the p-CaMKII/CaMKIIα ratio, in OGD-injured cells (**Figures 10D, E**, *P*<0.05, *P*<0.01 *vs.* OGD+tilianin+NCI). The reverse effects were observed for the apoptotic and inflammatory indicators of tilianin-treated OGD-injured cells in the presence of miR-193b-3p and miR-152-3p inhibitors, as demonstrated by the decreased Bcl-2/Bax ratio, increased caspase-3 and PARP activity levels, and increased p-p38/p38, p-p65/p65, TNF-ɑ, and IL-6 levels (**Figures 10D, F–I**, *P*<0.05, *P*<0.01, *P*<0.001 *vs.* OGD+tilianin+NCI). Upregulation of miR-193b-3p and miR-152-3p also enhanced the protective effects of tilianin on OGD-injured cells (**Figure 10J**, *P*<0.05 *vs.* OGD+tilianin+NCM). When CaM and CaMKII were overexpressed, miR-193b-3p and miR-152-3p’s enhancement of tilianin’s neuroprotective effects in OGD-injured cells was suppressed (**Figure 10J**, *P*<0.001 *vs.* OGD+tilianin+193M/152M). Similarly, CaM and CaMKII overexpression blocked the increased effects of miR-193b-3p and miR-152-3p on tilianin-mediated inflammation and apoptosis, as illustrated by activation of the p38 MAPK/NF-κB p65 and Bcl-2/Bax/caspase-3/PARP signaling cascades (**Figures 10K–O**, *P*<0.05, *P*<0.01 and *P*<0.001, *vs.* OGD+tilianin+193M/152M). Taken together, these findings suggest that tilianin exerts protective effects against VaD by specifically acting on miR-193b-3p/CaM and miR-152-3p/CaMKII, jointly inhibiting the p38 MAPK/NF-κB p65 inflammatory and Bcl-2/Bax/caspase-3/PARP apoptotic pathways.

## 4 Discussion

VaD is caused by ischemia or hemorrhagic tissue damage to specific brain regions, eventually leading to cognitive impairment (37). At present, there is a lack of effective therapeutic drugs or treatment approaches. In the present study, we found that the natural compound tilianin has protective effects against VaD and revealed that tilianin inhibits neuronal inflammation and apoptosis dependent on the miR-193b-3p/CaM and miR-152-3p/CaMKIIα co-initiated p38 MAPK/NF-κB p65 and Bcl-2/Bax/caspase-3/PARP pathways.

miRNAs are involved in various biological processes and often show unique expression profiles in disease, which may be associated with pathogenesis. In VaD, several dysregulated miRNAs have been linked to the pathological processes of inflammatory cascades, oxidative stress, neurotrophic deficiency, and neuronal apoptosis. Differentially-expressed miRNAs can be corrected using oligonucleotide-based miRNA mimics and antisense nucleotides (38). These small molecule compounds are of considerable interest due to their potential for regulating miRNA expression (39). At present, studies have only identified universal inhibitors or activators of miRNA expression (40, 41). It is thus of great importance to determine the pharmacological function and detailed mechanisms of modulators that control specific miRNAs.

With various pharmacological effects that include promoting blood circulation, removing blood stasis, clearing heat, and detoxifying, *D. moldavica* has a long history as a traditional Chinese medicine for treating cardiovascular and cerebrovascular diseases. In a previous study, we performed comprehensive miRNA profiling and constructed a compound-target gene-miRNA network of TFDM for cerebral ischemic treatment (30). We also showed that TFDM and its active compounds, namely tilianin, luteolin, and apigenin, protect against OGD injury by upregulating miR-3184-3p and downregulating miR-6875-5p in neuronal cells (30). These findings provide a foundation that dysregulated miRNAs play a role in the therapeutic effect of TFDM and its active components against brain ischemia.

Tilianin is the principal and reference compound extracted from TFDM and a prospective agent for treating cardiovascular and cerebrovascular diseases, as supported by major scientific projects in China (9). Tilianin may offer various useful functions for treating cardiovascular diseases. For example, tilianin maintains the function of OGD/R-damaged heart by inhibiting apoptotic and inflammatory reactions (34) and reduces apoptosis in ischemia-induced acute kidney injury by inhibiting ERK/EGR1/BCL2L1 pathways (38). We previously demonstrated the cardio- and neuro-protection of tilianin against ischemia *via* the p-CaMKII and ox-CaMKII signaling pathways (9, 18, 24). Permeability of the blood-brain barrier provides a possibility for tilianin to treat central nervous system diseases (**Figure S3**). In the present study, chronic cerebral hypoperfusion-induced cognitive impairment in rats was used as a standard model of VaD (39, 40). Oral administration of tilianin significantly improved spatial learning and memory impairment and neurodegenerative changes in these 2VO rats. Therefore, we suggested that tilianin could improve vascular cognitive deficits. As predicted, dependent on CaM/CaMKII axis, tilianin reduced the inflammatory response through the p38 MAPK/NF-κB pathway and apoptotic rates by inhibiting the Bcl-2/Bax/caspase-3/PARP pathway.

Ca/CaM is the primary activator of CaMKII. CaMKII is a central mediator of calcium handling, glutamate signaling, peroxidation stress, phosphorylation-dependent activation, transcription, and other processes crucial for brain function (41). When the Ca^2+^/CaM complex binds to the regulatory region of CaMKII during exposure to ischemic stimuli, CaMKII exposes the catalytic region and relieves the self-inhibitory region on the activation site (42). Subsequently, the Thr286 site of CaMKII undergoes autophosphorylation and generates spontaneous activity (43), and an oxidation-activated form results by modifying M281/282 (44). Activation of the oxidative and phosphorylated forms of CaMKIIα regulates downstream proteins involved in apoptotic and inflammatory signaling that contribute to ischemia (45). Early cellular changes during apoptosis are related to mitochondrial modifications mediated by the Bcl-2 protein family (46). Damaged mitochondria have been shown to activate downstream caspase-3 as an apoptotic executor, as well as PARP and its downstream endonucleases, ultimately leading to cell death (47). p38 MAPK is a member of the MAPK family of serine/threonine protein kinases that are conserved in eukaryotes and closely associated with cellular inflammation and apoptosis relevant to CaMKII activation. p38 MAPK further activates NF-κB to induce the synthesis of pro-inflammatory mediators, including cytokines, subsequently triggering the inflammatory response (8, 48).

Our previous studies demonstrated that tilianin has a weak regulatory effect on CaMKII protein molecules but a specific inhibitory effect on ox-CaMKII and p-CaMKII expression, resulting in inhibition of its kinase activity-dependent mechanisms (9, 18, 24). In the present study, CaM and CaMKII expression levels were increased in the VaD model and decreased in response to tilianin treatment. We speculate that post-transcriptional modifications might contribute to tilianin’s neuroprotective effects on the regulation of CaM/CaMKII signaling transduction.

Using high-throughput sequencing analysis, we explored miRNAs that responded to tilianin treatment in the VaD model; these miRNAs are thought to regulate post-transcriptional gene expression involved in the CaMKII cascades. Based on the results for 2VO rats with or without tilianin treatment, we focused on miR-193b-3p and miR-152-3p, which were part of the close interaction network within the CaM/CaMKII signaling pathway. The downregulation of miR-193b-3p and miR-152-3p in 2VO rats was significantly upregulated by tilianin treatment. The ability of decreased miR-193b-3p and miR-152-3p levels to be increased by tilianin was further validated by qRT-PCR analysis in various VaD-associated models, including OGD-injured cells and the cortex and hippocampus of 2VO model rats. In the functional experiments, overexpression of miR-193b-3p and miR-152-3p attenuated cognitive impairment and neuronal damage in the pathological VaD rat model. Prior studies have shown that exosomes containing miR-193b-3p promote neuroprotective effects and anti-inflammatory responses in mice suffering from subarachnoid hemorrhage (49). miR-193b-3p has also been shown to reduce oxidative stress and 5-lipoxygenase-induced inflammation in rats with cerebral ischemia/reperfusion injury (50). A decreased serum miR-152-3p level is a potential risk factor for cerebral infarction (51), and miR-152-3p shows protective effects against OGD injury *in vitro* (52). Our findings further support miR-193b-3p and miR-152-3p as potential pharmacological targets of tilianin action for the treatment of VaD.

To shed light on the underlying mechanisms of miR-193b-3p and miR-152-3p in tilianin treatment, this study identified miRNA target genes and molecular signaling pathways affected by the presence of tilianin. Using RNA sequencing analysis with online software and luciferase reporter assays, we found that CaM was the target of miR-193b-3p and CaMKIIα was the target of miR-152-3p with direct binding to the 3’-UTR regions. Moreover, qPCR and Western blot analyses revealed that overexpression of miR-193b-3p decreased CaM expression and inhibited activation of the CaM/CaMKII-induced p38/NF-κB inflammatory pathway and Bcl-2/Bax/caspase-3/PARP apoptotic pathway. Conversely, inhibition of miR-193b-3p led to upregulation of CaM and increased activation of inflammatory and apoptotic pathways. Similarly, overexpression of miR-152-3p inhibited the expression of CaMKIIɑ and its two isoforms, namely ox-CaMKIIα and p-CaMKIIα, suppressing subsequent signaling transduction of the p38 MAPK/NF-κB and Bcl-2/Bax/caspase-3/PARP pathways, which were unavailable with inhibition of miR-152-3p. Overexpression of CaM and CaMKIIɑ blocked the rescue of cognitive decline and neurodegeneration induced by miR-193b-3p and miR-152-3p, respectively, which was associated with activation of the downstream p38/NF-κB and Bcl-2/Bax/caspase-3/PARP pathways. These findings suggest that CaM and CaMKIIα are the target genes of miR-193b-3p and miR-152-3p and are involved in the inflammatory and apoptotic signaling pathways in VaD pathology.

**Figure 11:**
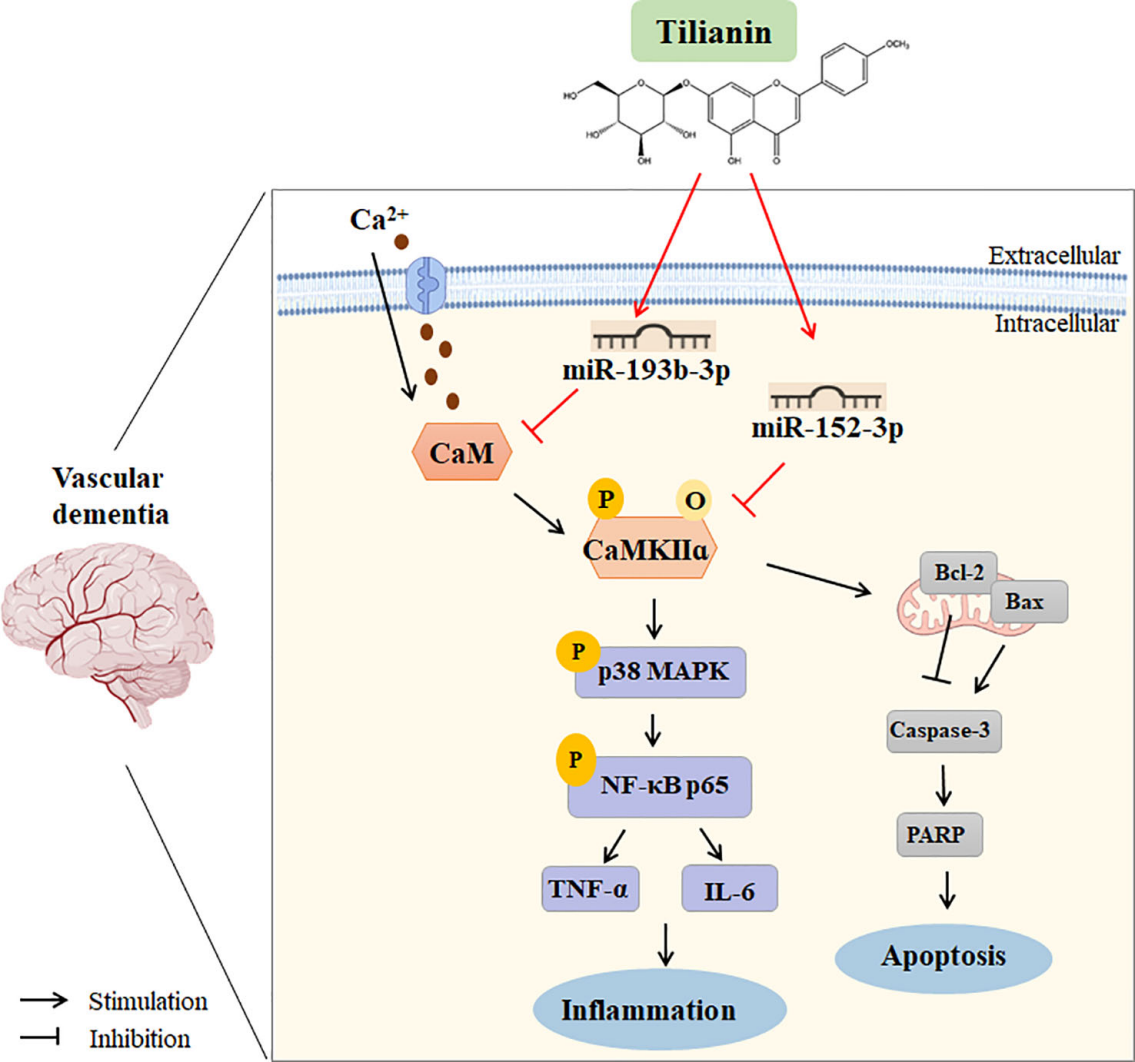
Working model of using tilianin to treat VaD. Bax: B cell lymphoma-2 associated X protein; Bcl-2: B cell lymphoma-2; CaM: calmodulin; CaMKII: Ca^2+^/calmodulin-dependent protein kinase II; Caspase-3: cysteine-dependent aspartate-specific proteases-3; IL-6: interleukin-6; NF-κB: nuclear factor kappa-B; TNF-α: tumor necrosis factor-alpha.

With clarification of the specific signaling pathways mediated by miR-193b-3p and miR-152-3p, gain- and loss-of-key target analyses were performed to understand the underlying mechanism of tilianin treatment in VaD. Oriented administration of miR-193b-3p and miR-152-3p inhibitors to the brains of 2VO rats abolished tilianin’s effects on cognitive behavior and neuronal degeneration. Furthermore, overexpression of CaM and CaMKIIα abolished the ability of miR-193b-3p and miR-152-3p to enhance the protective effect of tilianin against ischemic injury. Importantly, as illustrated by further western blot analyses, the reversal effect of CaM and CaMKIIα on the combination of tilianin with miR-193b-3p or miR-152-3p was associated with activation of the p38 MAPK/NF-κB inflammatory pathways and Bcl-2/Bax/caspase-3/PARP apoptotic pathways. These findings suggest that an imbalance of CaM/CaMKII signaling in VaD could be corrected by tilianin-based miRNA regulation (**Figure 11**). Nevertheless, we cannot rule out possible roles of tilianin in the interaction between the miR-193b-3p/CaM and miR-152-3p/CaMKIIα axes. Further experiments are needed to explore this possibility.

Although CaMKII is thought to be required for hippocampal long-term potentiation and spatial learning, excess CaMKIIα expression could also be detrimental in the case of VaD, due to deteriorating the neuroinflammation and apoptosis cascades *via* increasing phosphorylated and oxidative CaMKII expression. Tilianin treatment and miRNA overexpression reduce CaMKIIα expression levels close to those of sham rats, which can be understood as that tilianin-based miRNA regulation inhibits inflammation and apoptosis by restoring the balance of CaM/CaMKII signaling. Numerous neurons are lost in the hippocampal cornu amonis (CA) areas 1 subfield in AD, and CaMKIIα expression was increased in the remaining pyramidal neurons of this region. This increased expression may critically contribute to tau protein hyperphosphorylation and other neurodegenerative processes, such as caspase-3 hyperactivation, in CA1 pyramidal neurons. CA3 pyramidal neurons and granule cells of the dentate gyrus show an increased distribution of p-CaMKIIα. This change is suggested to shift CaMKII activity from the synapse to soma leading to synaptic deficits, neurodegenerative processes, and impaired memory formation (53). In such cases, the effect of tilianin on the rebalancing of CaMKIIα distribution and expression in the hippocampus or other regions may be a potential mechanism.

## 5 Conclusion

Taken together, the results of this study have demonstrated that tilianin attenuates cognitive impairment in VaD by regulating miR-193b-3p/CaM and miR-152-3p/CaMKIIα mediated p38 MAPK/NF-κB inflammatory and Bcl-2/Bax/caspase-3/PARP apoptotic pathways. These findings provide insights into the potential use of tilianin for treating VaD and shed light on its role as a promising small-molecule regulator of miRNAs associated with the inflammatory response.

## Data availability statement

The data presented in the study are deposited in the Gene Expression Omnibus (GEO) repository, accession number GSE228494 (https://www.ncbi.nlm.nih.gov/geo/query/acc.cgi?acc=GSE228494).

## Ethics statement

The animal study was reviewed and approved by The Experimental Animal Care and Use Committee of the Institute of Medicinal Biotechnology.

## Author contributions

RL and ZL contributed to the conception and design of the study. TS, ML, and LT performed the validation and investigation. LT provided the compounds. ML and LZ organized the figures. KZ and ZC performed the statistical analysis. SS performed identification and quantification of the compound. TS wrote the first draft of the manuscript. RL reviewed and supervised of the manuscript. All authors contributed to the article and approved the submitted version.
