# Supplementary material for: Tilianin improves cognition in a vascular dementia rodent model by targeting miR-193b-3p/CaM- and miR-152-3p/CaMKIIα-mediated inflammatory and apoptotic pathways
Source: Front Immunol. 2023 Apr 19;14:1118808. doi: 10.3389/fimmu.2023.1118808 (PMC10155197; doi:10.3389/fimmu.2023.1118808)
Supplement: Supplementary file 1 [file DataSheet_1.pdf]

Supplementary Material

Tilianin improves cognition in a vascular dementia rodent model by targeting miR-193b-3p/CaM- and miR-152-3p/CaMKIIα-mediated inflammatory and apoptotic pathways

Ting Sun[†], Linjie Tan[†], Mimin Liu[†], Li Zeng, Kaiyue Zhao, Zhongdi Cai, Shengnan Sun, Zhuorong Li*, Rui Liu*

*** Correspondence:** Rui Liu (liurui@imb.pumc.edu.cn, +86 10 67087731); Zhuorong Li (lizhuorong@imb.pumc.edu.cn, +86 10 83152017)

1 Supplementary Figures and Tables

1.1 Supplementary Tables

Table S1. The antibodies used for Immunohistochemistry and Western blot analysis

Primary Antibody	Dilution	Source
**Anti-IBA1 rabbit mAb**	1:500	Abcam
**Anti-GFAP rabbit mAb**	1:500	Abcam
**Anti-Bax rabbit mAb**	1:1000	Abcam
**Anti-Bcl-2 rabbit pAb**	1:500	Proteintech
**Anti-cleaved PARP rabbit mAb**	1:1000	Abcam
**Anti-PARP rabbit mAb**	1:1000	CST
**Anti-phosph-p38 MAPK(Thr183/Thy182) rabbit mAb**	1:1000	Abcam
**Anti-p38 MAPK rabbit pAb**	1:1000	Abcam
**Anti-phosph-NF-κB p65(Ser536) rabbit mAb**	1:1000	Abcam
**Anti-NF-κB p65 rabbit mAb**	1:1000	Abcam
**Anti-GAPDH rabbit pAb**	1:5000	Proteintech
**Anti-oxidized-CaMKIIα (Met281/282) rabbit pAb**	1:1000	GeneTex
**Anti-CaMKIIα rabbit mAb**	1:1000	Abcam
**Anti-CaM rabbit mAb**	1:1000	CST

**Goat anti-Mouse IgG (H+L)-HRP secondary antibody ZB-2305**	1: 5000 for WB<br>1:500 for IHC	ZSGB-BIO
**Goat anti-Rabbit IgG (H+L)-HRP secondary antibody ZB-2306**	1: 5000 for WB<br>1:500 for IHC	ZSGB-BIO

Table S2. PCR primer sequences

Primer Name	Sequence
**miR-152-3p-RT**	5'- GTCGTATCCAGTGCAGGGTCCGAGGT ATTCGCACTGGATACGACCCAAGT - 3'
**miR-152-3p-F**	5'- CGCGTCAGTGCATGACAGA - 3'
**miR-152-3p-R**	5'-AGTGCAGGGTCCGAGGTATT - 3'
**miR-193b-3p-RT**	5'- GTCGTATCCAGTGCAGGGTCCGAGGT ATTCGCACTGGATACGACAGCGGG - 3'
**miR-193b-3p-F**	5'- AAAGTCCCGCTCCCGCT - 3'
**miR-193b-3p-R**	5'- AGTGCAGGGTCCGAGGTATT - 3'
**U6-RT**	5'- GTCGTATCCAGTGCAGGGTCCGAGGT ATTCGCACTGGATACGACAAAATA - 3'
**U6-F**	5'- CAAATTCGTGAAGCGTTCCA - 3'
**U6-R**	5'- AGTGCAGGGTCCGAGGTATT - 3'
**miR-106a-5p-RT**	5'- GTCGTATCCAGTGCAGGGTCCGAGGT ATTCGCACTGGATACGACCTACCT - 3'
**miR-106a-5p-F**	5'- CGCGAAAAGTGCTTACAGTGC - 3'
**miR-106a-5p-R**	5'- AGTGCAGGGTCCGAGGTATT - 3'
**miR-17-5p-RT**	5'- GTCGTATCCAGTGCAGGGTCCGAGGT ATTCGCACTGGATACGACCTACCT - 3'
**miR-17-5p-F**	5'- GCGCAAAGTGCTTACAGTGC - 3'
**miR-17-5p-R**	5'- AGTGCAGGGTCCGAGGTATT - 3'
**CAMK2A-F**	5'- TTCTCTGTTTGCACTCGGCA - 3'
**CAMK2A-R**	5'- CAGGTGAGGCTTGGGACTG - 3'
**CALM1-F**	5'- CTTCCAGTCCGCAGAGAGATG - 3'

**CALM1-R**	5’- GTGGAAAACACACCCAGCGA - 3’
**GAPDH-F**	5’- ACAGTCAGCCGCATCTTCTT - 3’
**GAPDH-R**	5’- ATCCGTTGACTCCGACCTTC - 3’
**Negative control mimics**	Sense: 5’- UUGUACUACACAAAAGUACUG - 3’<br>Antisense: 5’- GUACUUUUGUGUAGUACAAUU - 3’
**miR-152-3p mimics**	Sense: 5’- UCAGUGCAUGACAGAACUUGG - 3’<br>Antisense: 5’- AAGUUCUGUCAUGCACUGAUU - 3’
**miR-193b-3p mimics**	Sense: 5’- AACUGGCCCUCAAAGUCCCGCU- 3’<br>Antisense: 5’- CGGGACUUUGAGGGCCAGUUUU - 3’
**Negative control inhibitor**	5’- CAGUACUUUUGUGUAGUACAA - 3’
**miR-152-3p inhibitor**	5’- CCAAGUUCUGUCAUGCACUGA - 3’
**miR-193b-3p inhibitor**	5’- AGCGGGACUUUGAGGGCCAGUU - 3’

Table S3. miRNAs with specific changes and opposite trends after tilianin treatment

miRNA name	Log_2(Fold Change) (C *vs.* V)	*P*-Value (C *vs.* V)	Regulated (C *vs.* V)	Log_2(Fold Change) (T *vs.* V)	*P*-Value (T *vs.* V)	Regulated (T *vs.* V)
**miR-10a-5p**	-1.0592	0.0004	down	6.4761	8.13E-05	up
**miR-10b-5p**	-1.4550	8.43E-05	down	5.7979	0.0003	up
**miR-122-5p**	3.9551	1.75E-05	up	-3.1159	0.0024	down
**miR-214-3p**	-1.2806	0.0017	down	1.7432	0.0023	up
**miR-223-3p**	-1.0494	0.0104	down	1.5156	0.0206	up
**miR-152-3p**	-3.5707	0.0285	down	3.2506	0.0305	up
**miR-193b-3p**	-4.3964	0.0198	down	2.9718	0.0493	up

Table S4. mRNAs with specific changes and opposite trends after tilianin treatment

mRNA name	Log_2(Fold Change) (C *vs.* V)	*P*-Value (C *vs.* V)	Regulated (C *vs.* V)	Log_2(Fold Change) (T *vs.* V)	*P*-Value (T *vs.* V)	Regulated (T *vs.* V)
**SLC1A3**	18.6526349	4.88E-30	up	-18.652635	4.43E-30	down
**NFIA**	2.8559291	5.05E-26	up	-3.0898423	4.24E-10	down
**CAMK2A**	3.376916	0.0269	up	-5.941051	0.01823	down
**CALM1**	4.559787	0.03703	up	-4.715904	0.02647	down
**EHMT2**	-8.2103669	5.25E-05	down	7.65329295	1.17E-09	up
**CAMK2B**	-5.0288051	3.90E-19	down	3.9081209	1.74E-05	up
**IGF2**	-2.4459694	2.64E-07	down	2.27066779	2.82E-05	up
**COL3A1**	-2.1826125	3.03E-08	down	1.89814492	1.70E-05	up
**SLC22A6**	-2.0984826	1.11E-08	down	1.4884997	1.55E-06	up

**COL1A1**	-2.0134175	9.60E-10	down	1.50381487	6.09E-08	up
**COCH**	-1.8228717	9.39E-11	down	1.48148552	3.25E-05	up
**PCOLCE**	-1.720373	1.56E-05	down	1.50536921	1.47E-06	up
**SERPING1**	-1.6162913	6.66E-08	down	1.70964857	7.47E-06	up
**GJB2**	-1.5734209	1.05E-06	down	1.48959479	7.11E-07	up
**CD74**	-1.5108106	7.74E-05	down	1.56215911	1.54E-07	up
**CRABP2**	-1.4748539	2.23E-05	down	1.35877498	4.34E-05	up
**BGN**	-1.3702454	1.07E-10	down	1.06572292	9.24E-07	up

1.2 Supplementary Figures

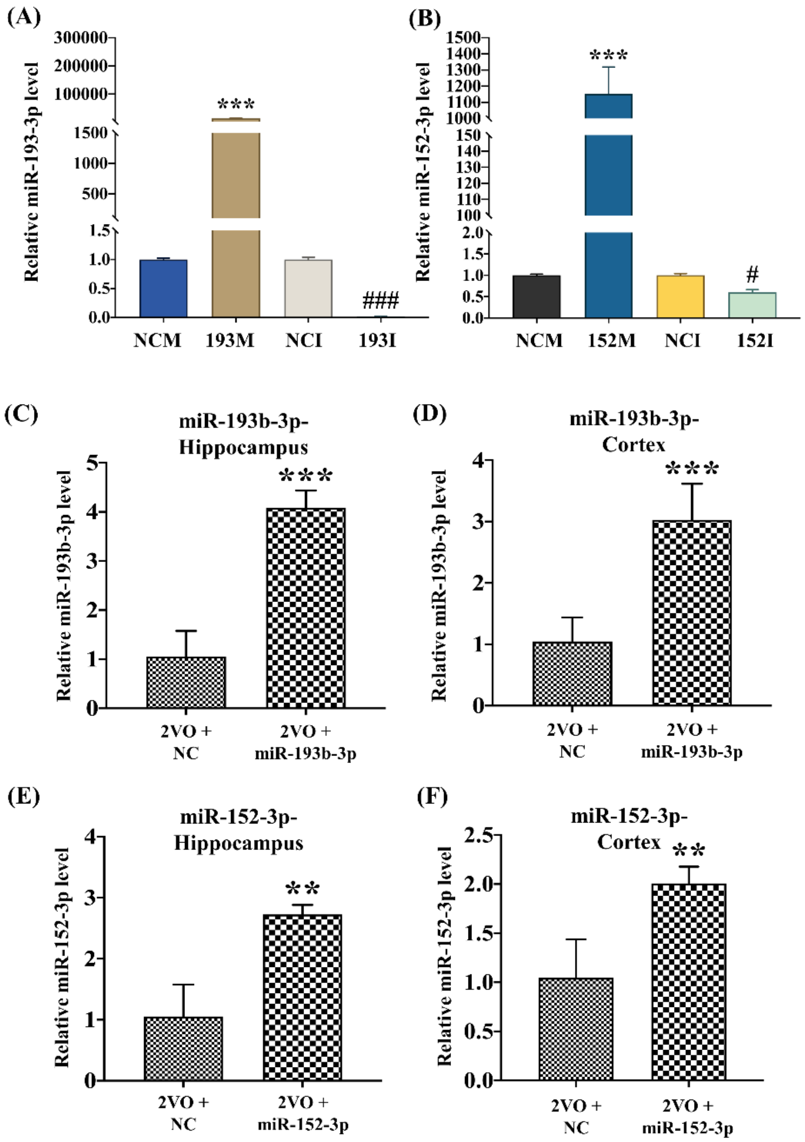

Supplementary Figure 1. Expression of miR-152-3p and miR-193b-3p after transfection with miRNAs *in vitro and in vivo*. (A) miR-193b-3p level after transfection of miR-193b-3p mimics and inhibitor detected by qRT-PCR. (B) miR-152-3p level after transfection of miR-152-3p mimics and inhibitor detected by qRT-PCR. (C-D) miR-193b-3p upregulation in the hippocampus (C) and cortex (D) of AAV-miR-193b-3p-treated 2VO rats. (E-F) miR-152-3p upregulation in the hippocampus (E)

and cortex (F) of AAV-miR-152-3p-treated 2VO rats. Results are expressed as mean ±SD, $n = 3$. $^{***}P < 0.001$ *vs*. NCM or 2VO+NC, $^{\#}P < 0.05$, $^{\#\#\#}P < 0.001$ *vs*. NCI group.

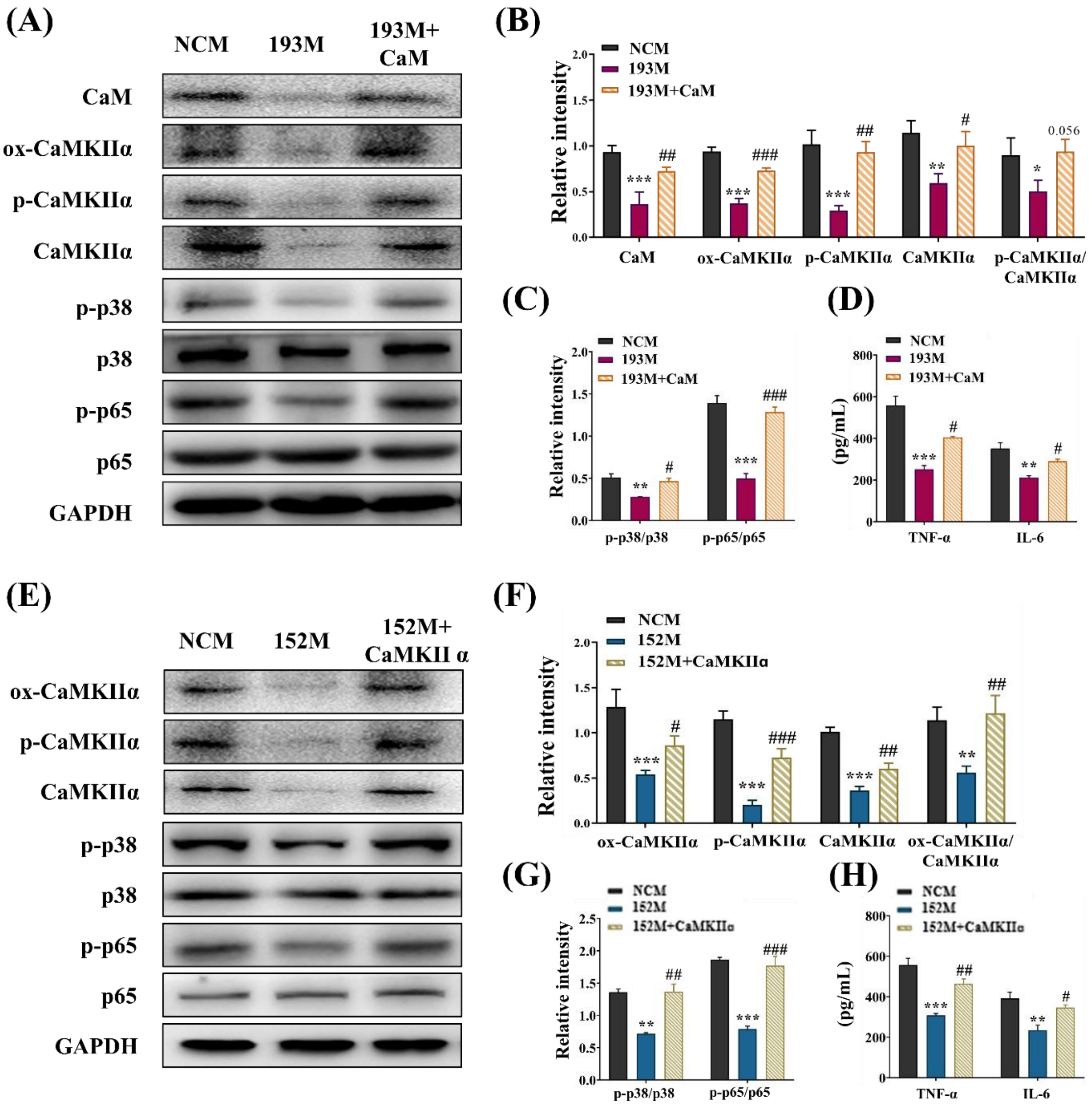

Supplementary Figure 2. miR-193b-3p and miR-152-3p inhibited inflammation by targeting CaM/CaMKIIα *in vitro*. (A) Representative Western blot bands for CaM, ox-CaMKIIα, p-CaMKIIα, CaMKIIα, p-p38, p38, p-p65, and p65. (B) Quantitative analysis of ratios of CaM, ox-CaMKIIα, p-CaMKIIα, CaMKIIα and p-CaMKIIα/CaMKIIα. (C) Quantitative analysis of ratios of p-p38/p38 and p-p65/p65. (D) The level of TNF-α and IL-6. (E) Representative Western blot bands for ox-CaMKIIα, p-CaMKIIα, CaMKIIα, p-p38, p38, p-p65, and p65. (F) Quantitative analysis of ratios of ox-CaMKIIα, p-CaMKIIα, CaMKIIα and p-CaMKIIα/CaMKIIα. (G) Quantitative analysis of ratios of p-p38/p38 and p-p65/p65. (H) The level of TNF-α and IL-6. Results represent means ± SD, $n = 4$, $^{*}P < 0.05$, $^{**}P < 0.01$, $^{***}P < 0.001$ *vs*. NCM group, $^{\#}P < 0.05$, $^{\#\#}P < 0.01$, $^{\#\#\#}P < 0.001$ *vs*. 193M or 152M group.

(A)

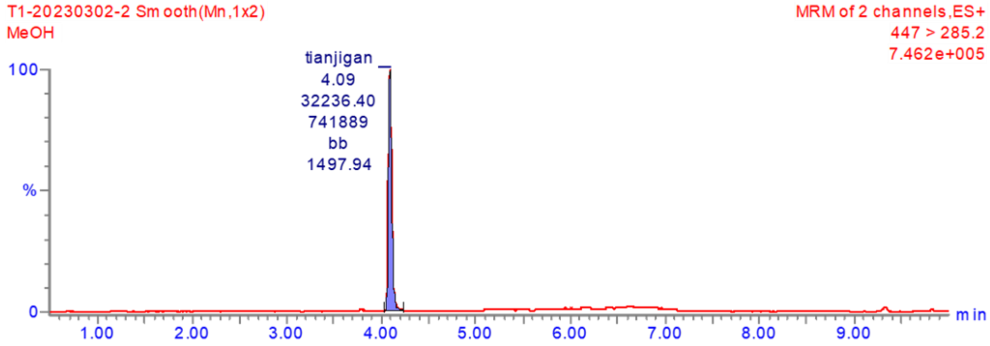

(B)

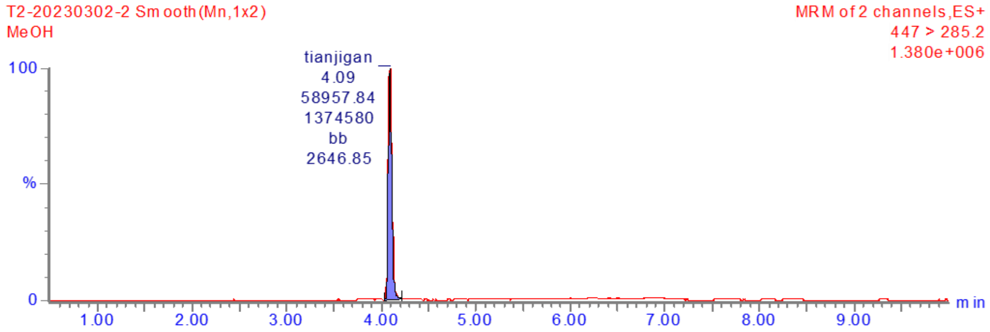

(C)

Name	Concentration (ng/ml)	Area	Results (Original concentration)
Standard-1	0.78125	4664.843	
Standard-2	1.5625	8677.512	
Standard-3	3.125	16624.97	
Standard-4	6.25	35345.5	
Standard-5	12.5	70101.81	
Standard-6	25	138899.4	
Brain (Dilution ratio 1000)		32236.402	5.78 μg/ml
Plasma (Dilution ratio 1000)		58957.844	10.58 μg/ml

Supplementary Figure 3. Tilianin level in the brain and blood of rats. (A) Tilianin level in the brain of a rat by UPLC-MS/MS (Xevo TQ-S, Waters^{TM}, USA). (B) Tilianin level in the plasma of a rat by UPLC-MS/MS. The rat was injected with 60 mg/kg tilianin or saline via the tail vein. The blood was collected from the orbit 10 mins after injection, and the whole brain was collected after the rats were sacrificed by cervical dislocation. Plasma was collected by centrifugation with 4000 ×g for 10 mins and diluted with methanol. Rat brain tissue was homogenized in the methanol solution and the supernatant was obtained after centrifugation with 5000 ×g for 10 mins. The plasma and supernatants of the brain were diluted 1000 times with methanol for assays. The levels of tilianin in the brain and plasma were calculated by the standard curve drawn by standards.

Supplementary Figure 4. Original images of Western blotting analysis.

Images in Figure 8 A

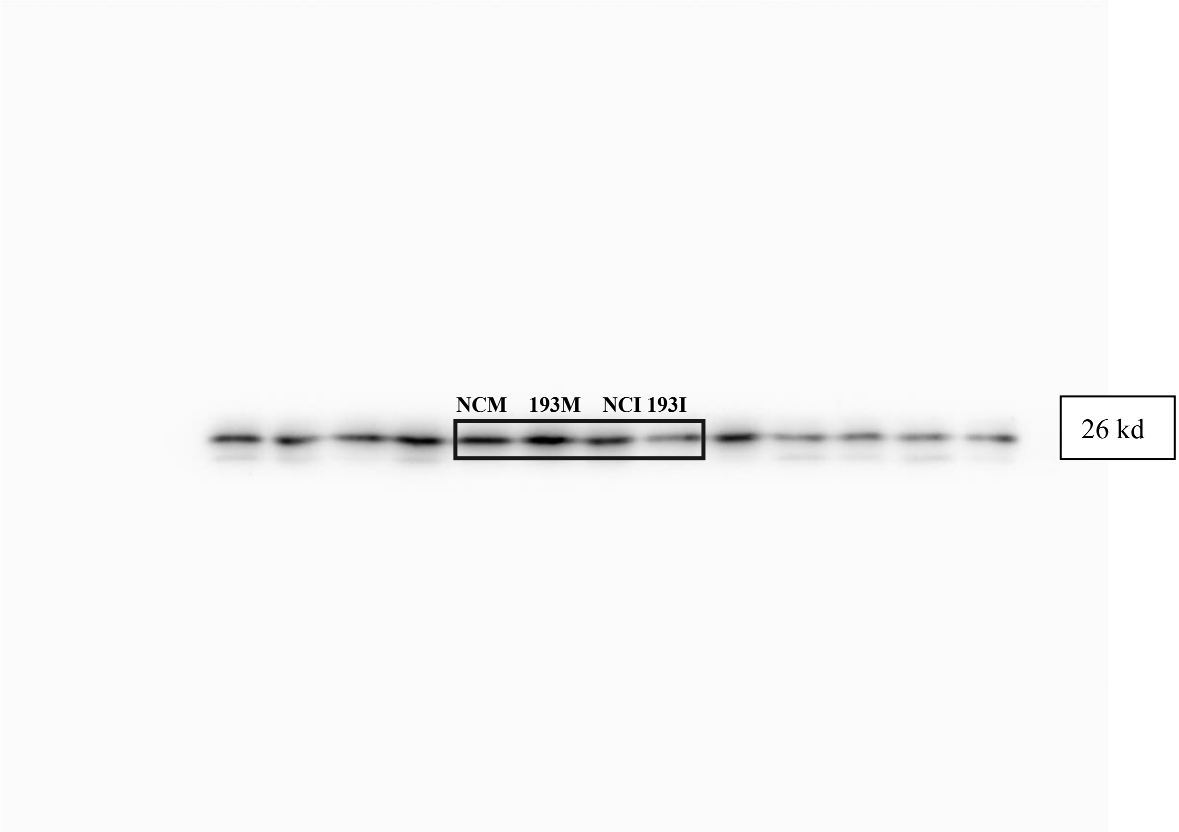

Bcl-2

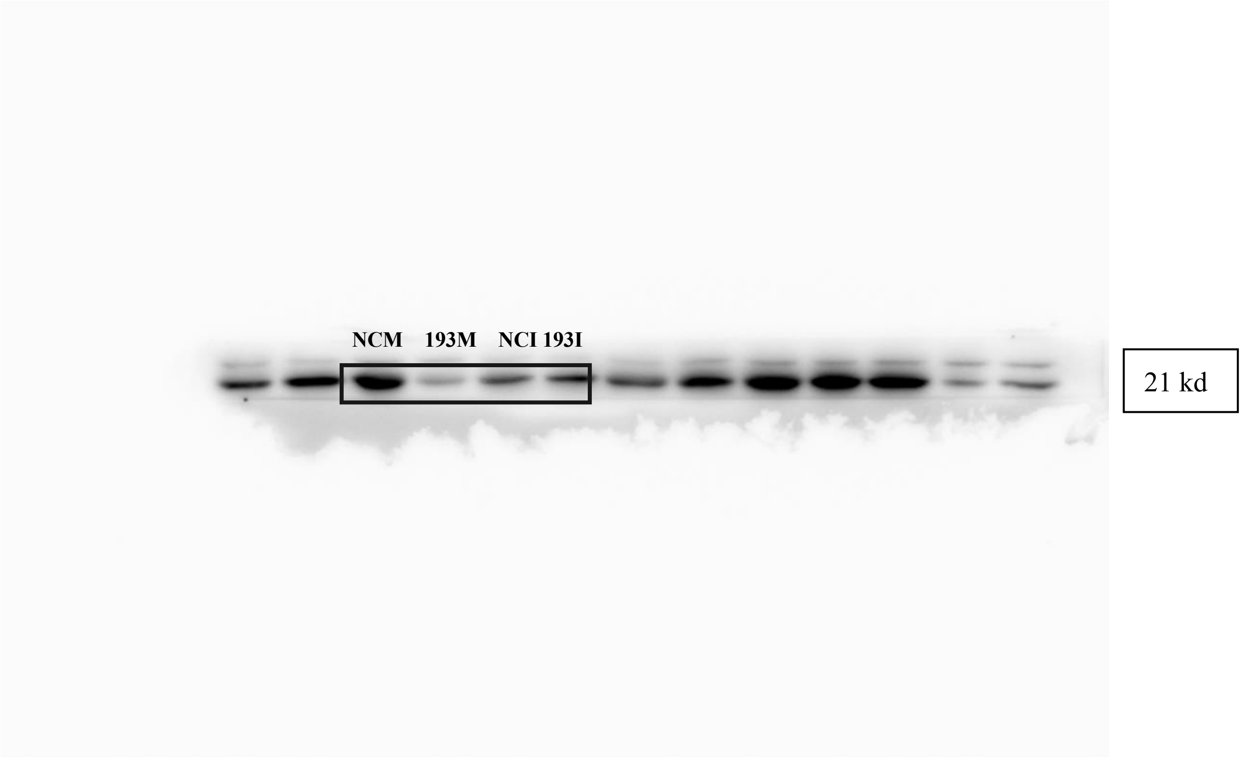

Bax

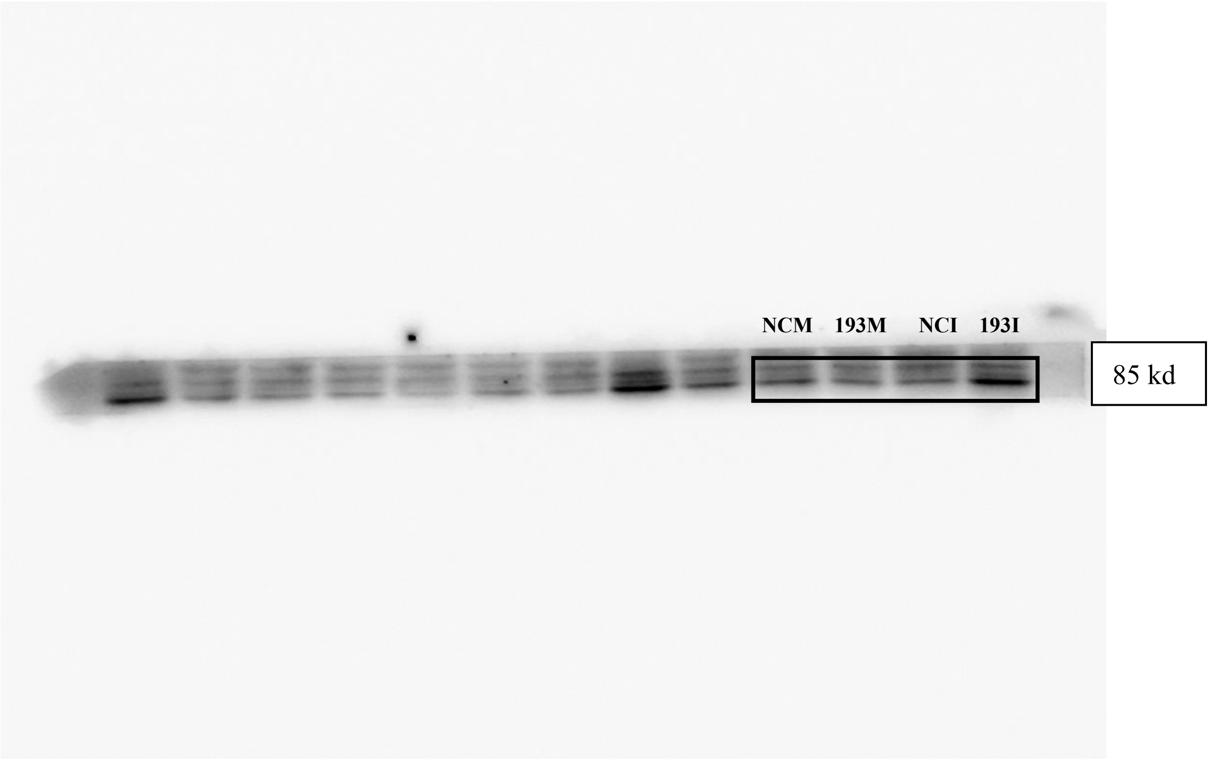

c-PARP

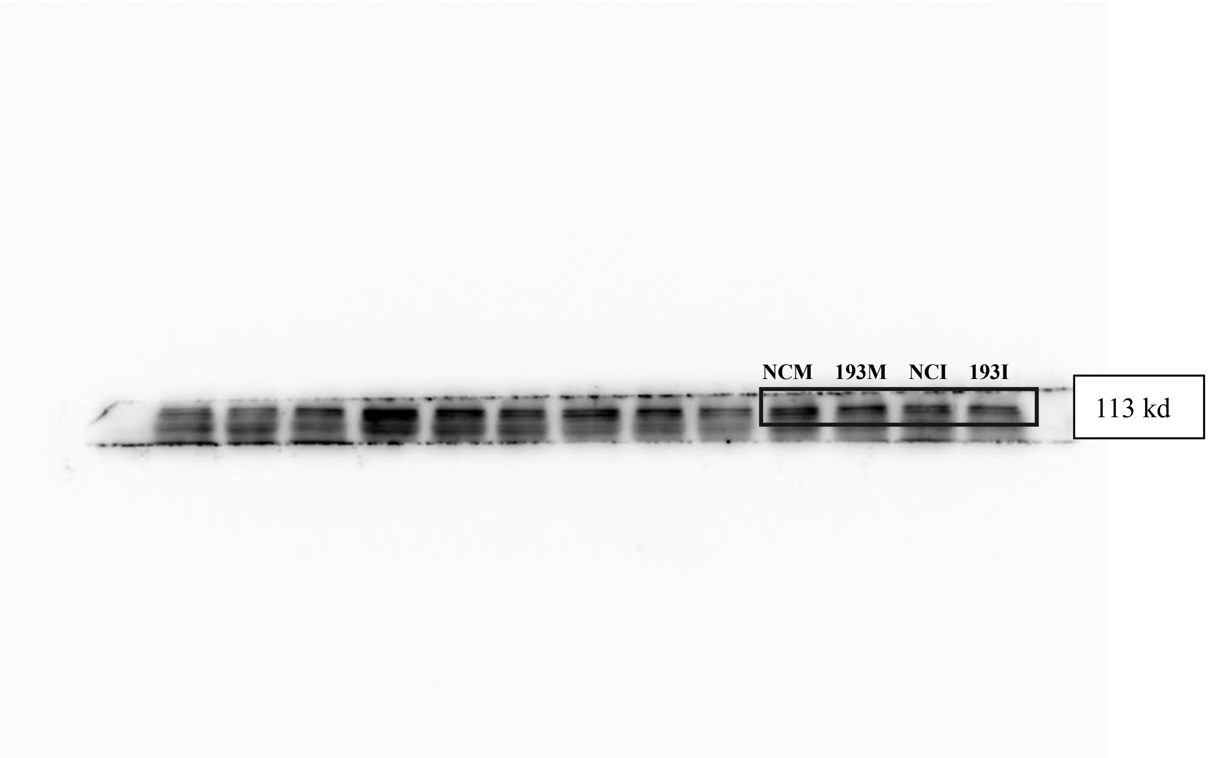

PARP

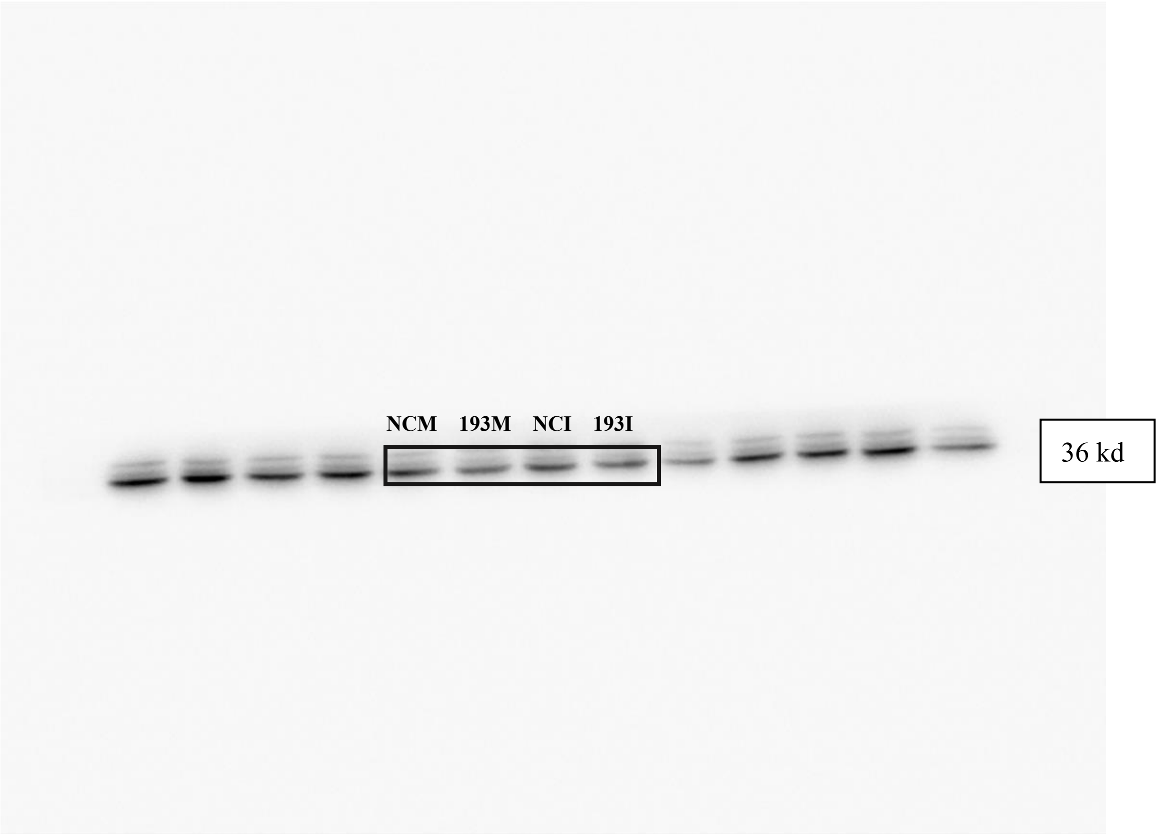

GAPDH

Images in Figure 8 D

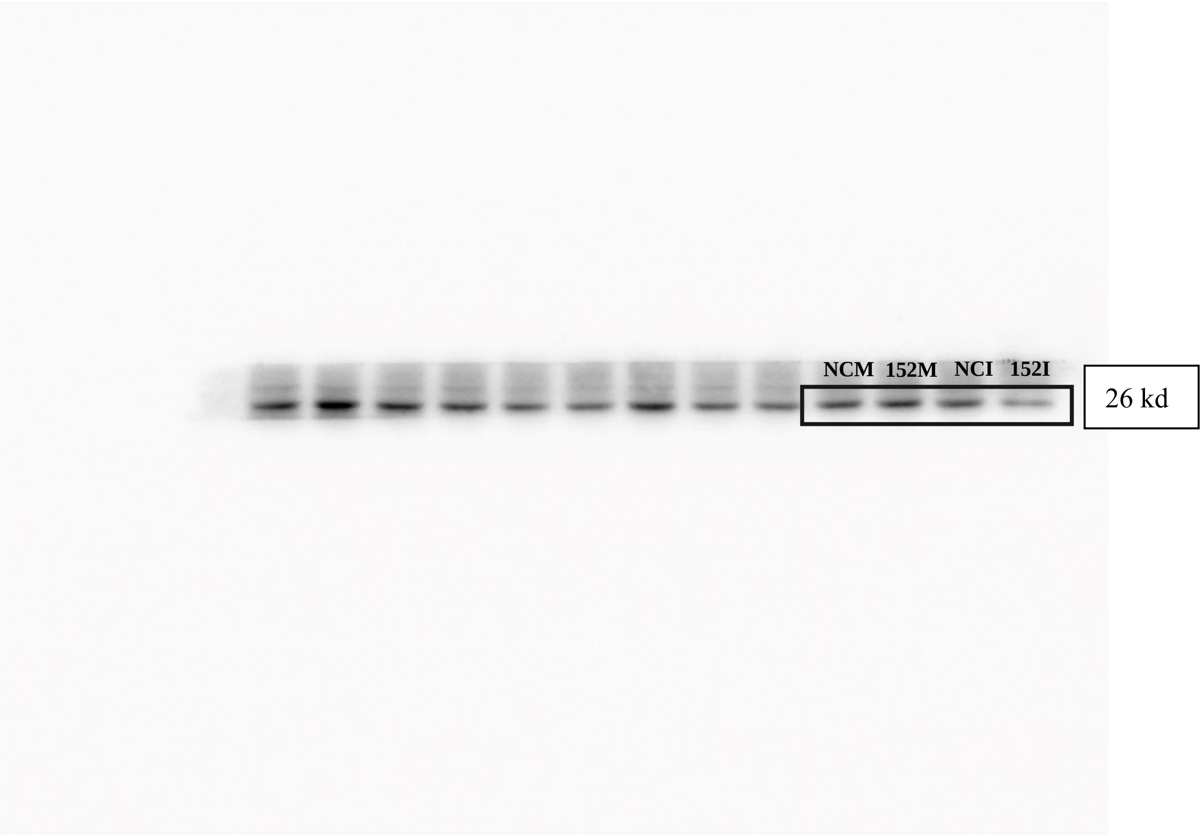

Bcl-2

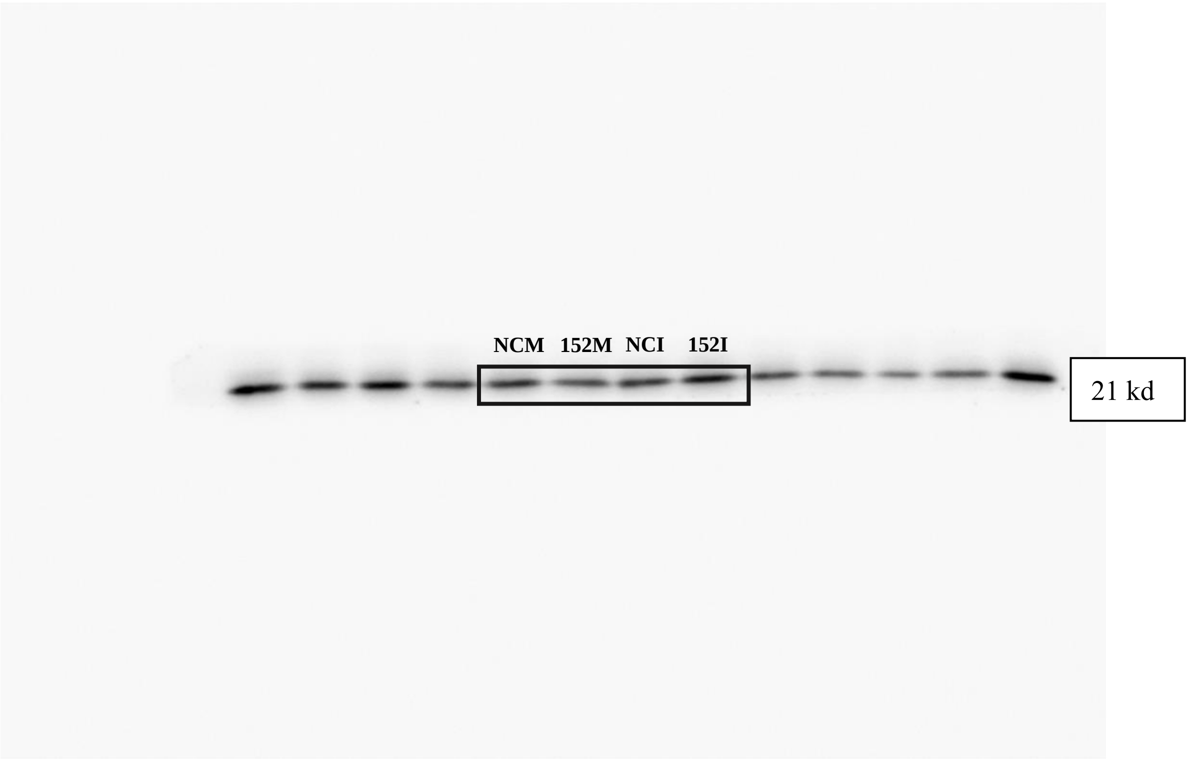

Bax

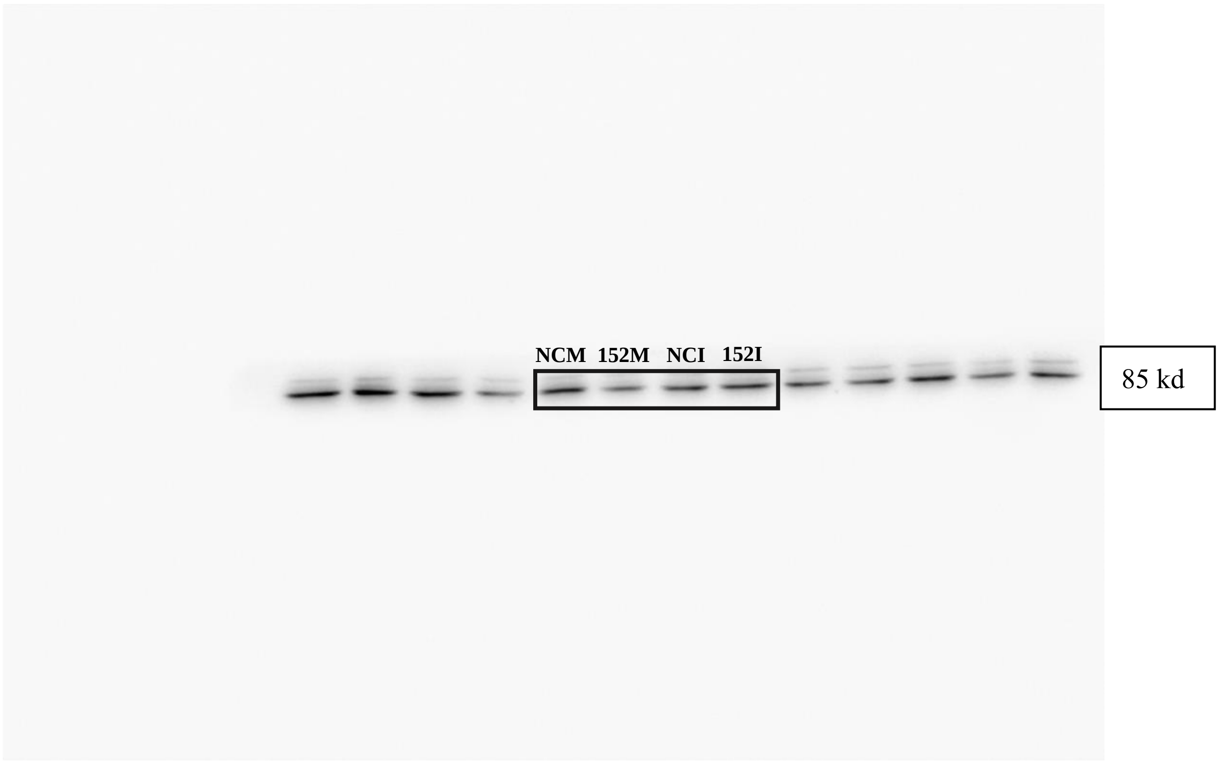

c-PARP

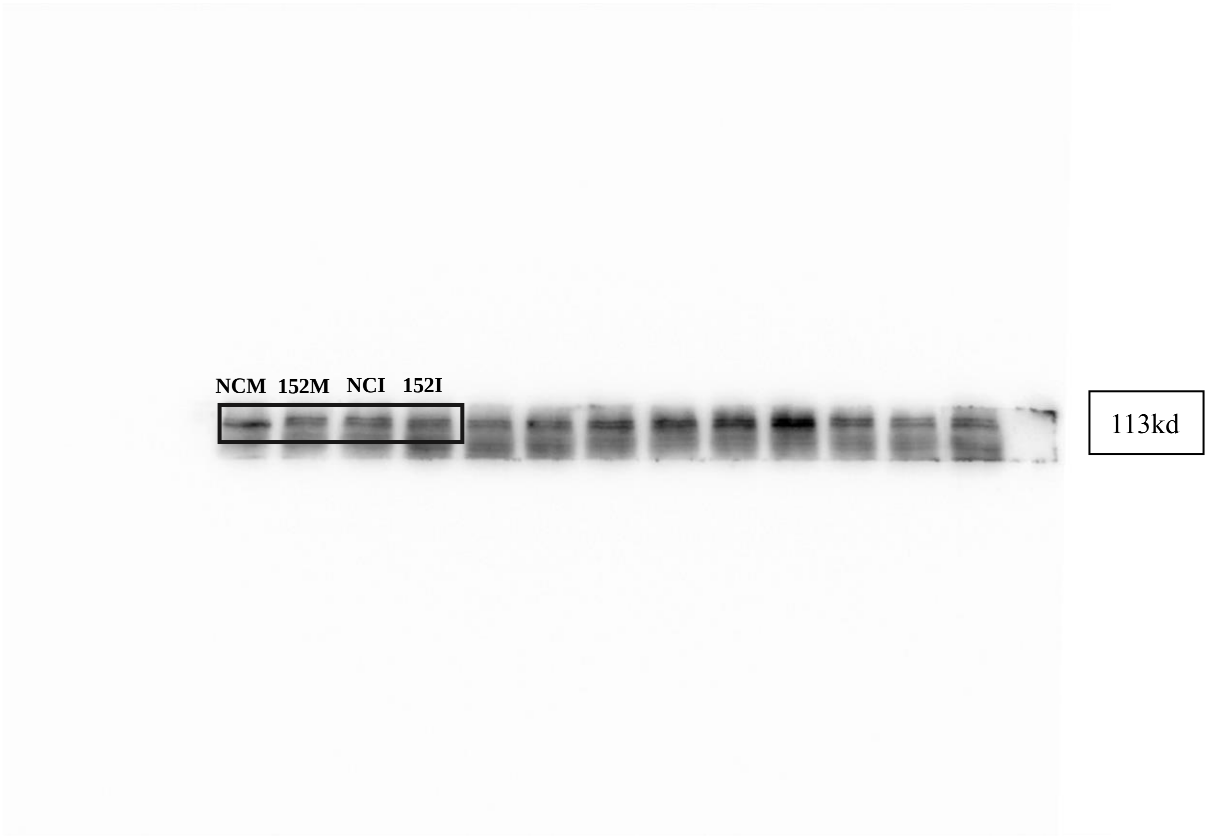

PARP

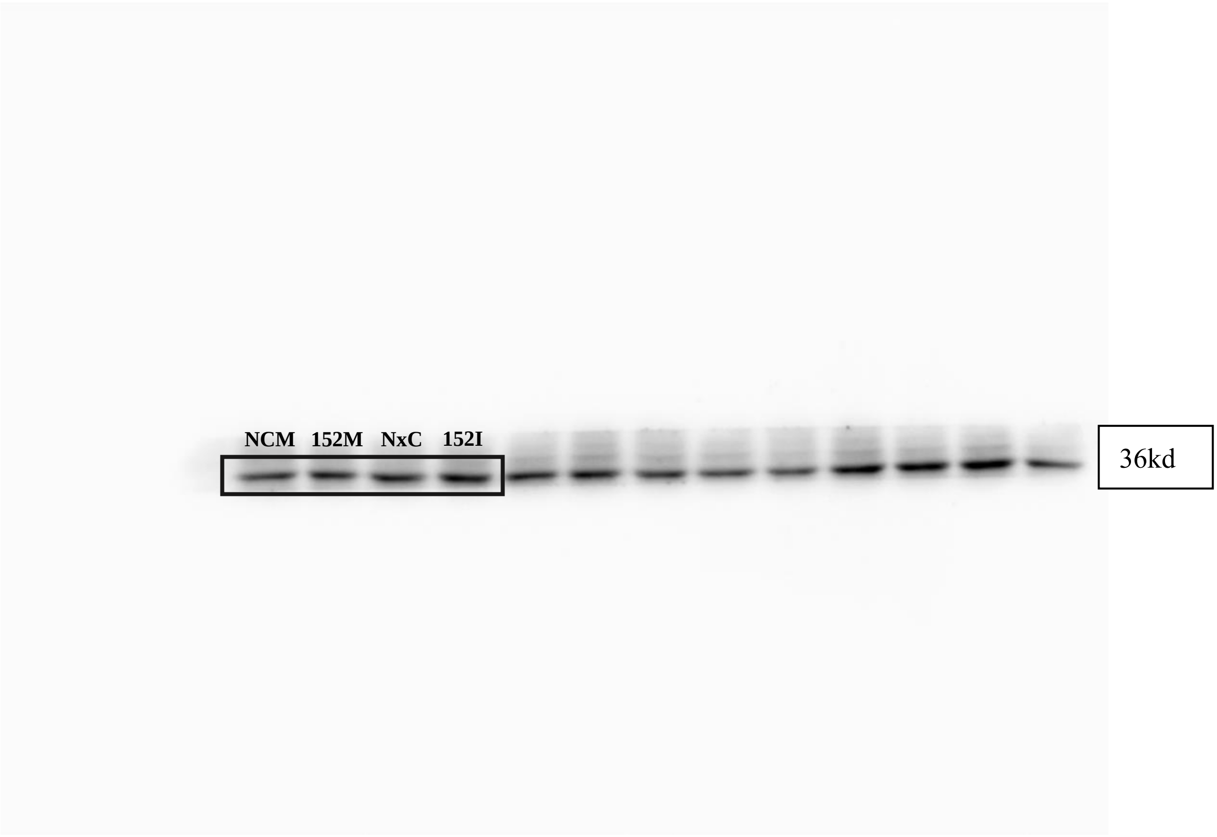

GAPDH

Images in Figure 8 G

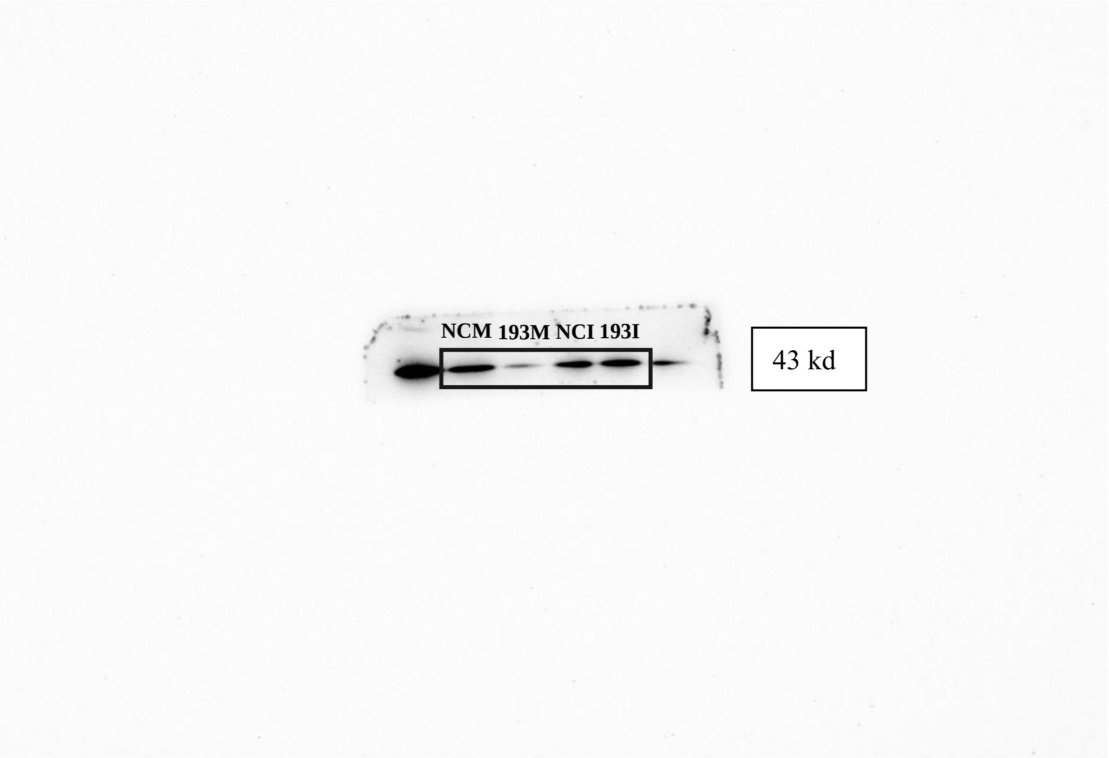

p-p38

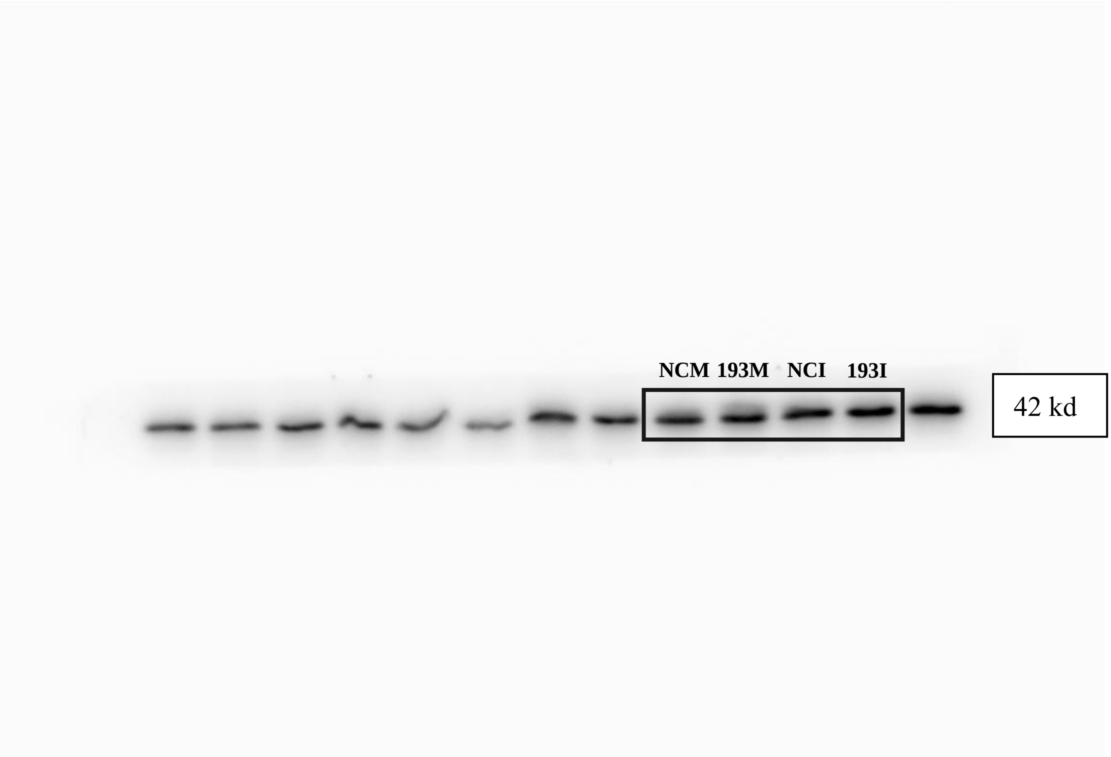

p38

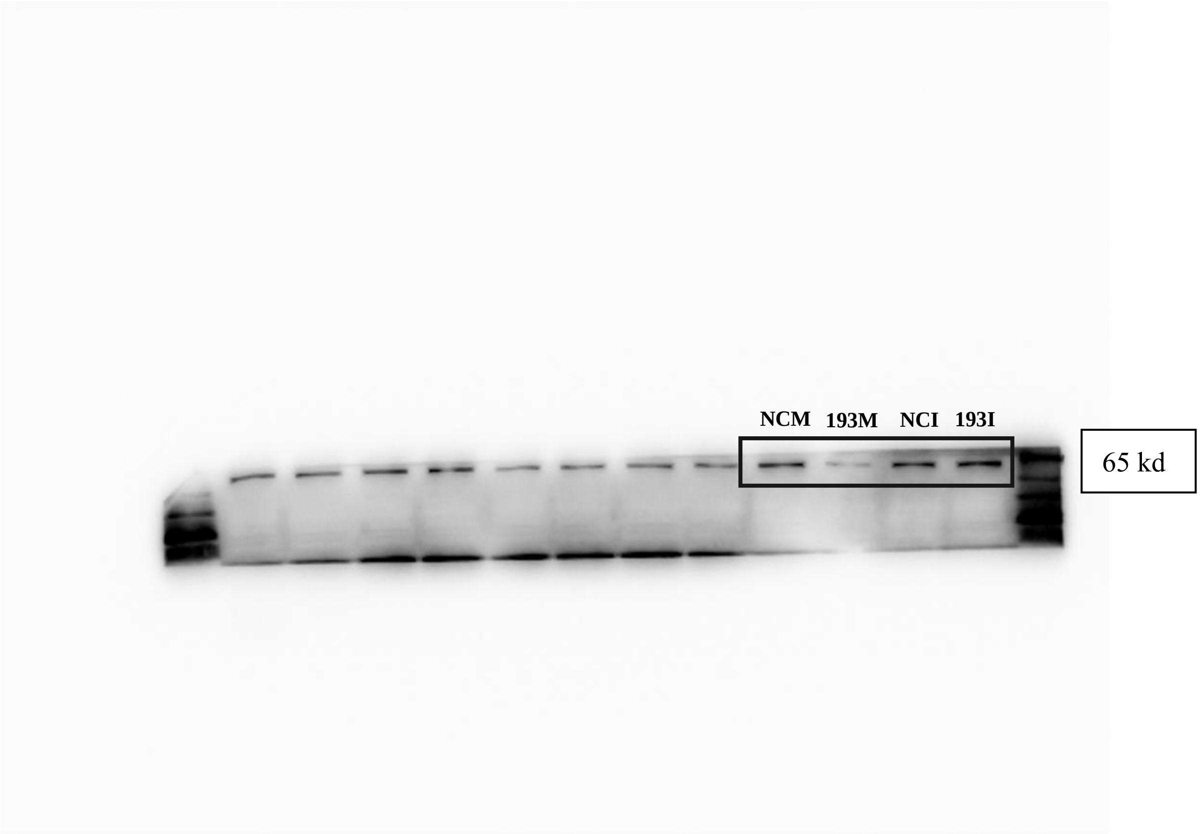

p-p65

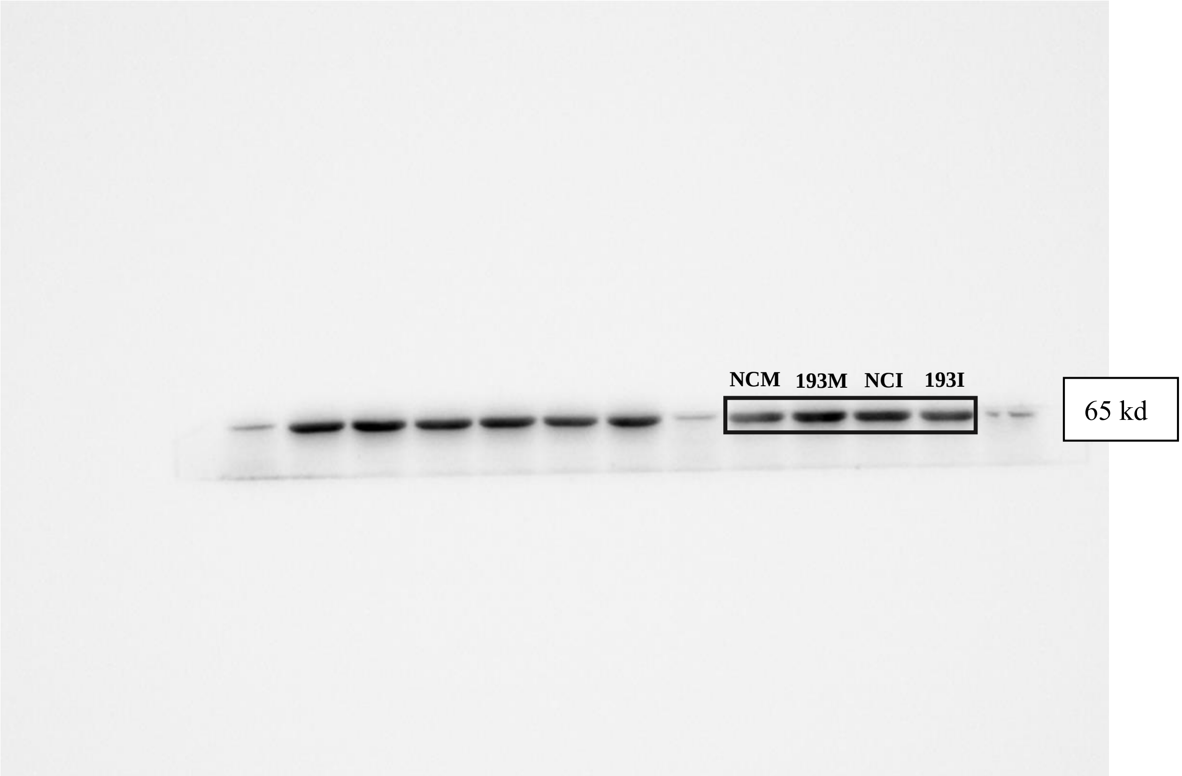

p65

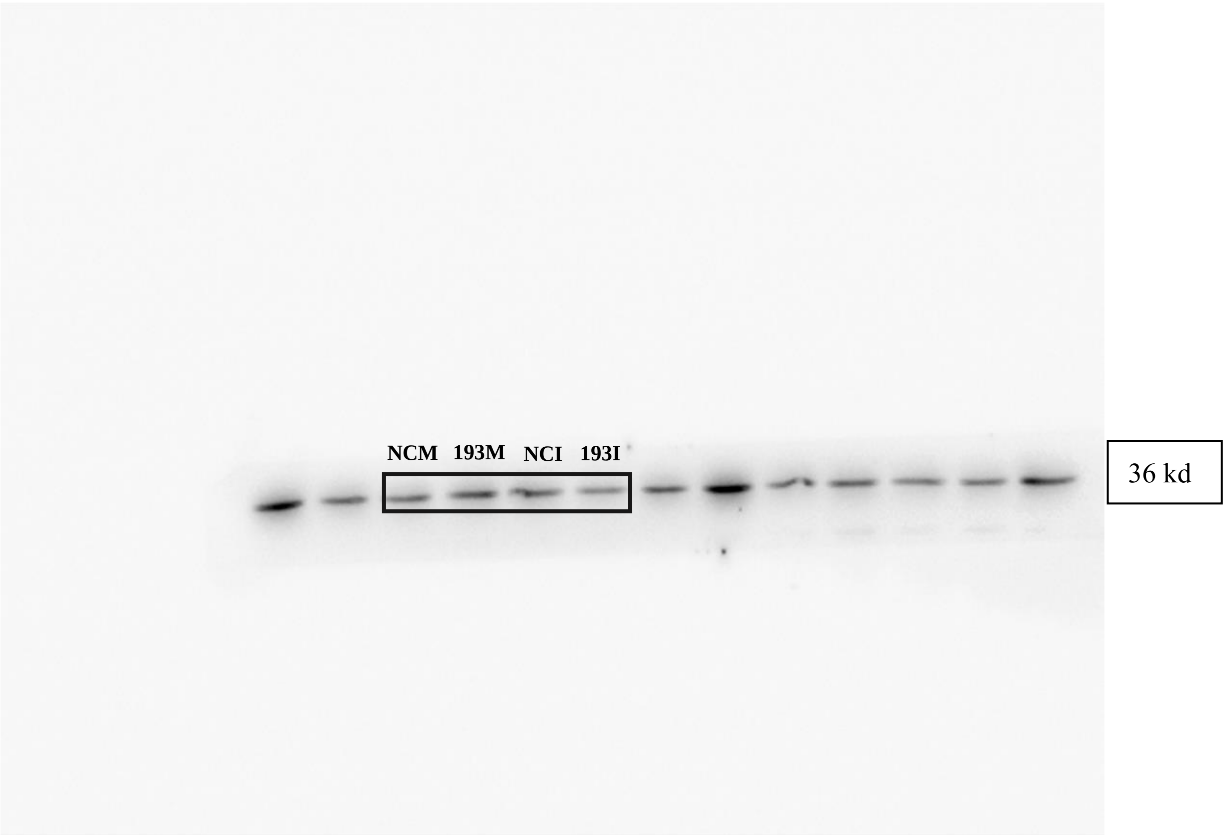

GAPDH

Images in Figure 8 J

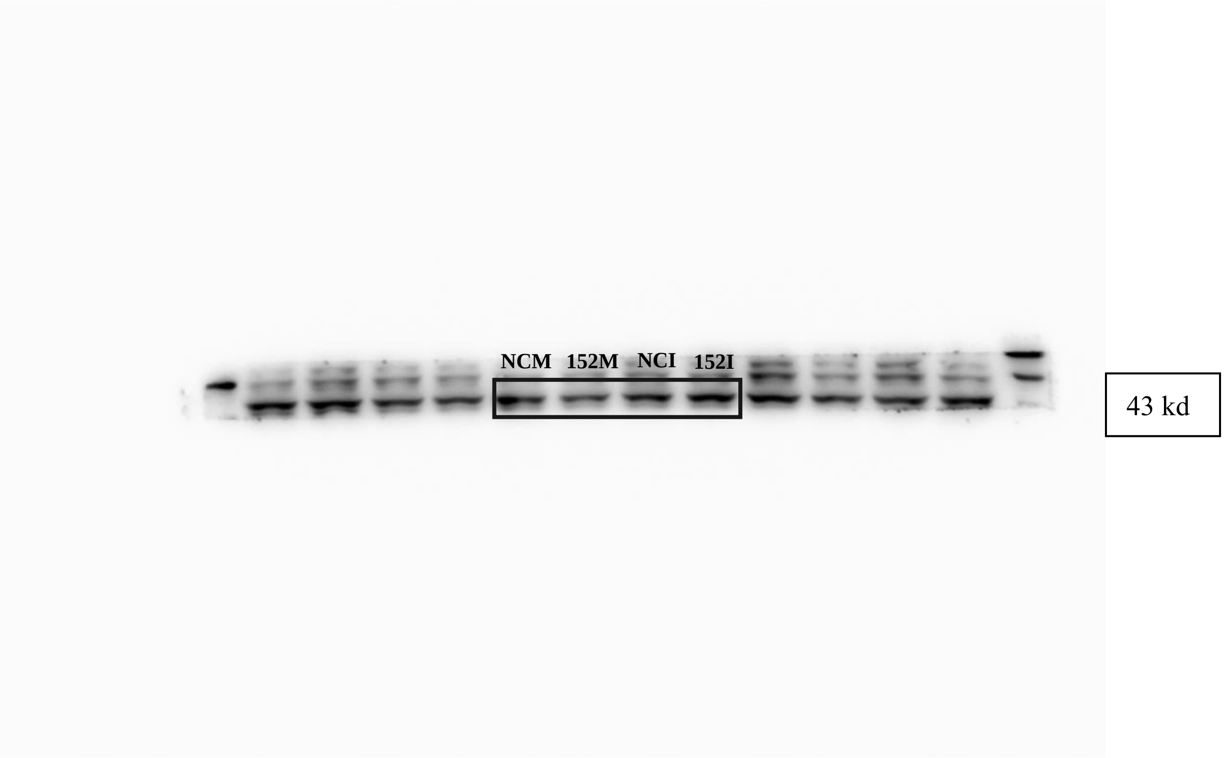

p-p38

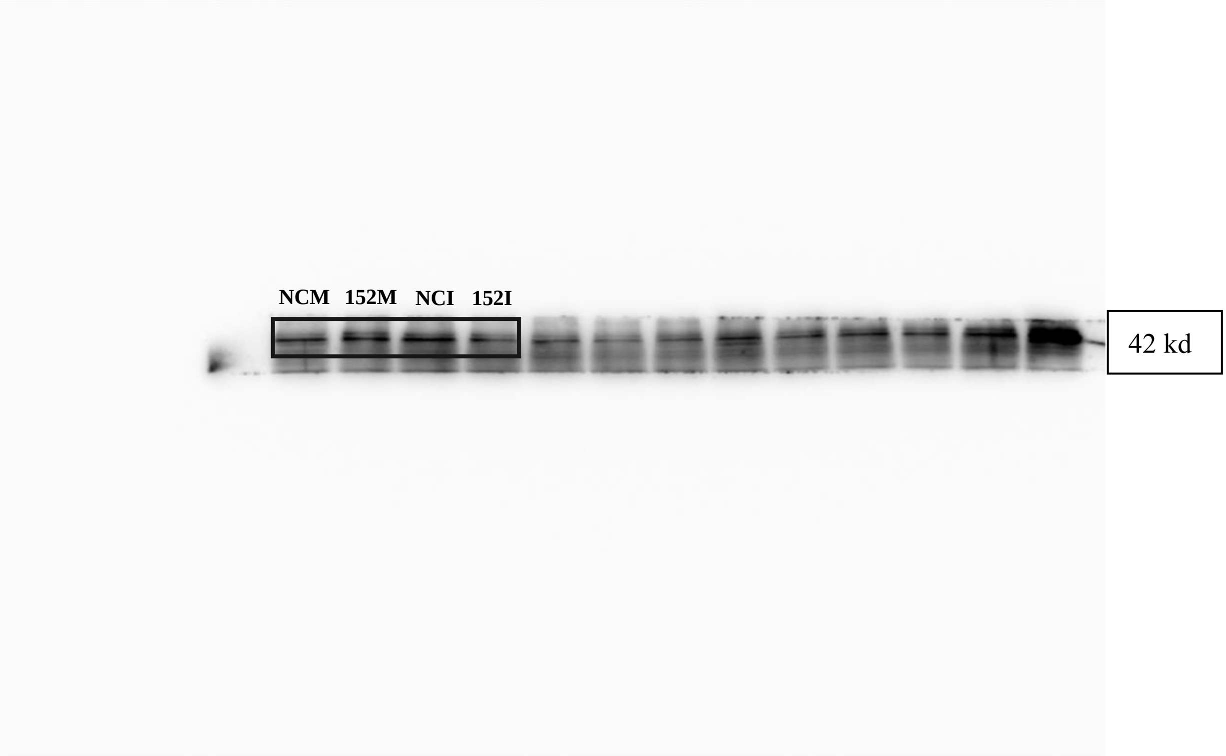

p38

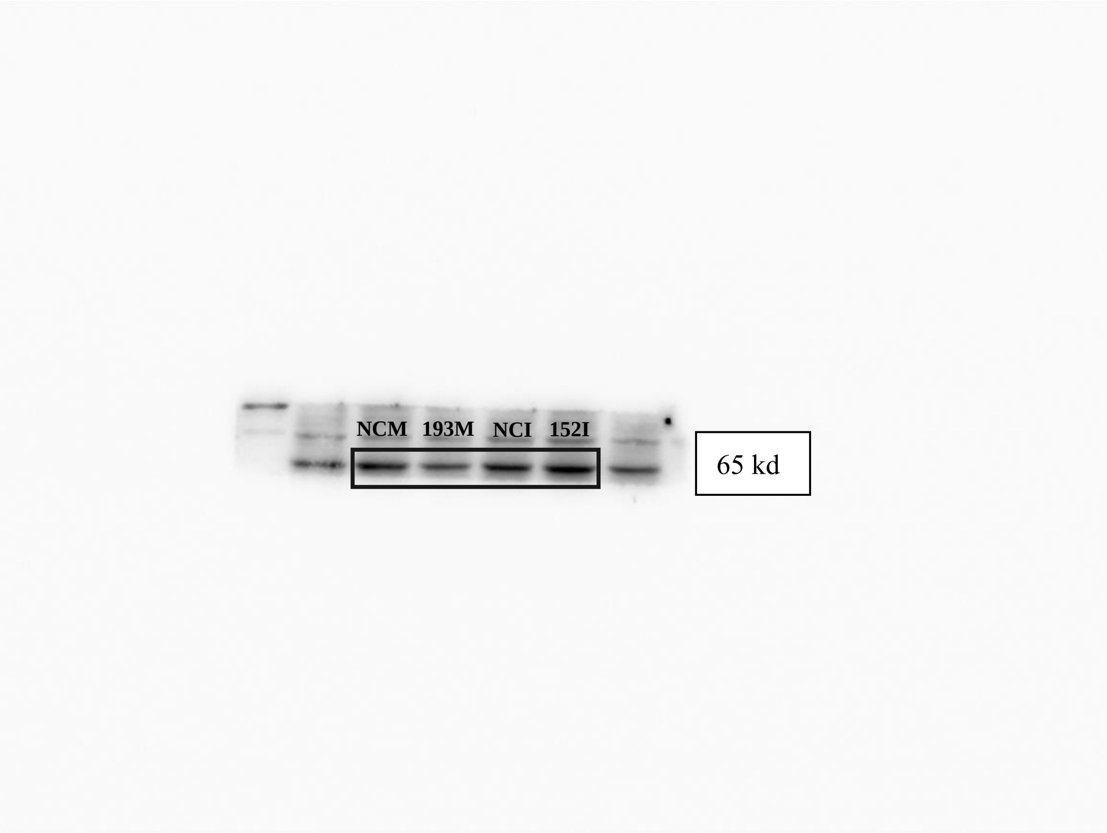

p-p65

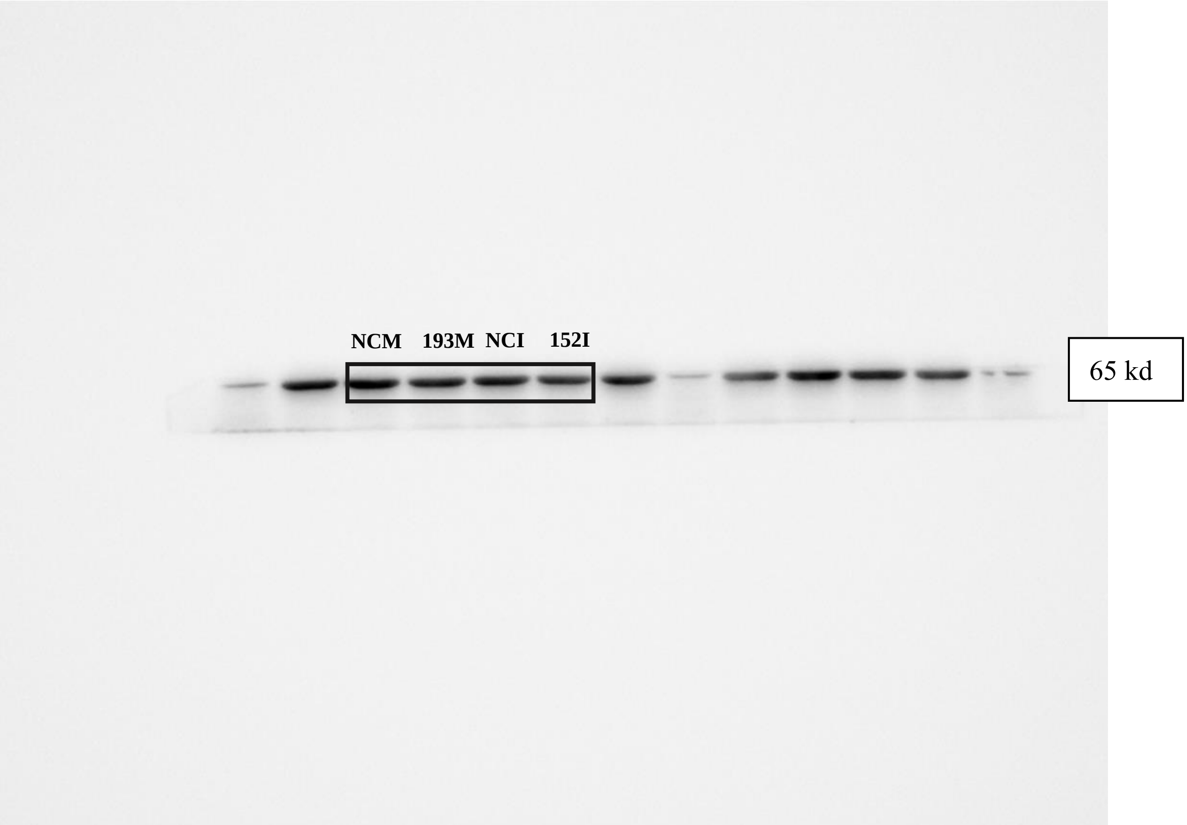

p65

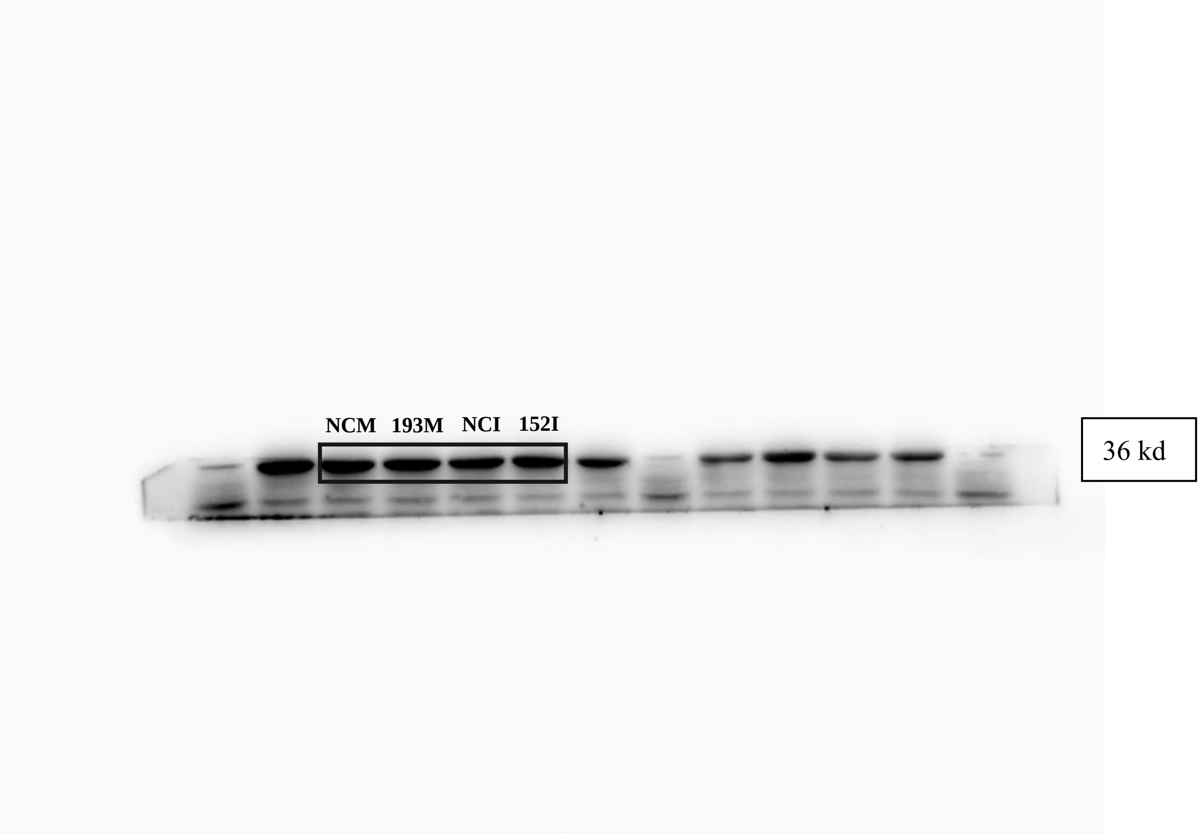

GAPDH

Images in Figure 9 K

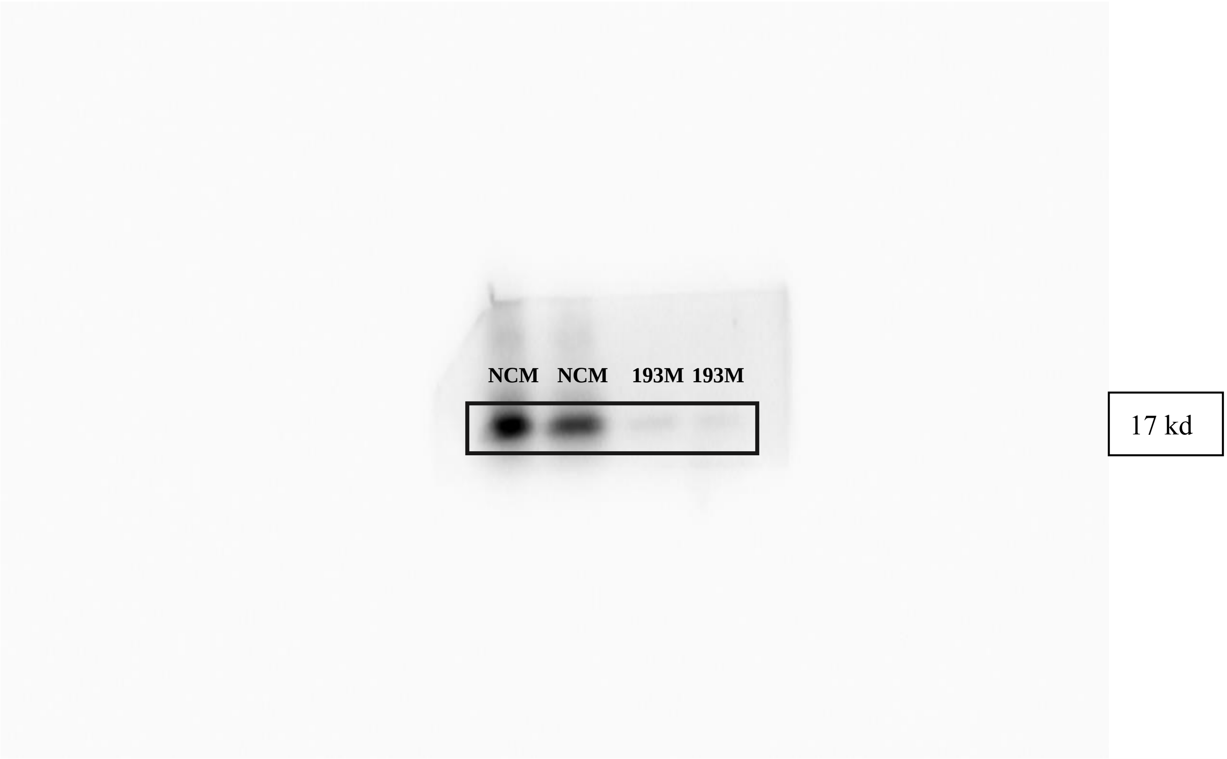

CaM

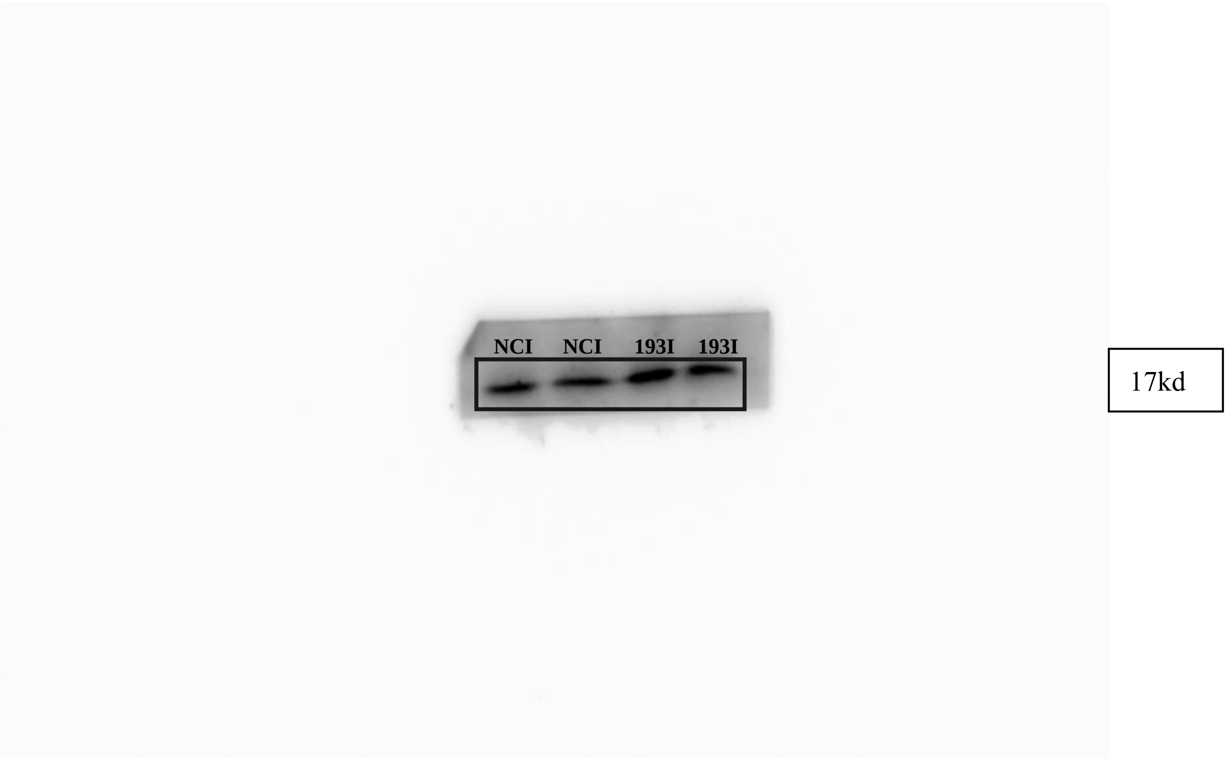

CaM

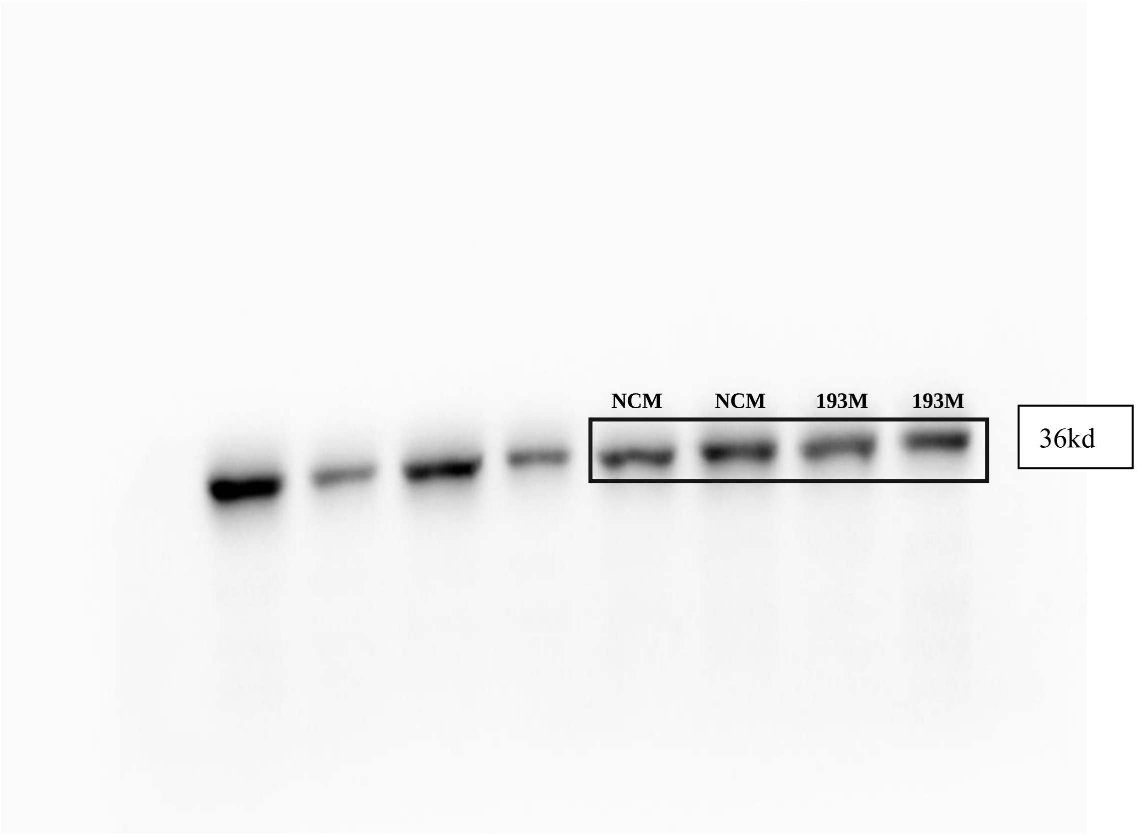

GAPDH

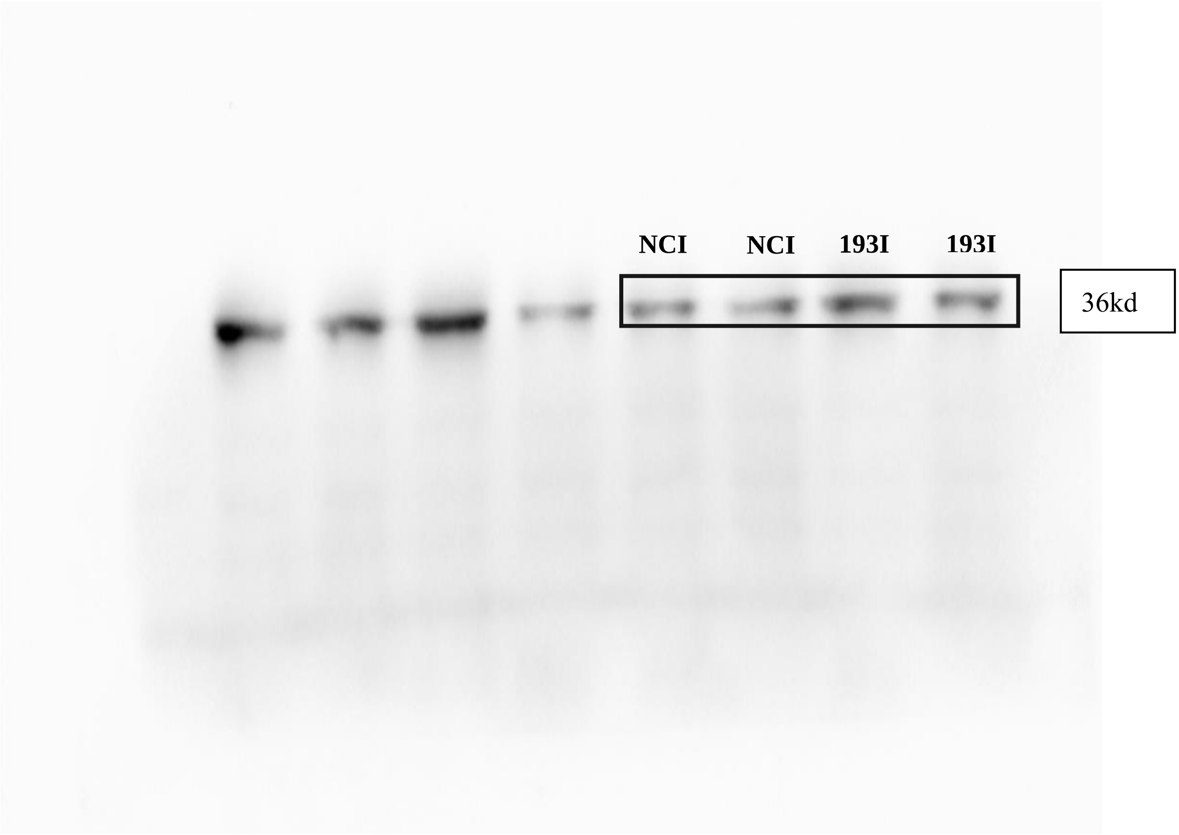

GAPDH

Images in Figure 9 L

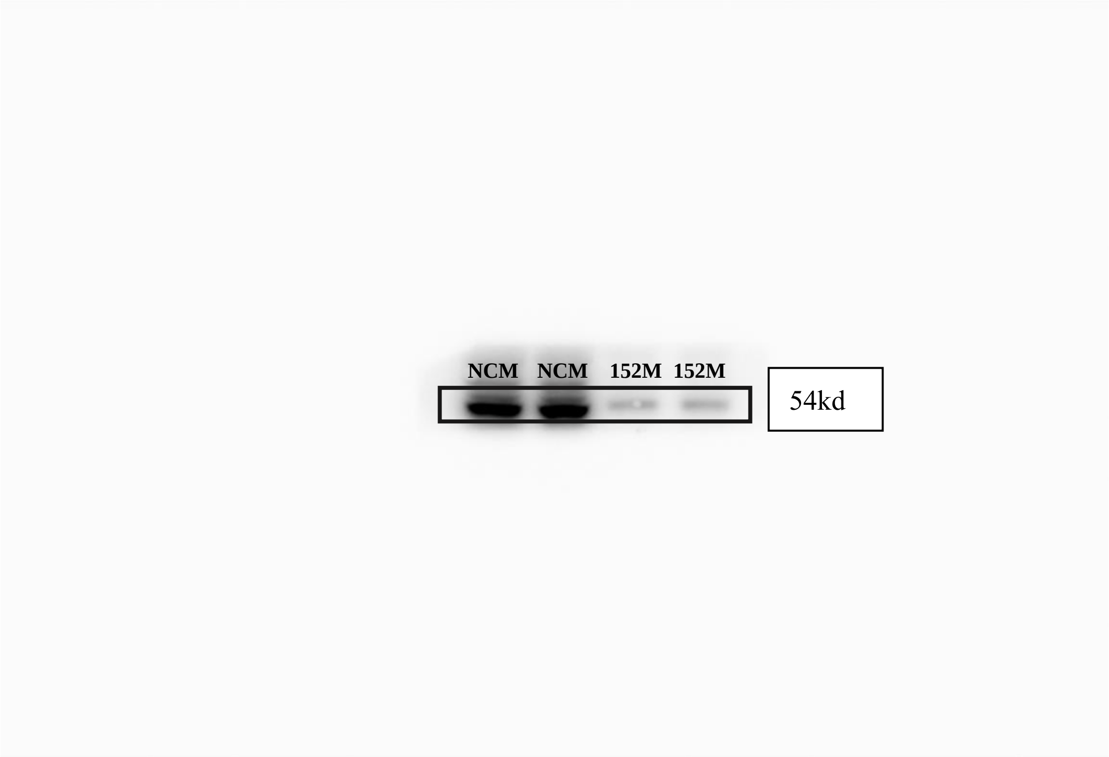

CaMKIIα

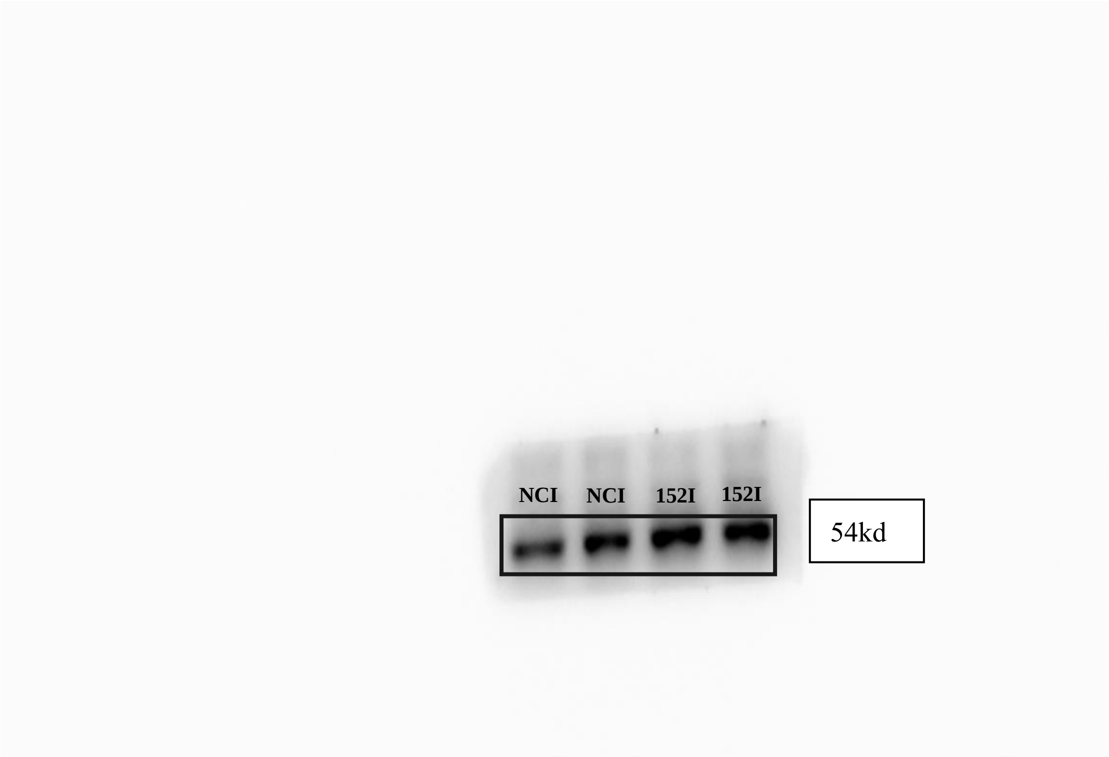

CaMKIIα

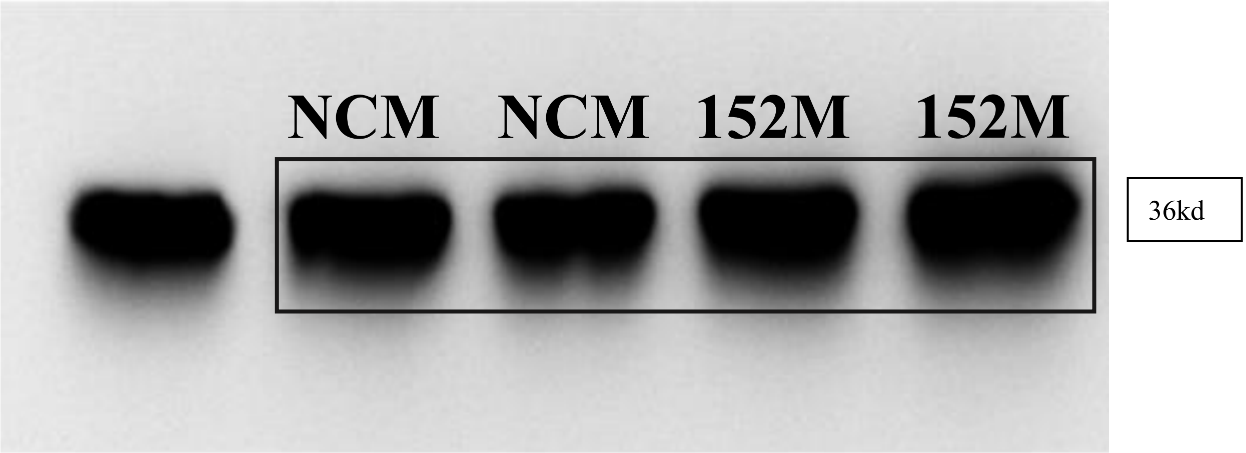

GAPDH

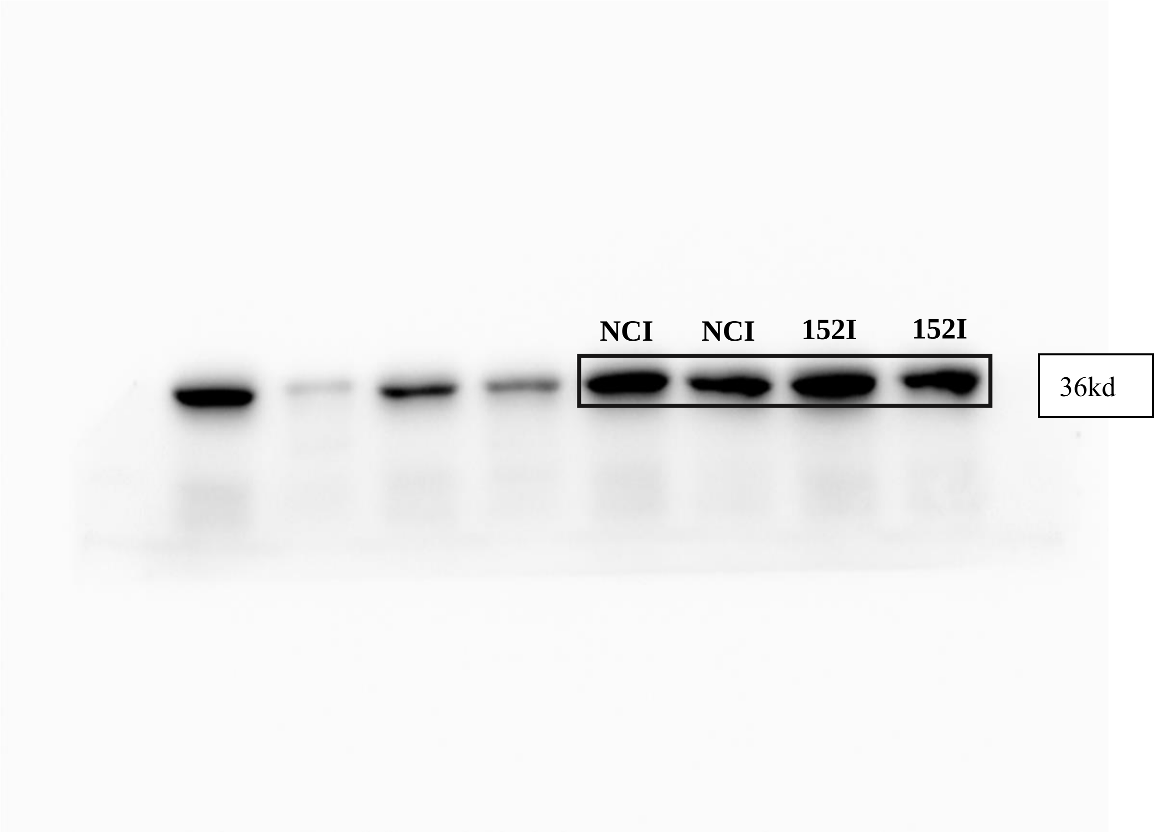

GAPDH

Images in Figure 11 A

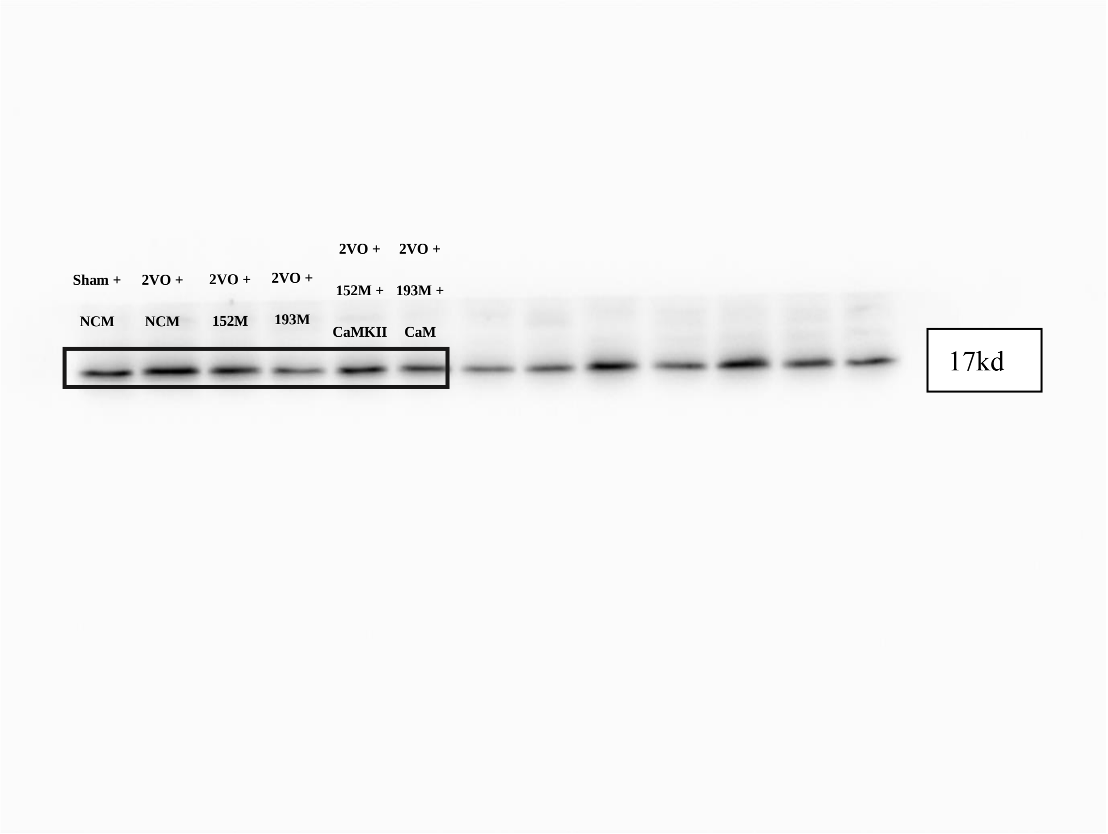

CaM

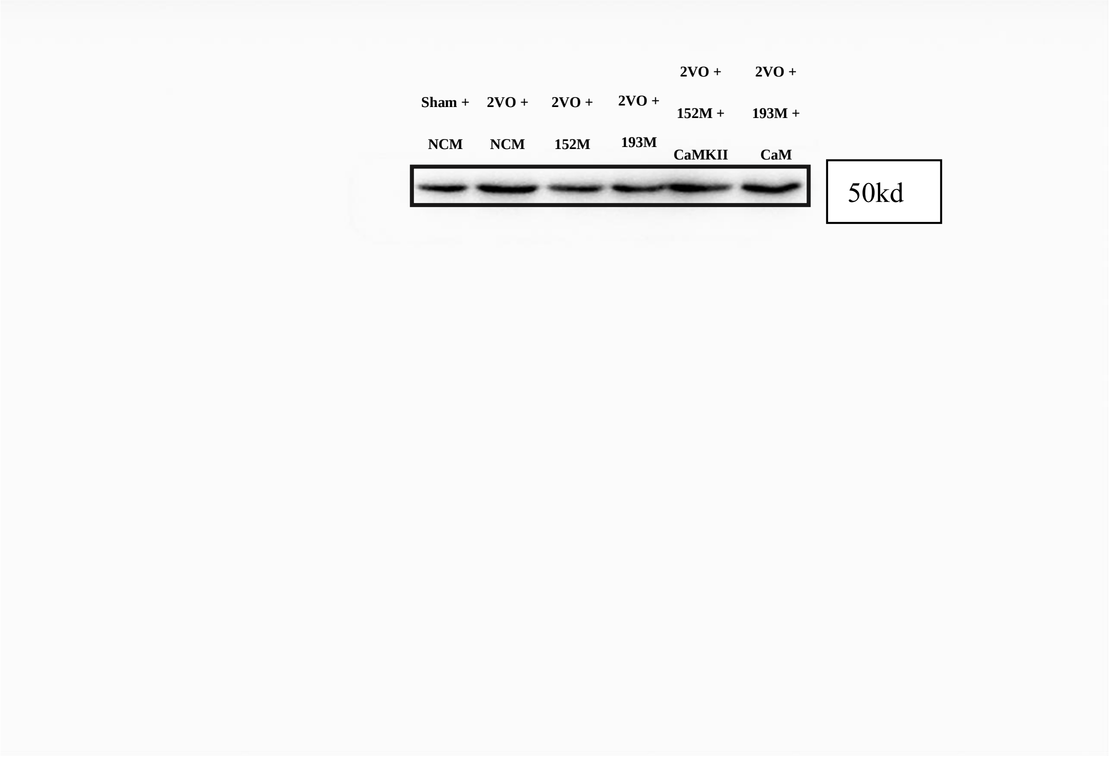

ox-CaMKIIα

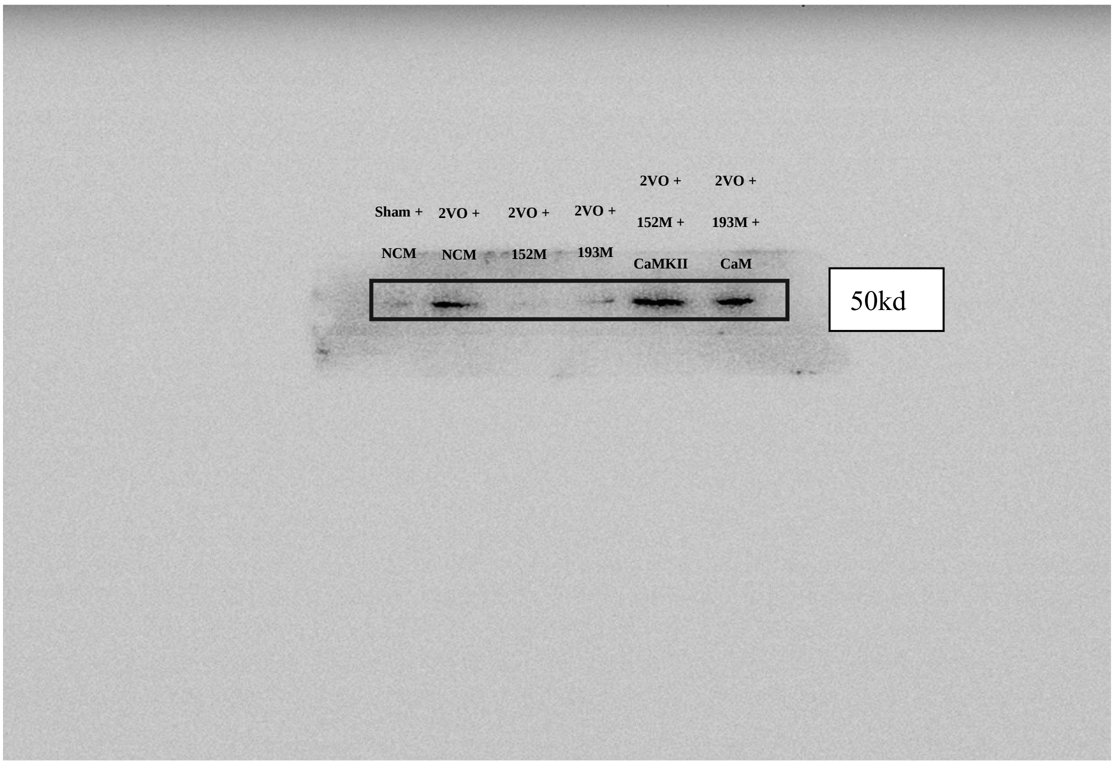

p-CaMKIIα

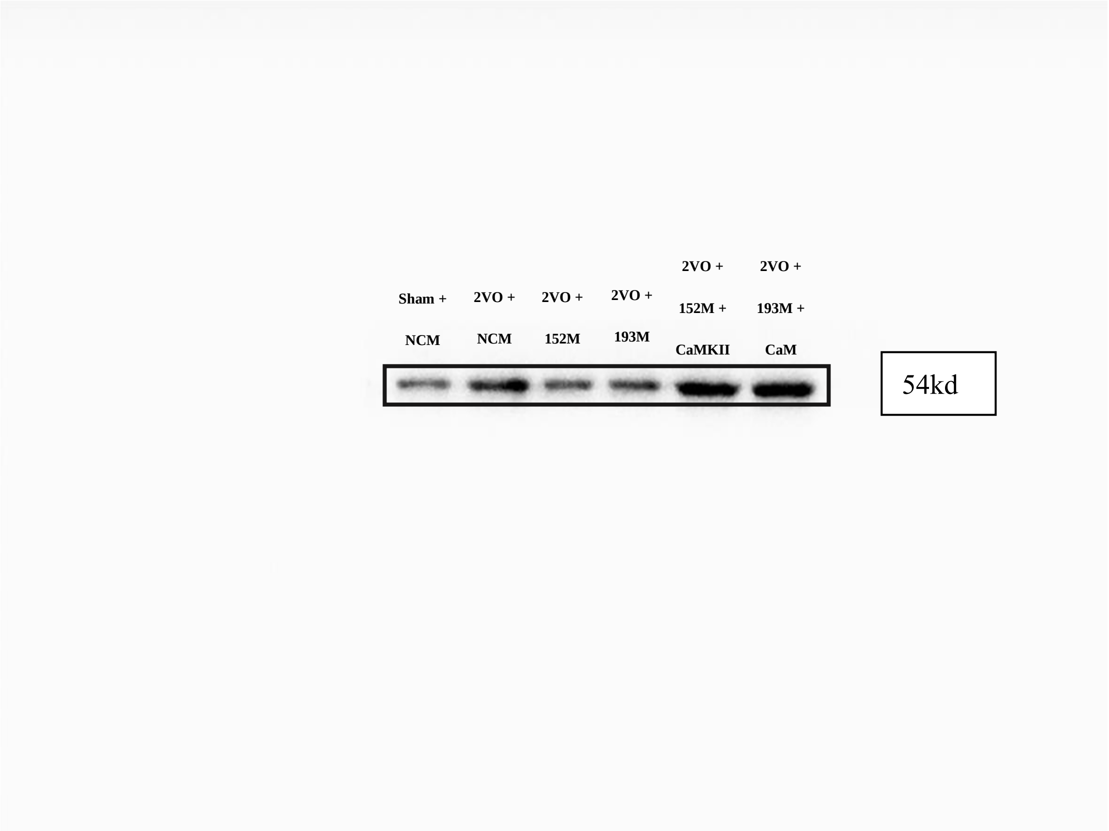

CaMKIIα

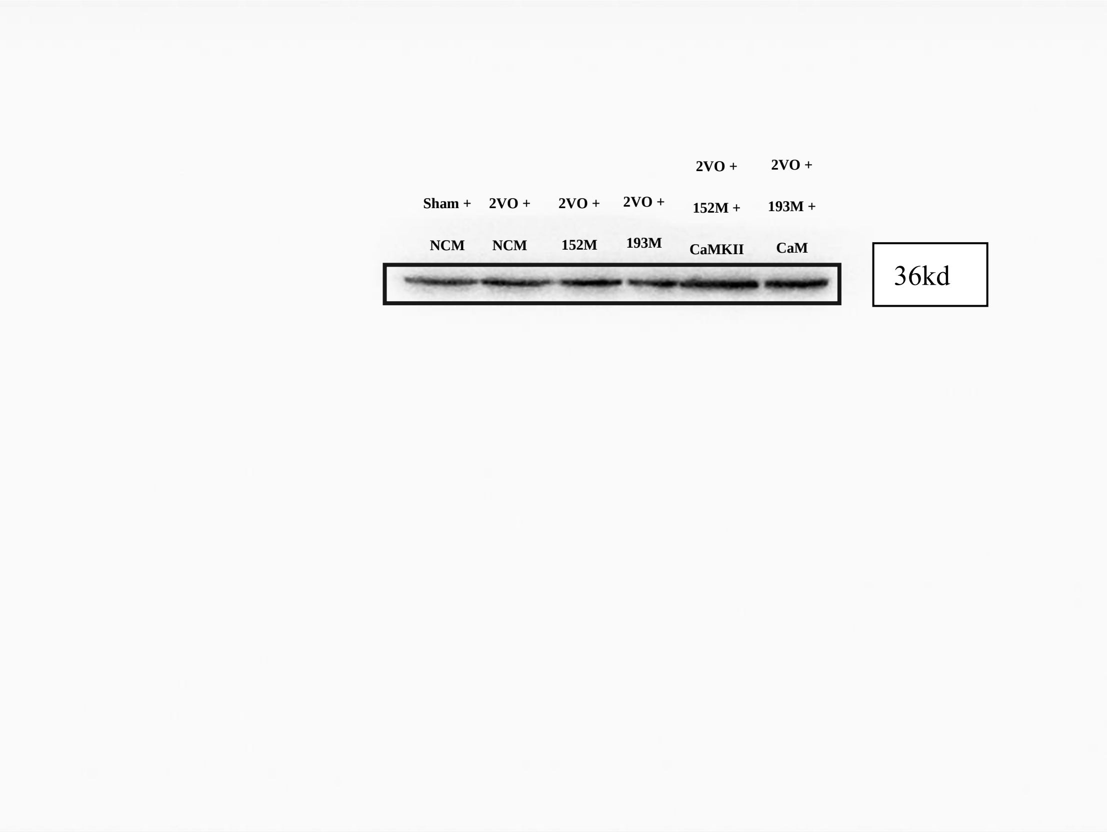
Sham + NCM
2VO + NCM
2VO + 152M
2VO + 193M
2VO + 152M + CaMKII
2VO + 193M + CaM
36kd

GAPDH

Images in Figure 11 C

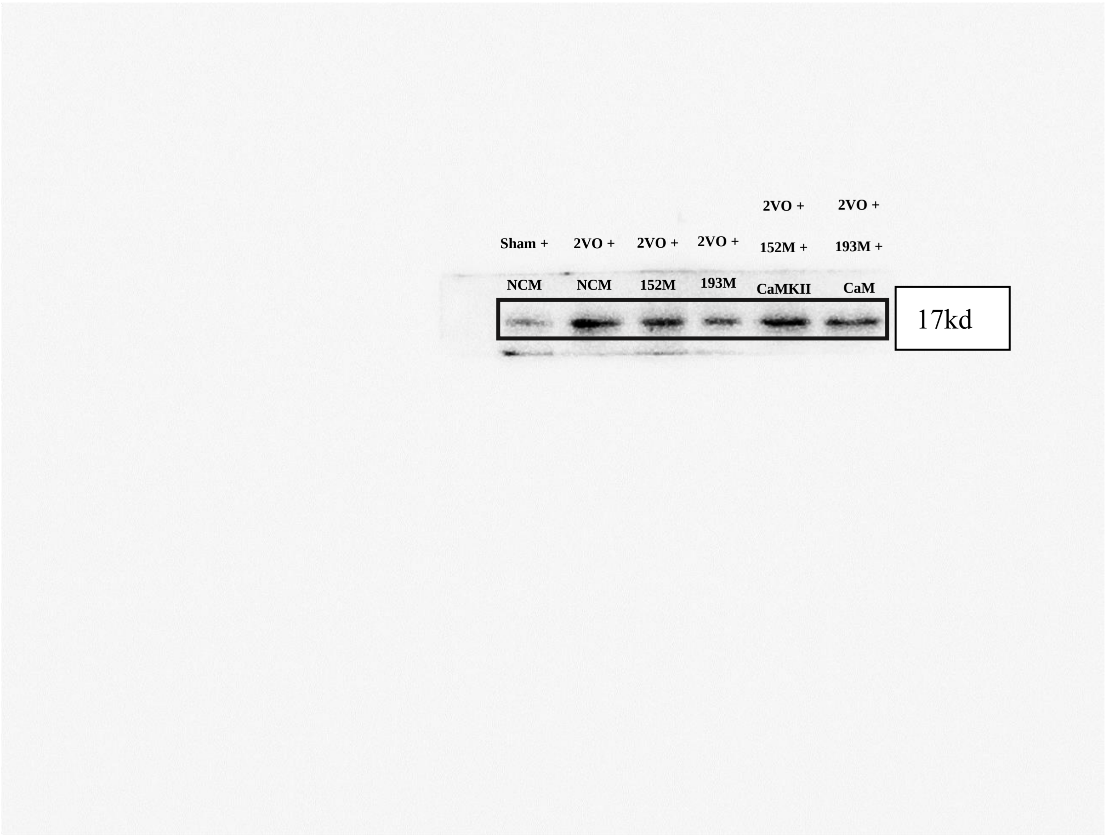

CaM

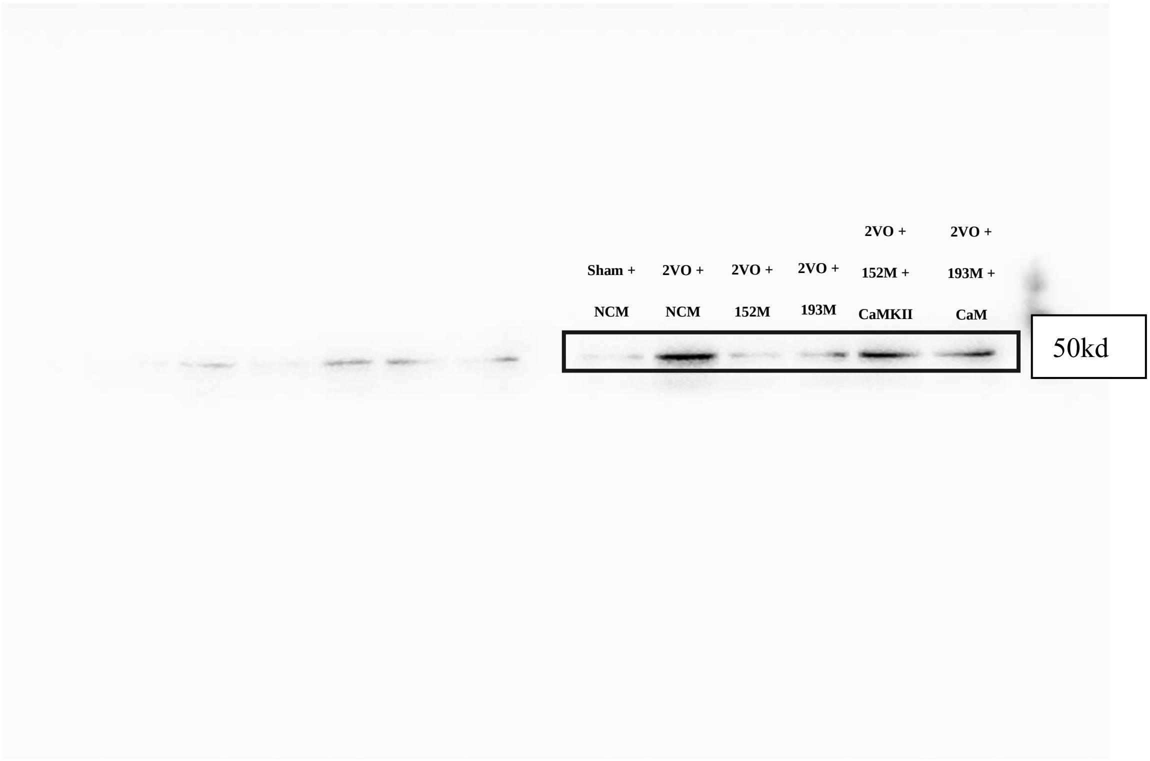

ox-CaMKIIα

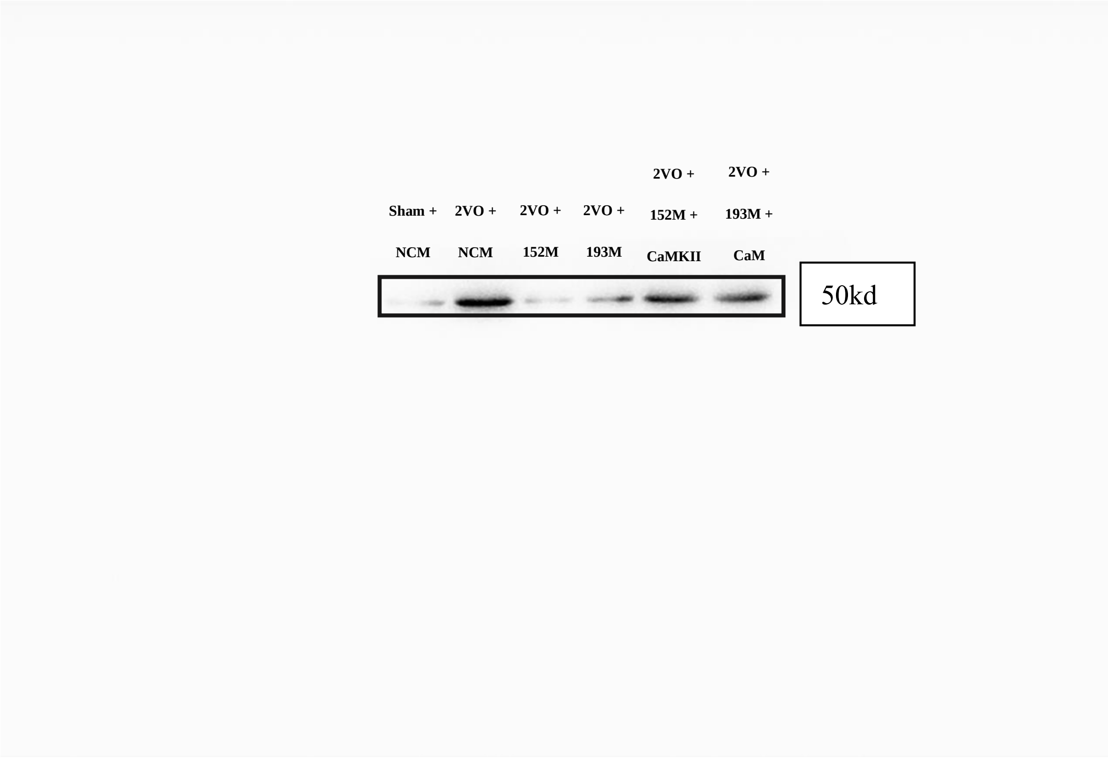

p-CaMKIIα

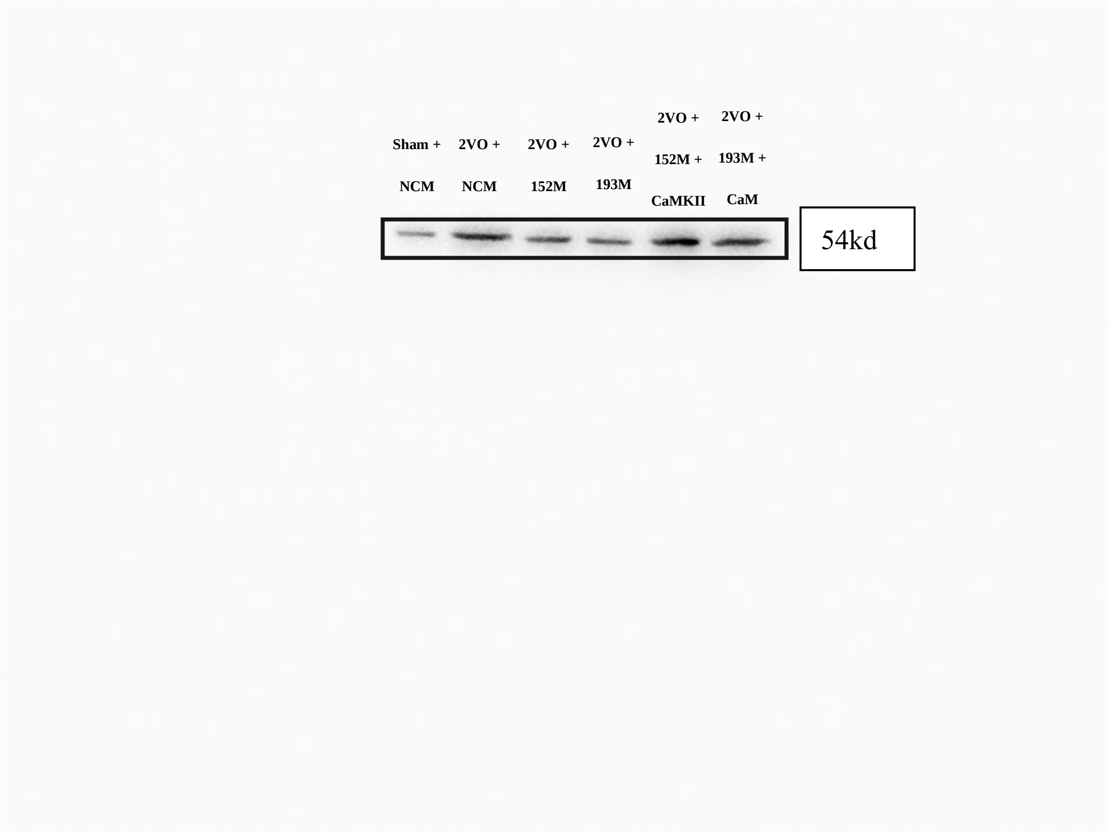

CaMKIIα

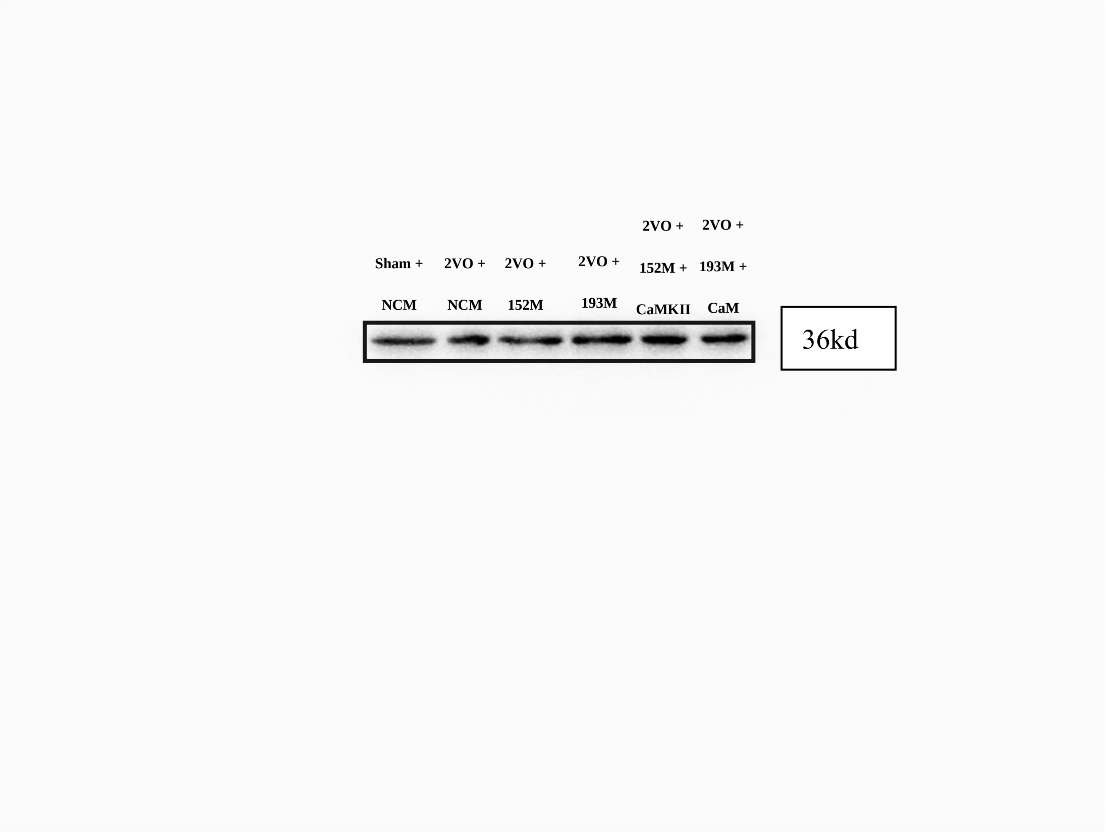
2VO +
2VO +
Sham +
2VO +
2VO +
2VO +
152M +
193M +
NCM
NCM
152M
193M
CaMKII
CaM
36kd

GAPDH

Images in Figure 11 E

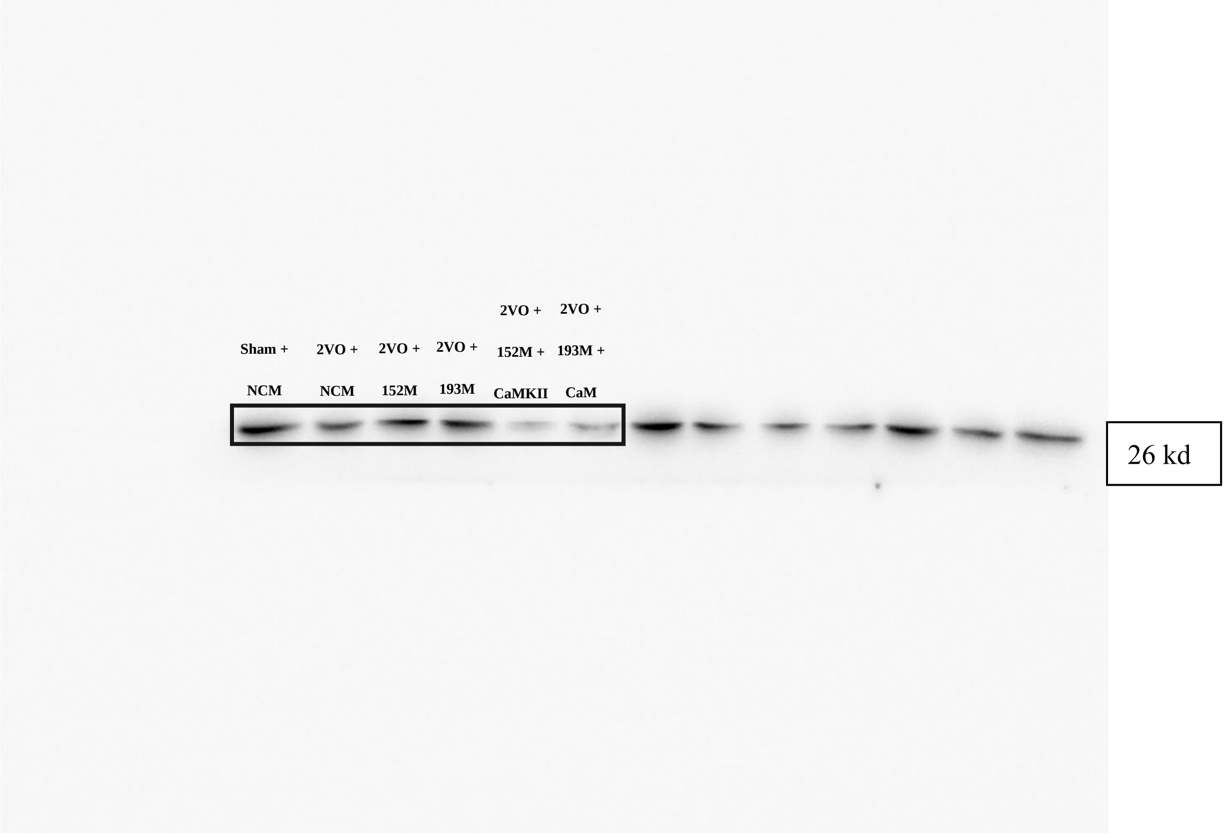

Bcl-2

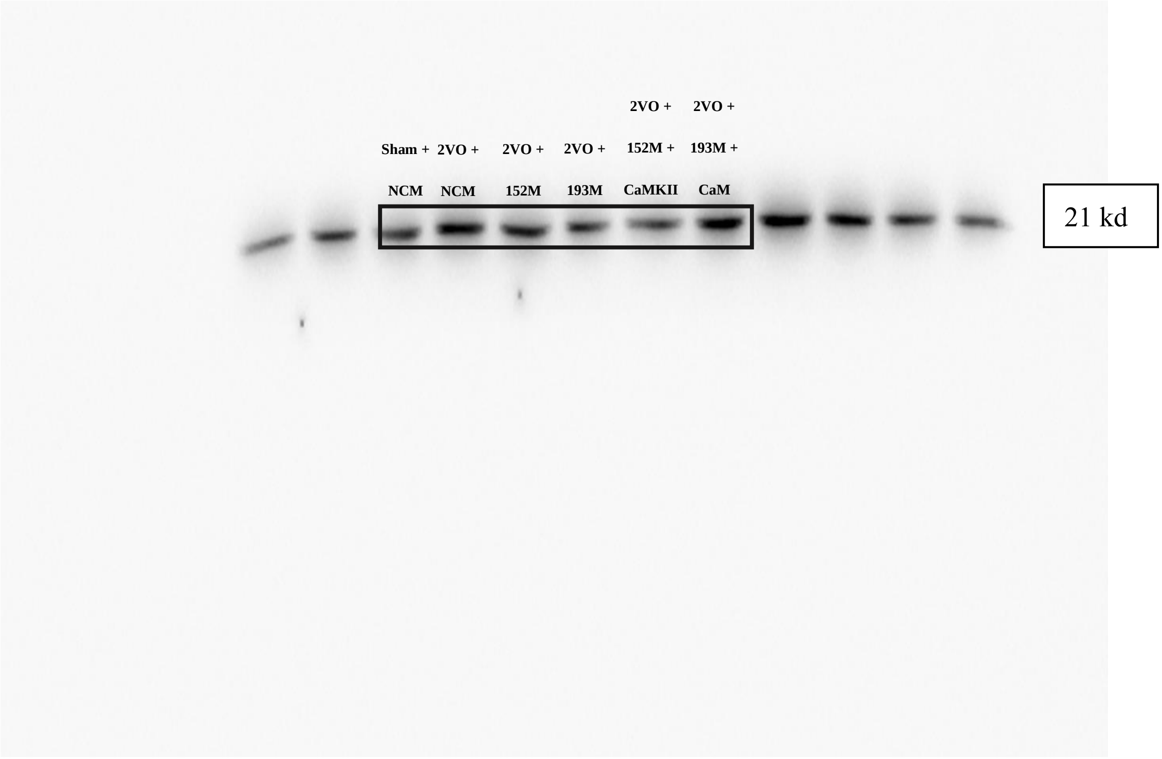

Bax

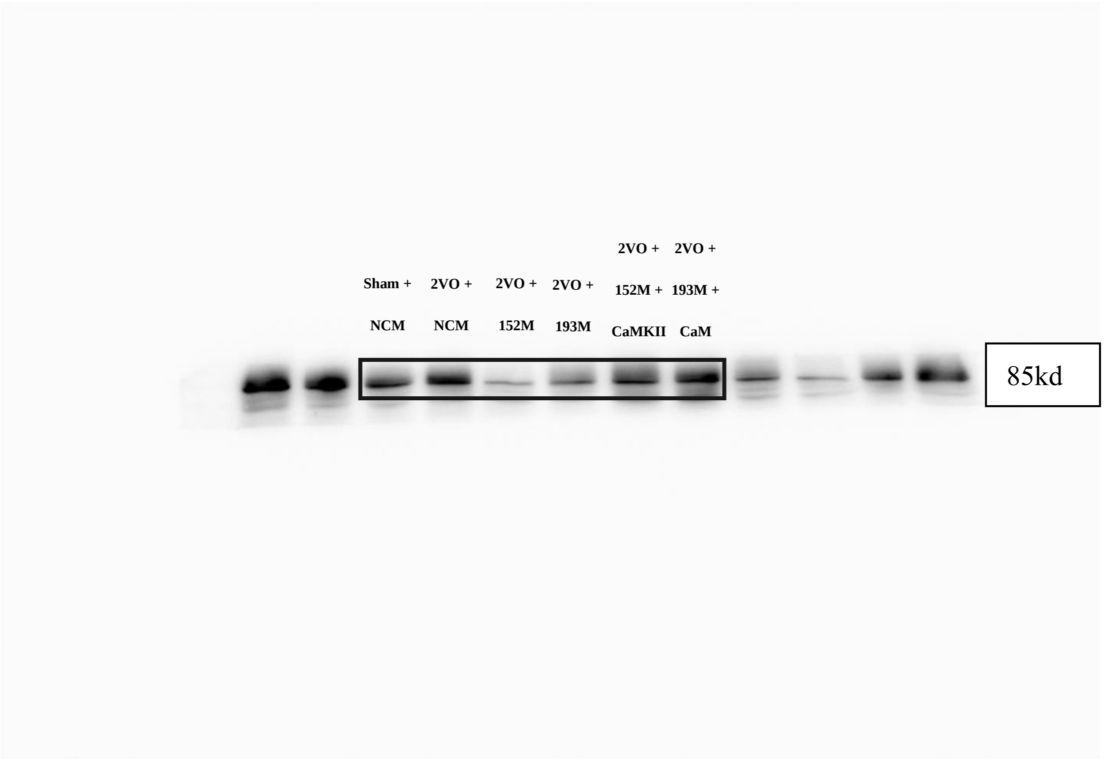
Sham + NCM
2VO + NCM
2VO + 152M
2VO + 193M
2VO + 152M + CaMKII
2VO + 193M + CaM
85kd

c-PARP

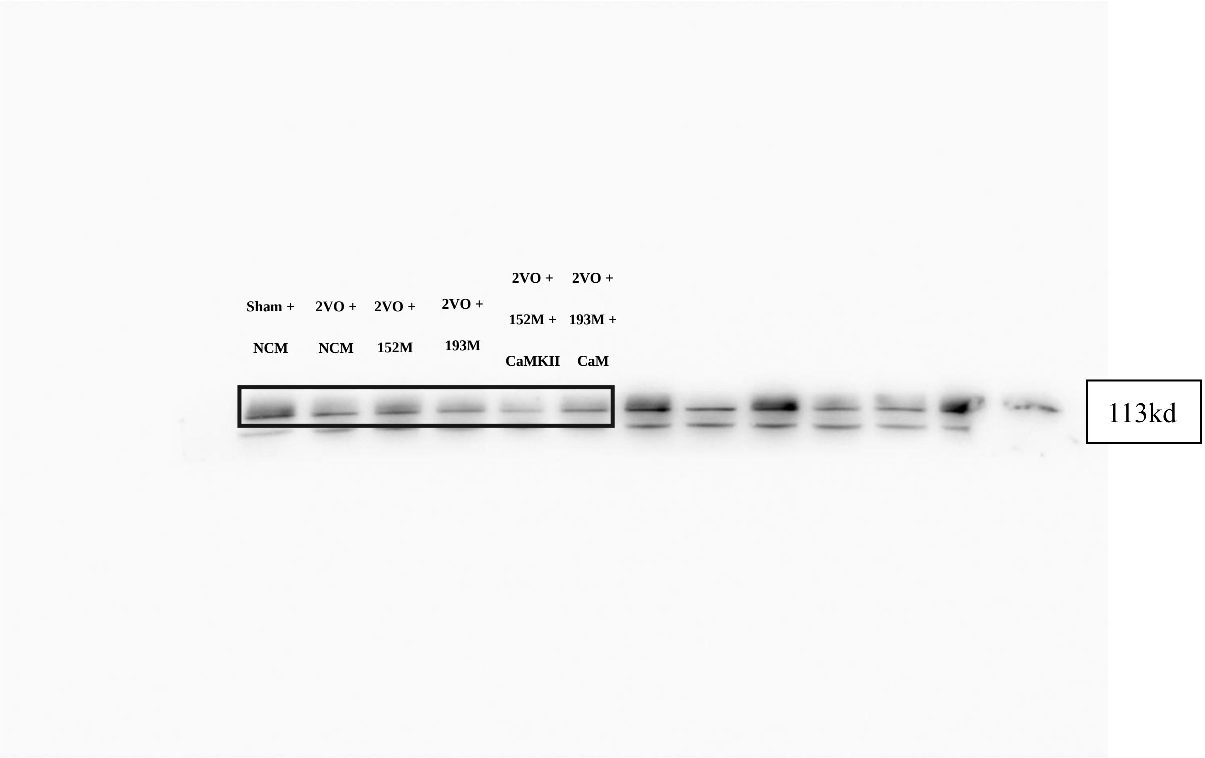
Sham + NCM
2VO + NCM
2VO + 152M
2VO + 193M
2VO + 152M + CaMKII
2VO + 193M + CaM
113kd

PARP

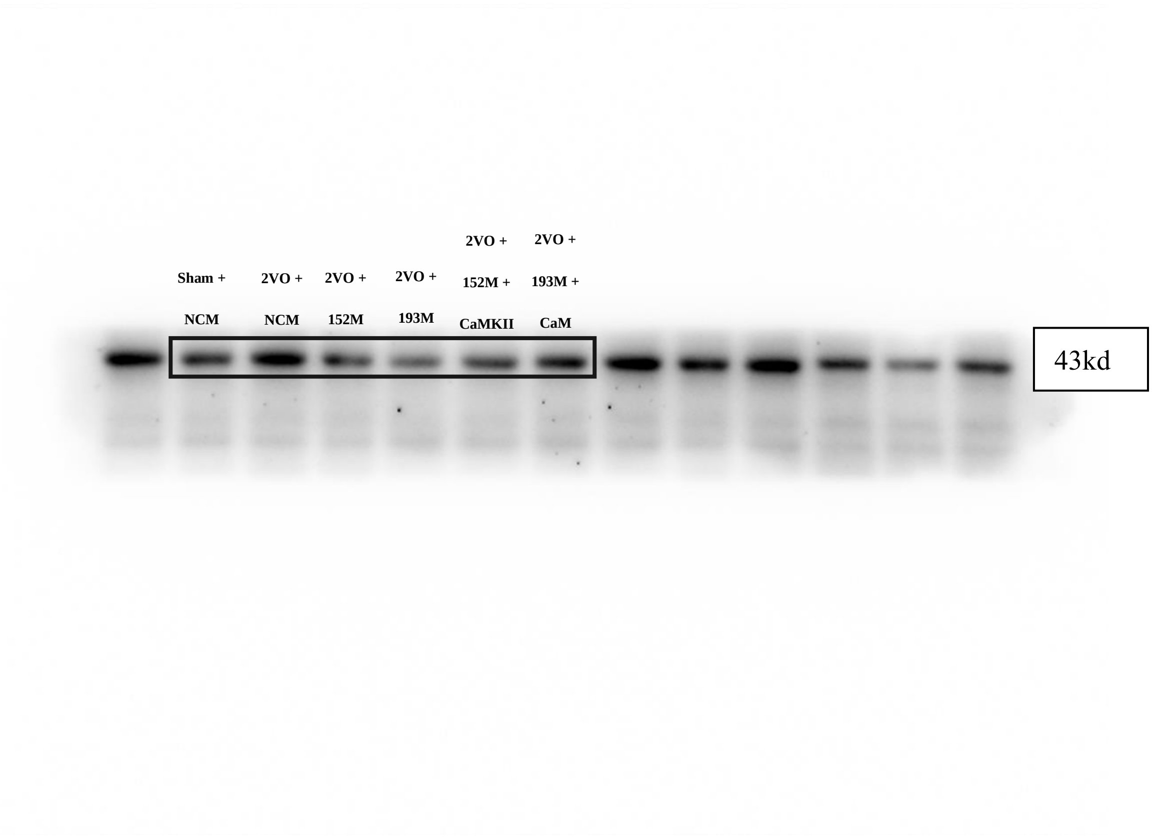
Sham + NCM
2VO + NCM
2VO + 152M
2VO + 193M
2VO + 152M + CaMKII
2VO + 193M + CaM
43kd

p-p38

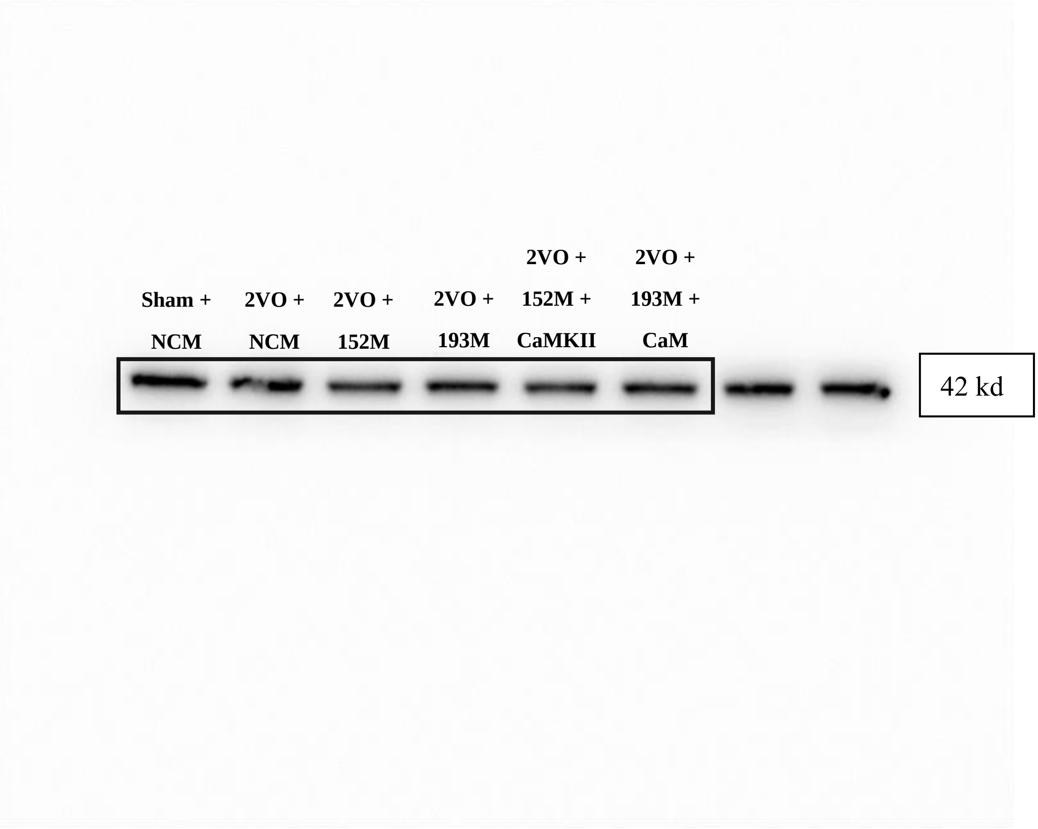
Sham + NCM
2VO + NCM
2VO + 152M
2VO + 193M
2VO + 152M + CaMKII
2VO + 193M + CaM
42 kd

p38

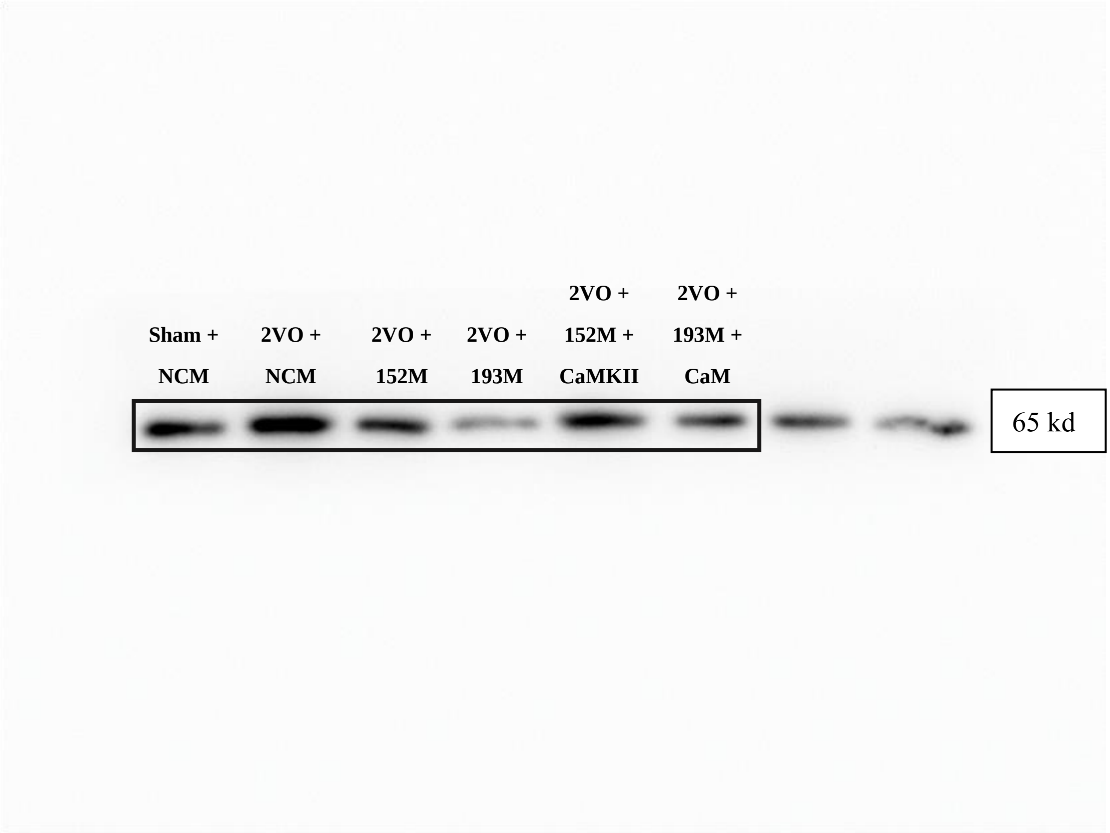
Sham + NCM
2VO + NCM
2VO + 152M
2VO + 193M
2VO + 152M + CaMKII
2VO + 193M + CaM
65 kd

p-p65

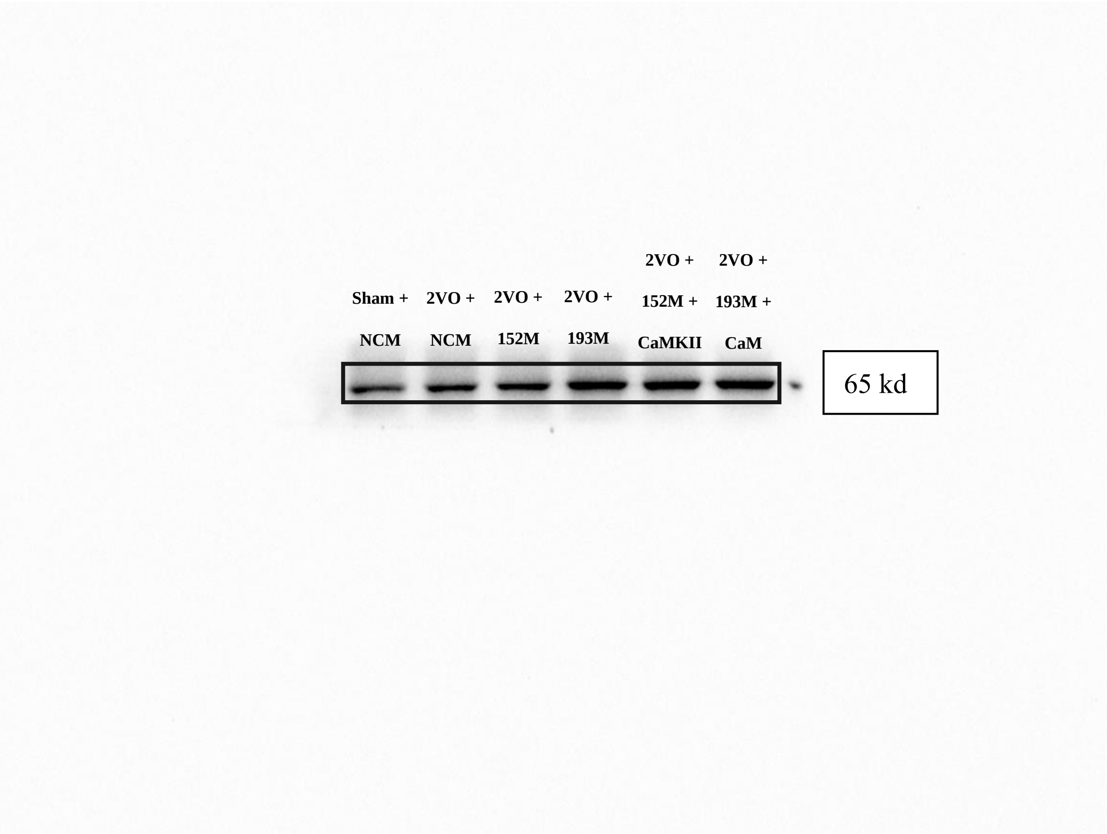
Sham + NCM
2VO + NCM
2VO + 152M
2VO + 193M
2VO + 152M + CaMKII
2VO + 193M + CaM
65 kd

p65

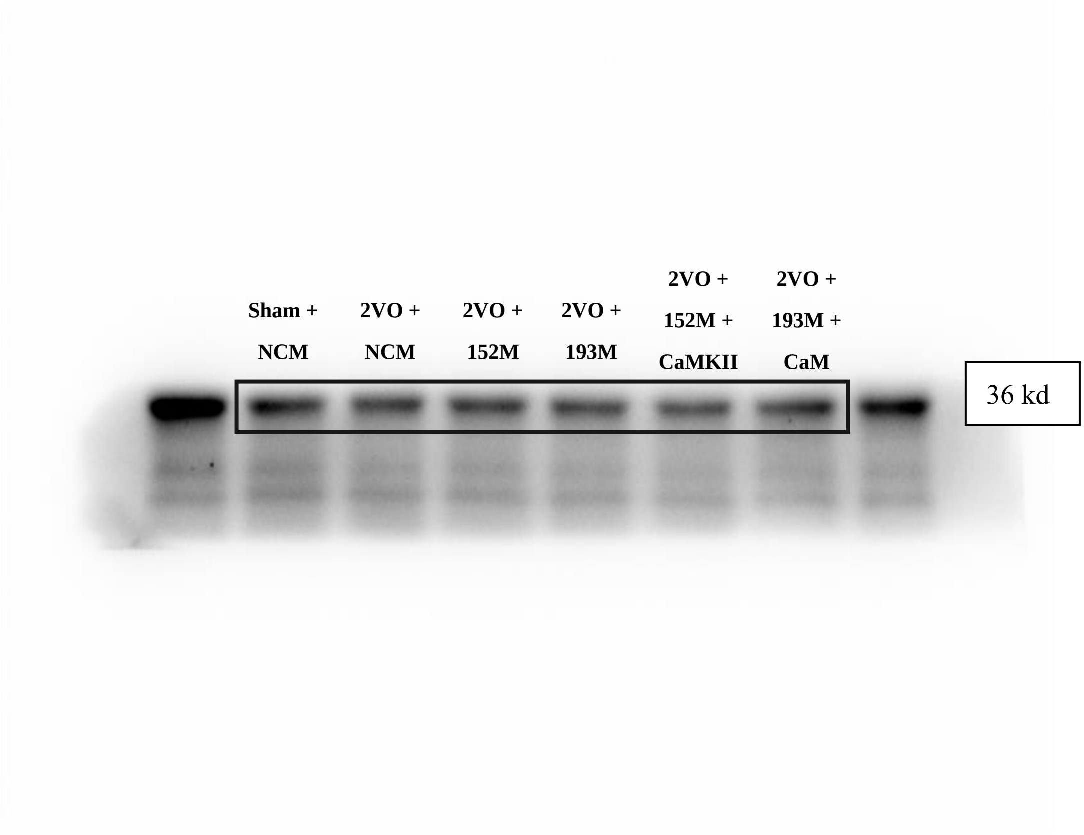
Sham + NCM
2VO + NCM
2VO + 152M
2VO + 193M
2VO + 152M + CaMKII
2VO + 193M + CaM
36 kd

GAPDH

Images in Figure 11 J

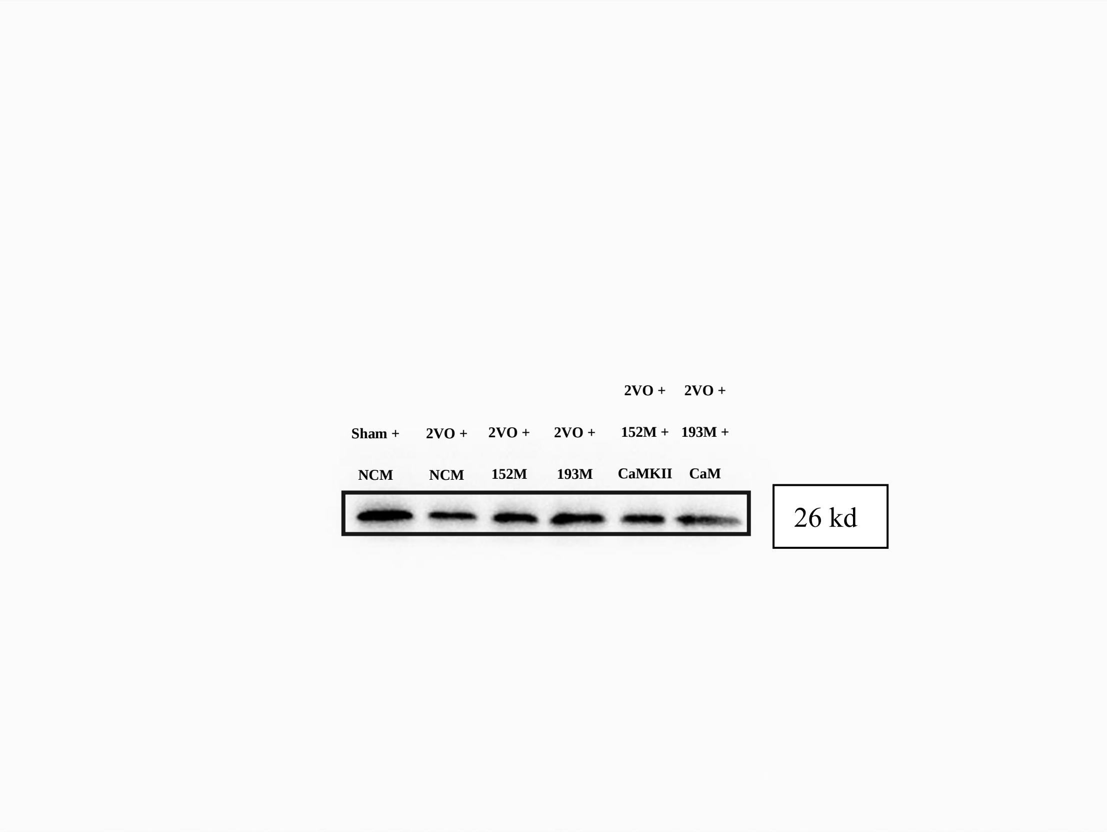

Bcl-2

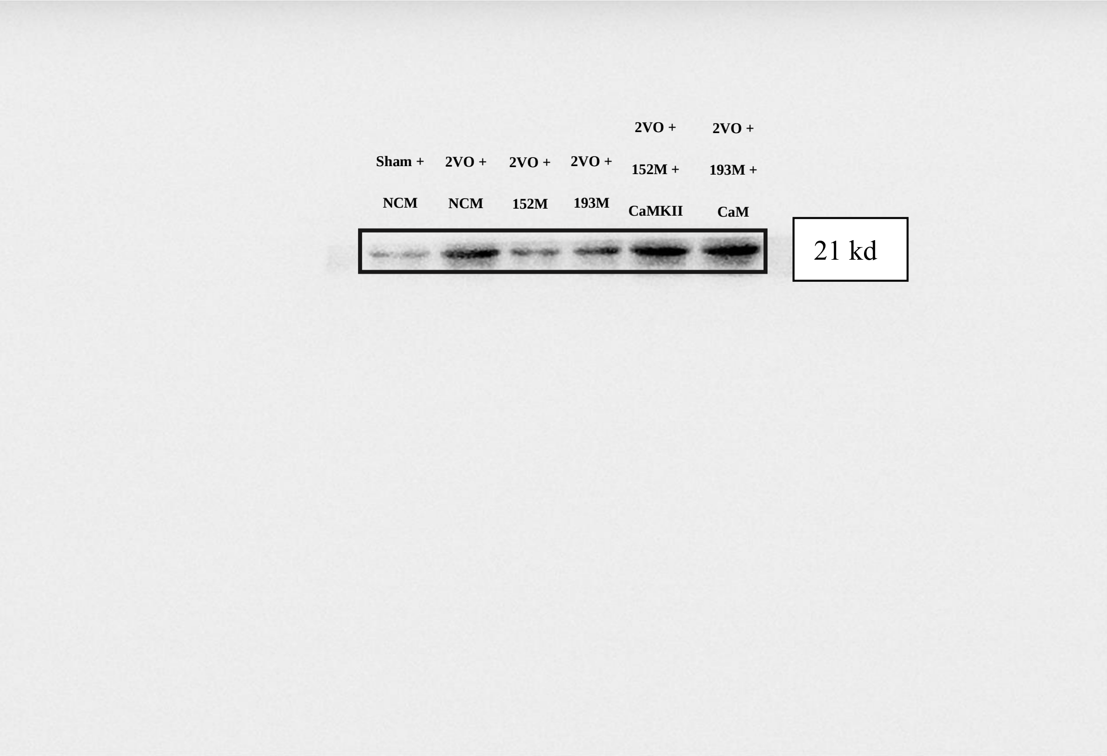

Bax

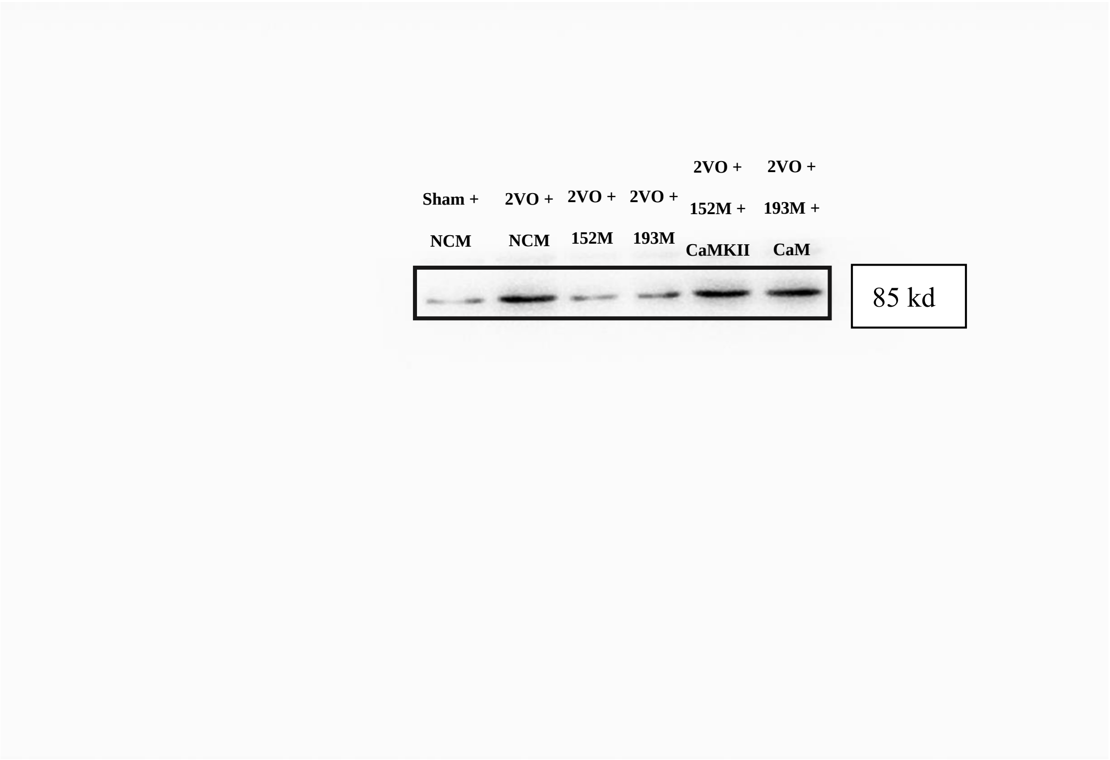
Sham + NCM
2VO + NCM
2VO + 152M
2VO + 193M
2VO + 152M + CaMKII
2VO + 193M + CaM
85 kd

c-PARP

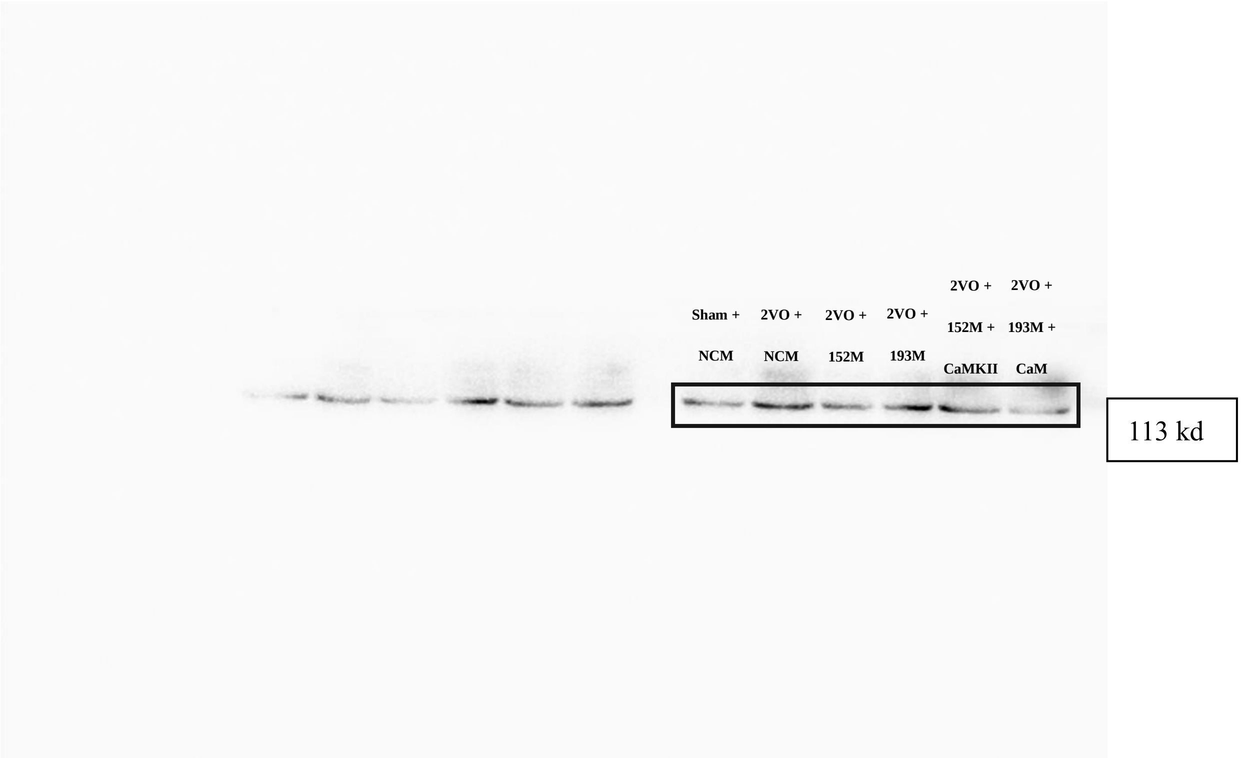
Sham + NCM
2VO + NCM
2VO + 152M
2VO + 193M
2VO + 152M + CaMKII
2VO + 193M + CaM
113 kd

PARP

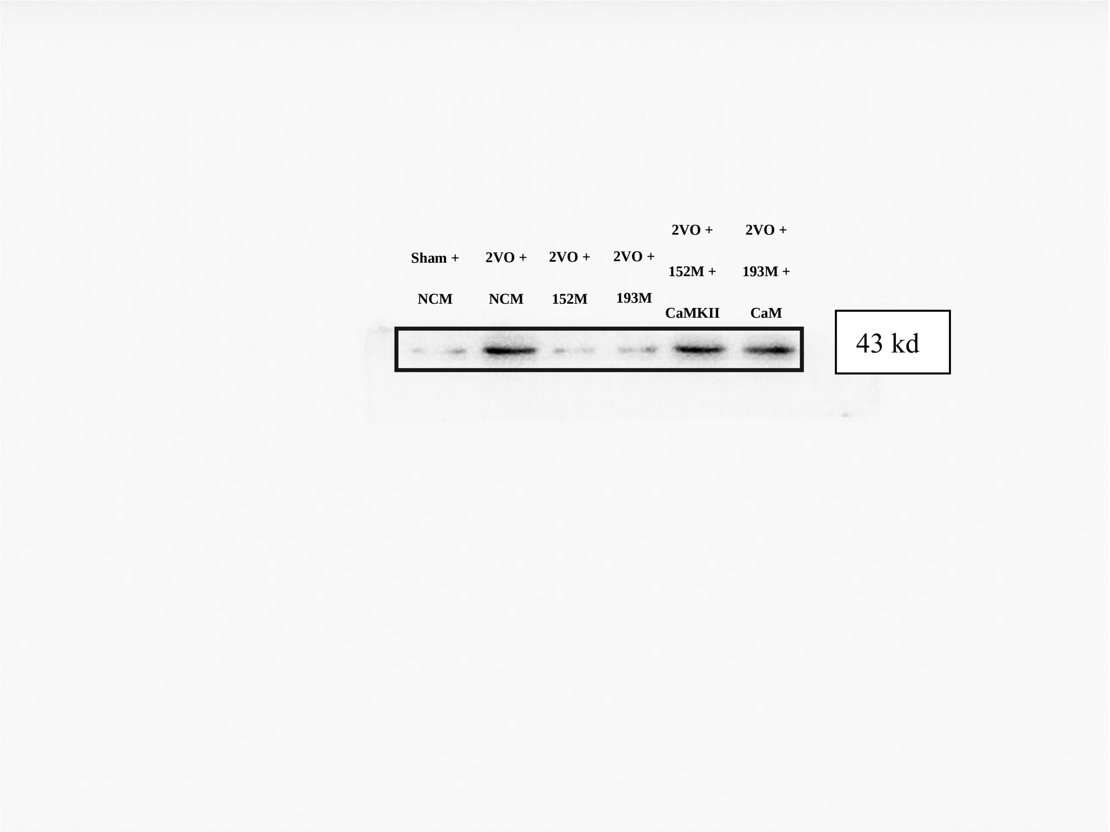
Sham +
NCM
2VO +
NCM
2VO +
152M
2VO +
193M
2VO +
152M +
CaMKII
2VO +
193M +
CaM
43 kd

p-p38

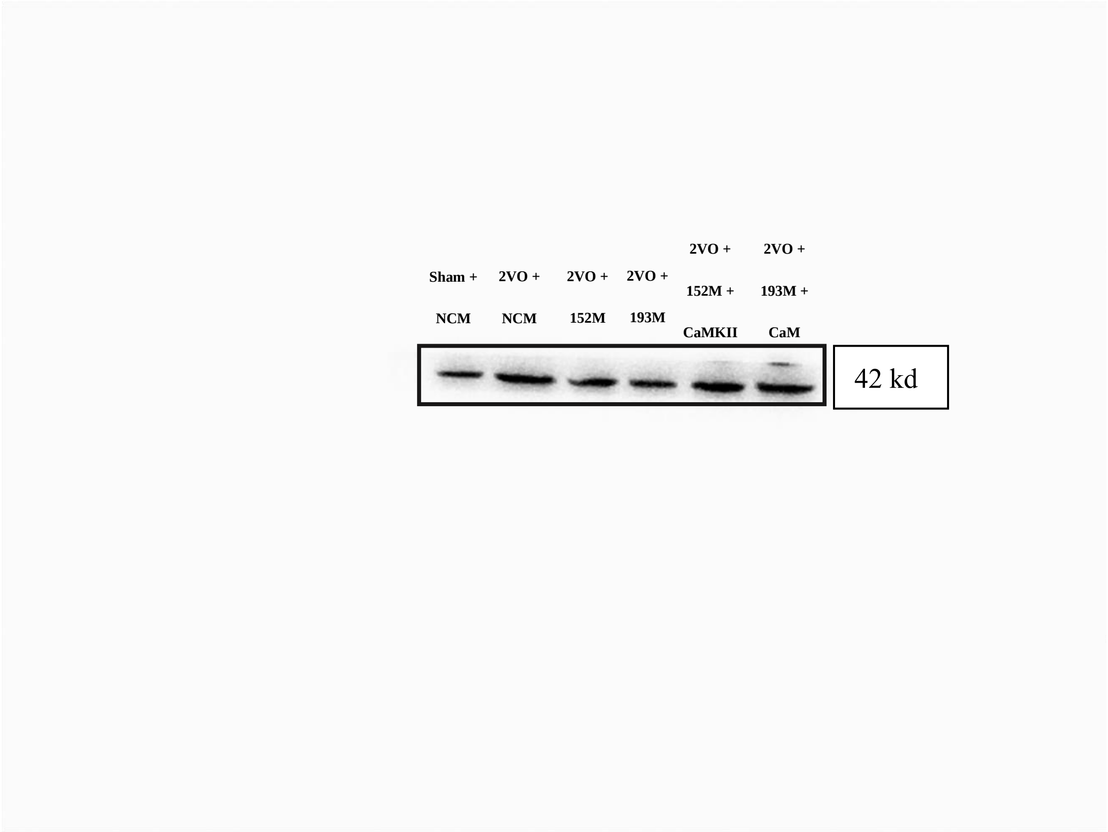
Sham +
NCM
2VO +
NCM
2VO +
152M
2VO +
193M
2VO +
152M +
CaMKII
2VO +
193M +
CaM
42 kd

p38

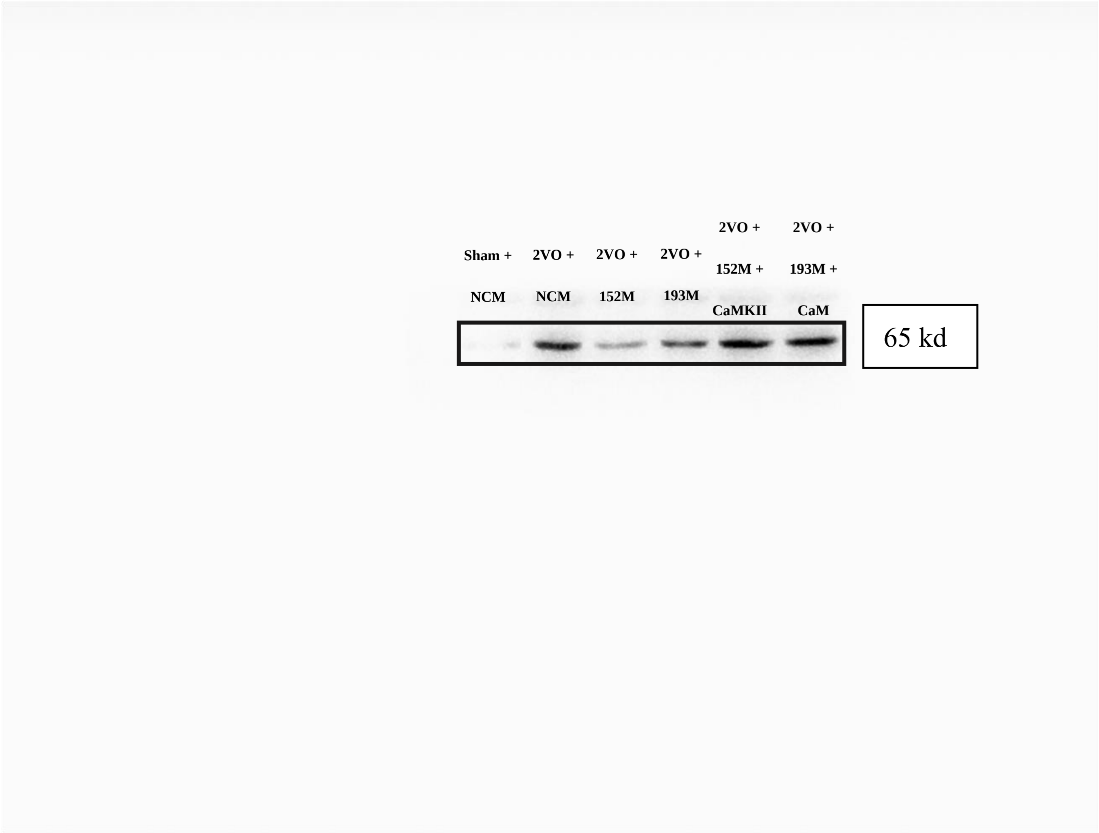
Sham + NCM
2VO + NCM
2VO + 152M
2VO + 193M
2VO + 152M + CaMKII
2VO + 193M + CaM
65 kd

p-p65

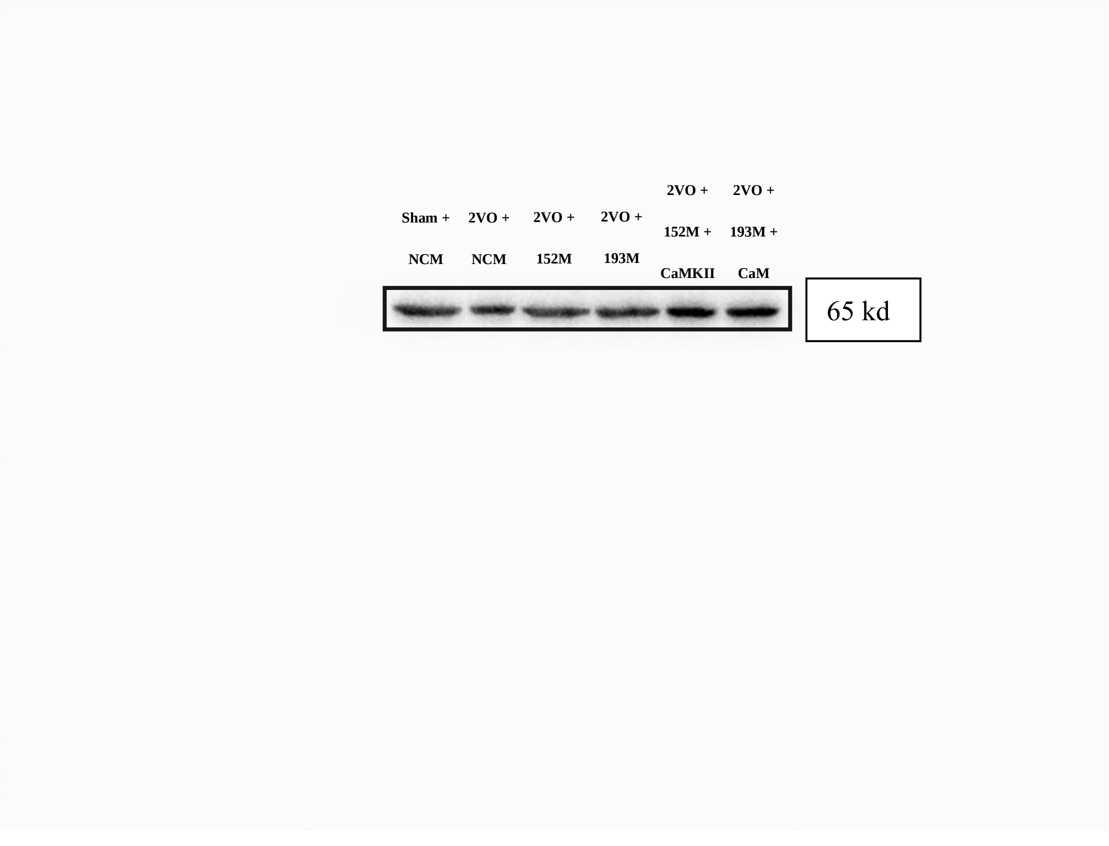
Sham + NCM
2VO + NCM
2VO + 152M
2VO + 193M
2VO + 152M + CaMKII
2VO + 193M + CaM
65 kd

p65

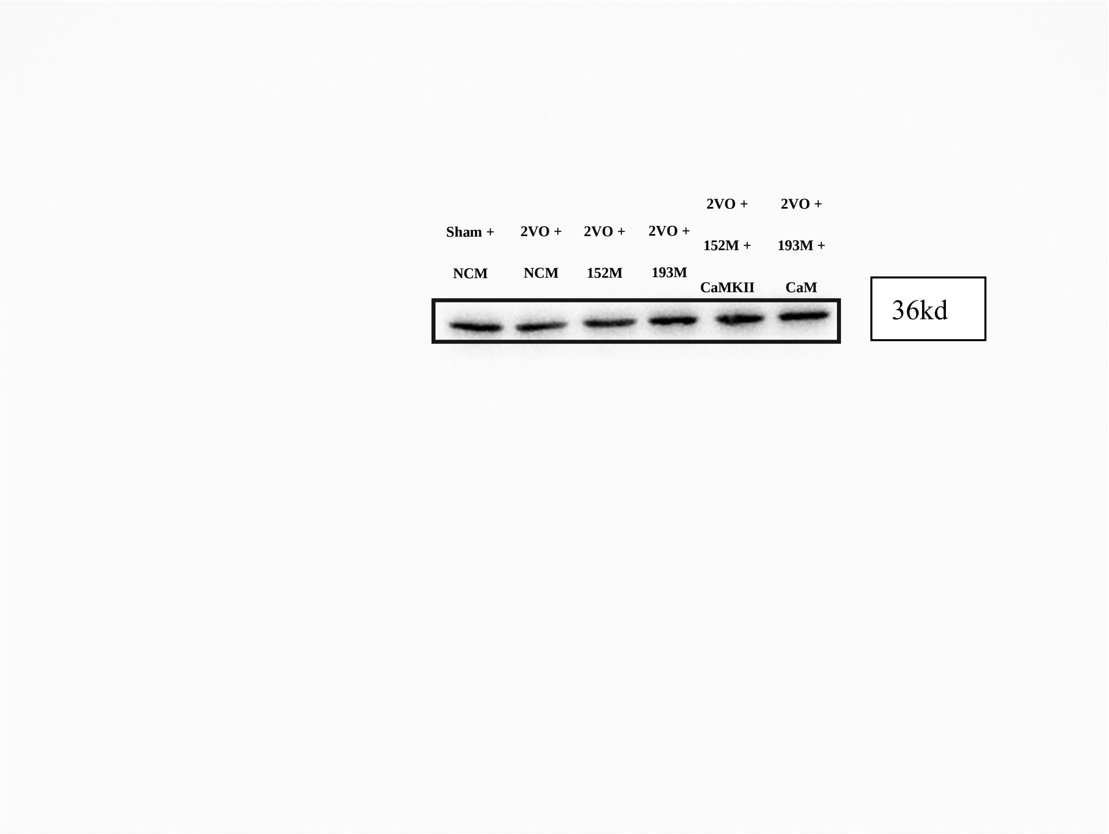
Sham + NCM
2VO + NCM
2VO + 152M
2VO + 193M
2VO + 152M + CaMKII
2VO + 193M + CaM
36kd

GAPDH

Images in Figure 14 A

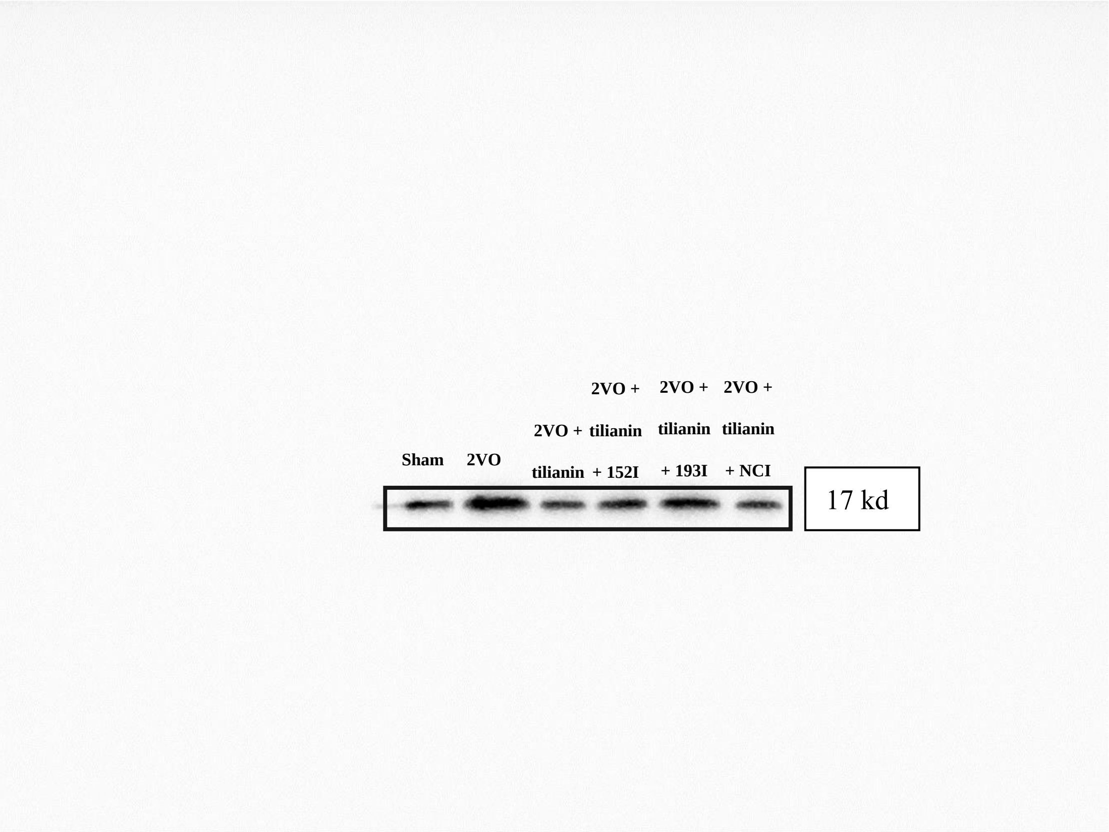

CaM

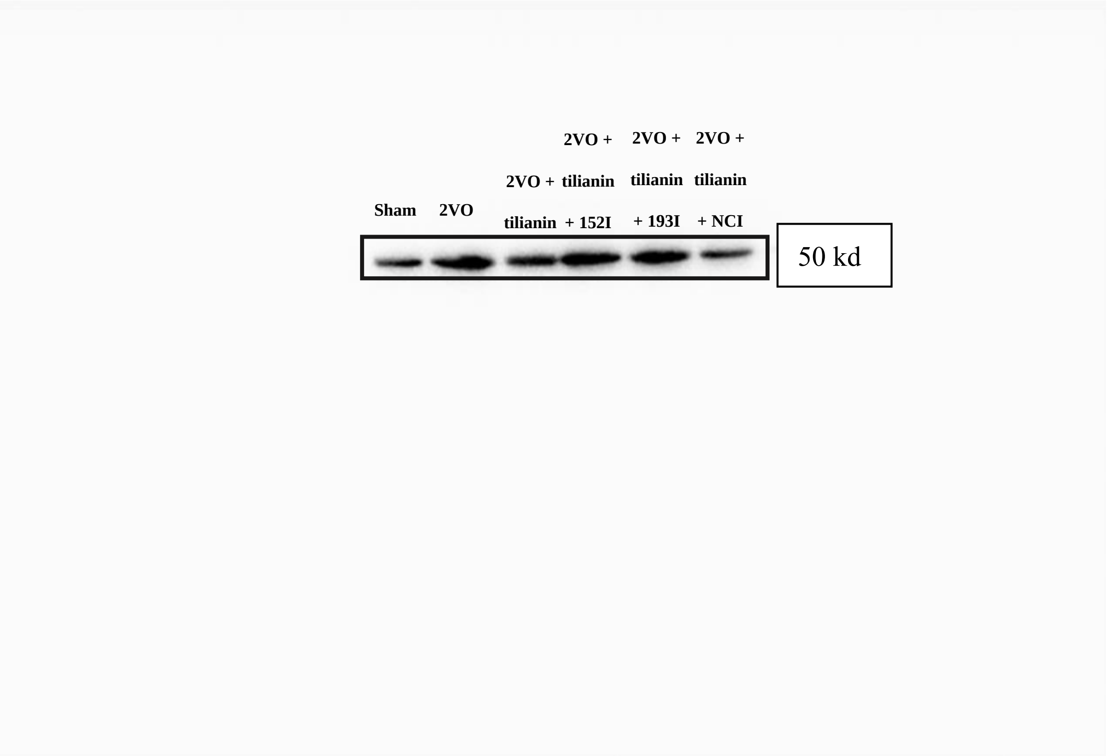

ox-CaMKIIα

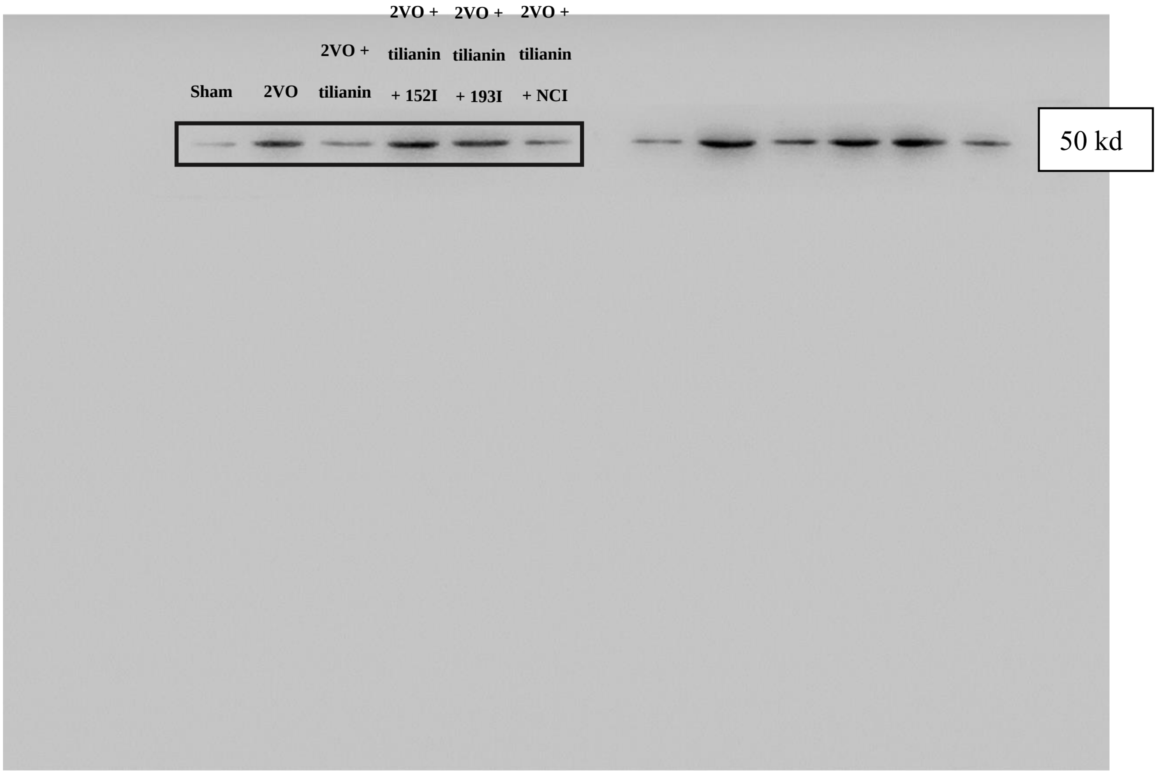

p-CaMKIIα

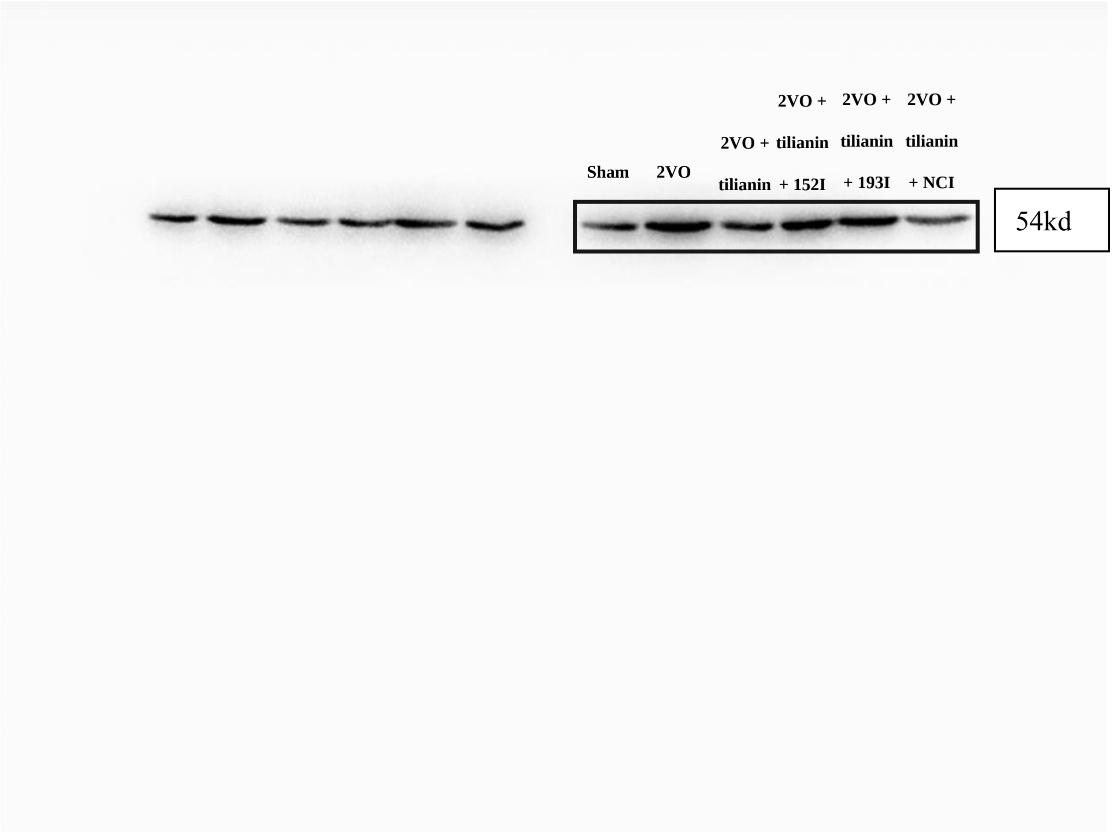

CaMKIIα

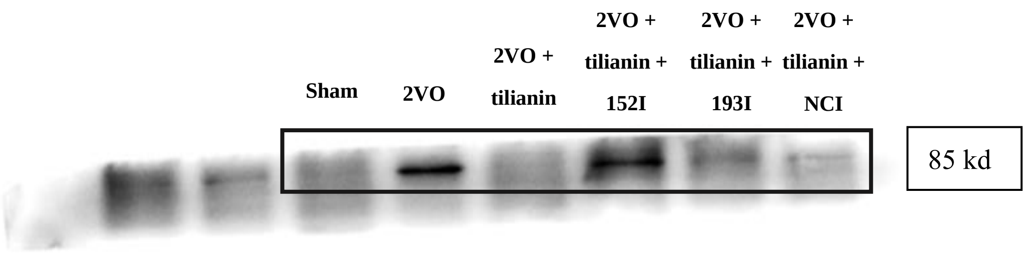
Sham
2VO
2VO + tilianin
2VO + tilianin + 152I
2VO + tilianin + 193I
2VO + tilianin + NCI
85 kd

c-PARP

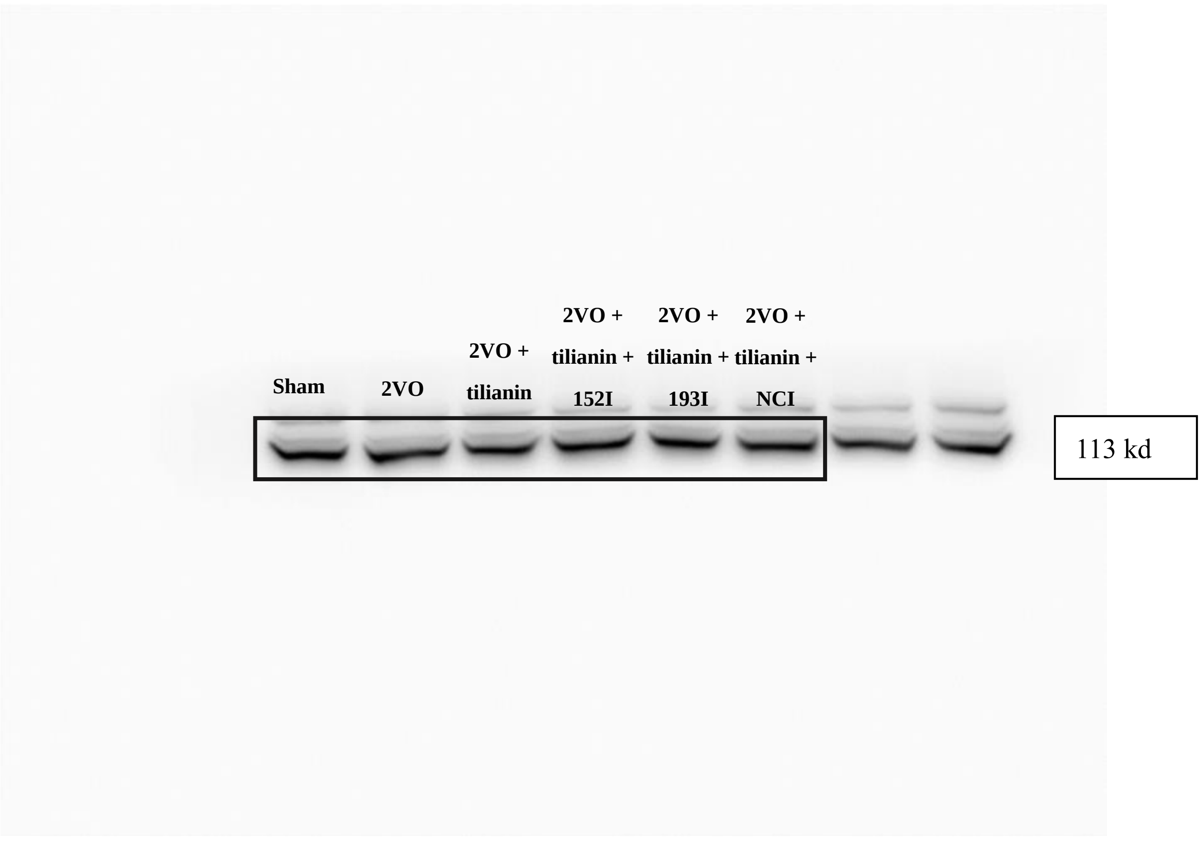
Sham
2VO
2VO + tilianin
2VO + tilianin + 152I
2VO + tilianin + 193I
2VO + tilianin + NCI
113 kd

PARP

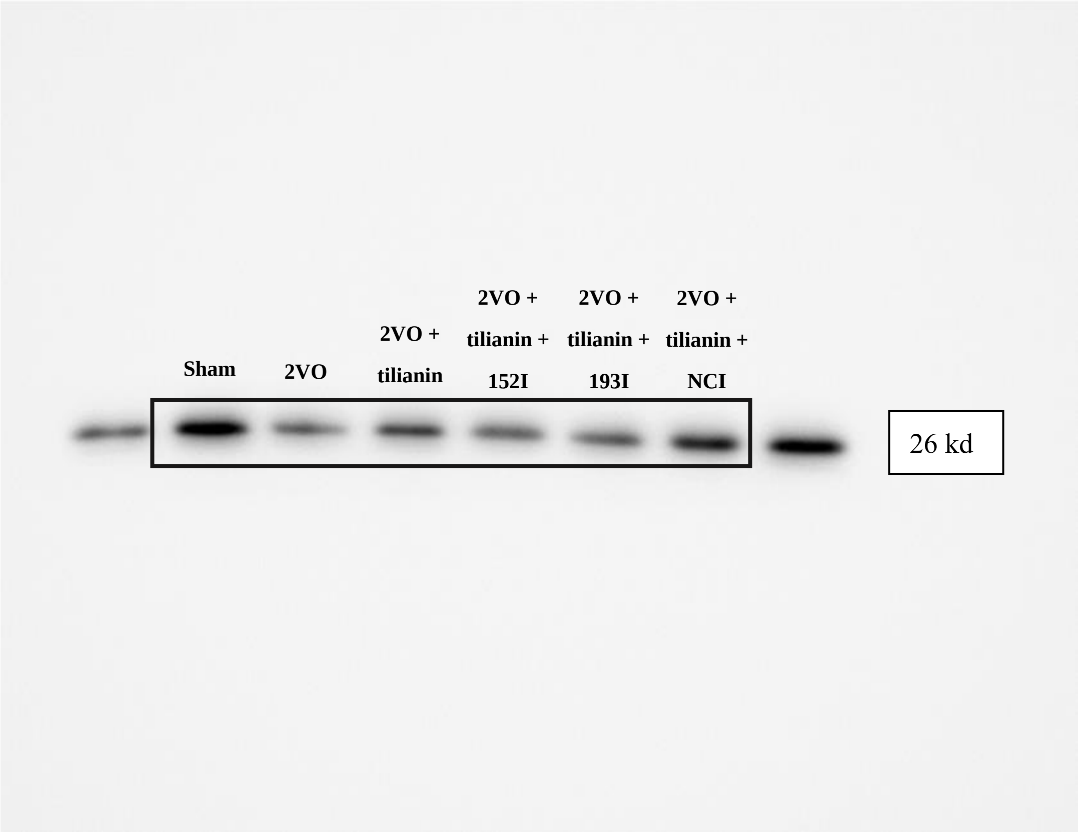
Sham
2VO
2VO + tilianin
2VO + tilianin + 152I
2VO + tilianin + 193I
2VO + tilianin + NCI
26 kd

Bcl-2

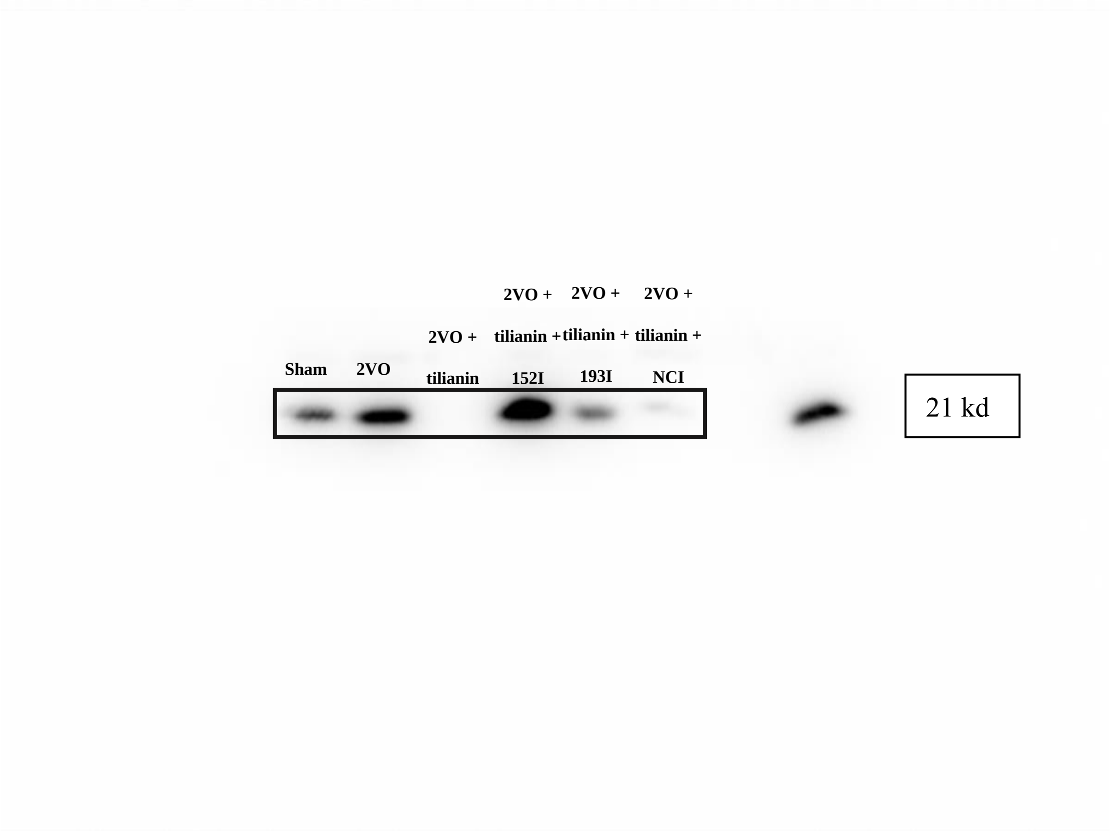
Sham
2VO
2VO + tilianin
2VO + tilianin + 152I
2VO + tilianin + 193I
2VO + tilianin + NCI
21 kd

Bax

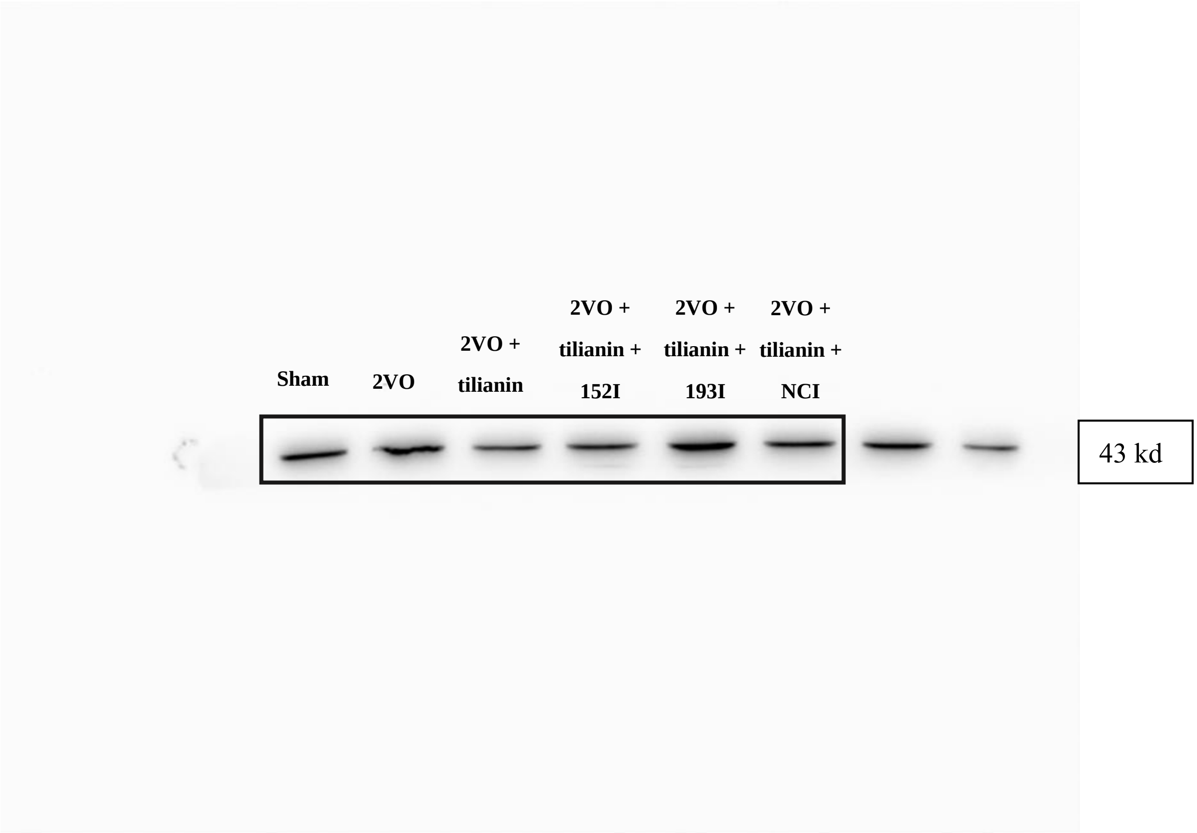
Sham
2VO
2VO + tilianin
2VO + tilianin + 152I
2VO + tilianin + 193I
2VO + tilianin + NCI
43 kd

p-p38

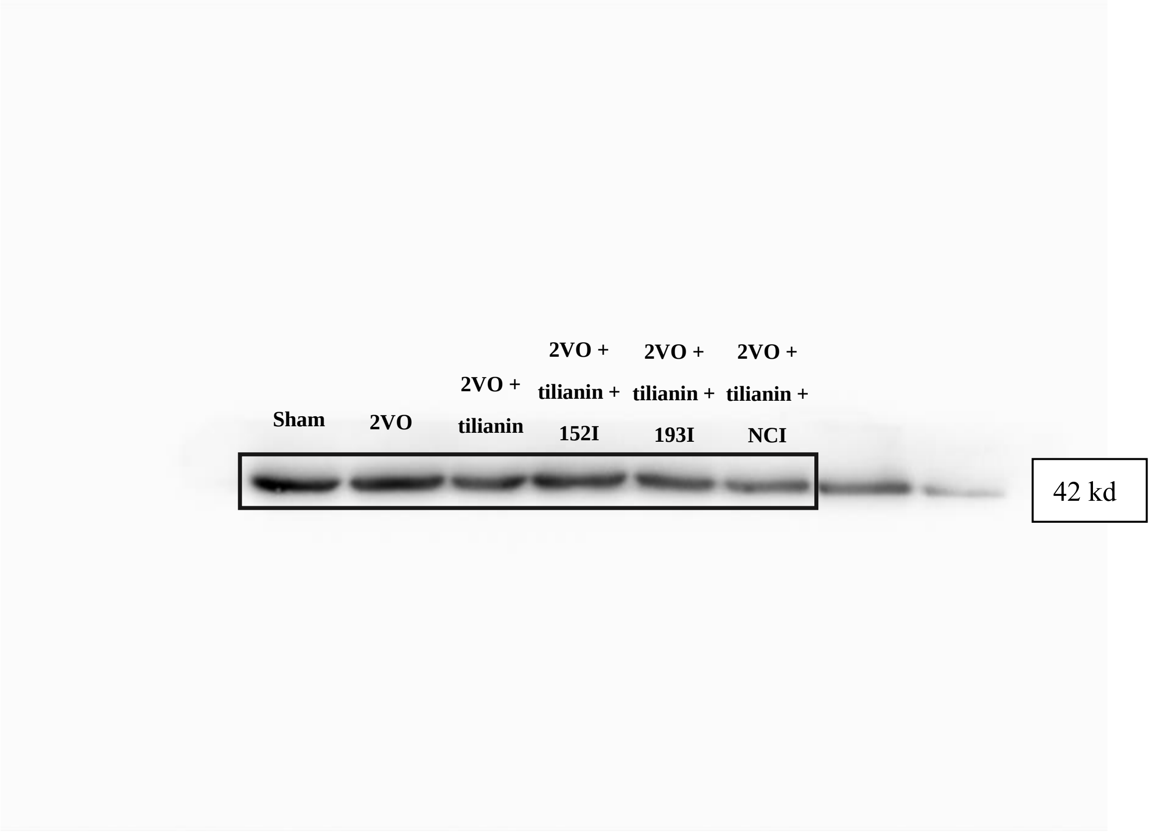
Sham
2VO
2VO + tilianin
2VO + tilianin + 152I
2VO + tilianin + 193I
2VO + tilianin + NCI
42 kd

p38

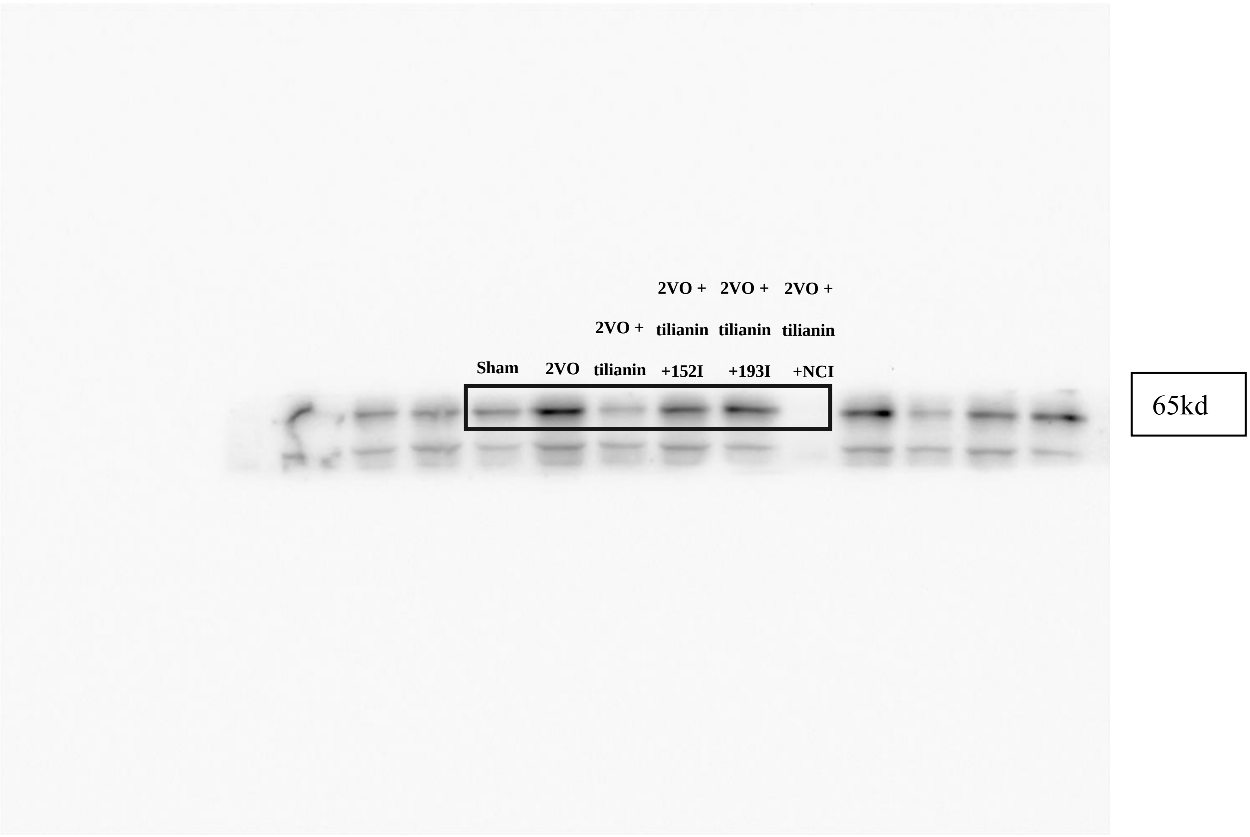
Sham
2VO
2VO + tilianin
2VO + tilianin +152I
2VO + tilianin +193I
2VO + tilianin +NCI
65kd

p-p65

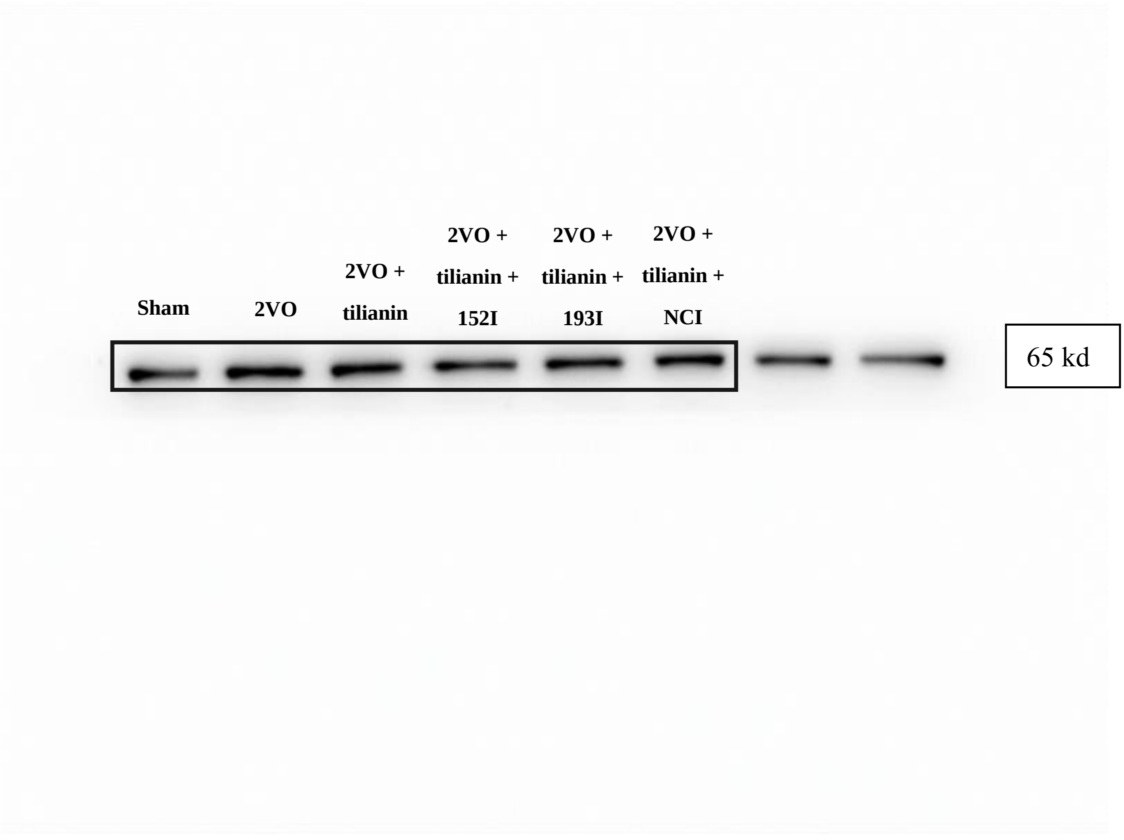
Sham
2VO
2VO + tilianin
2VO + tilianin + 152I
2VO + tilianin + 193I
2VO + tilianin + NCI
65 kd

p65

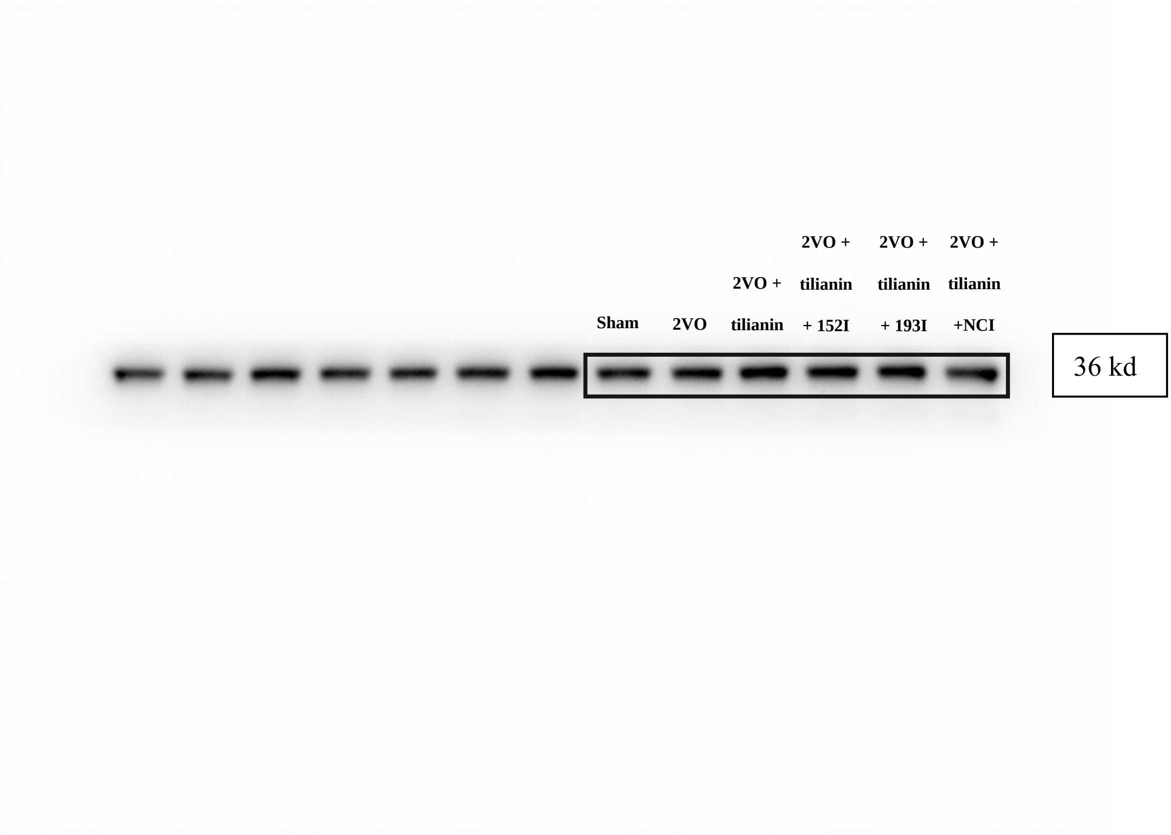
Sham
2VO
2VO + tilianin
2VO + tilianin + 152I
2VO + tilianin + 193I
2VO + tilianin +NCI
36 kd

GAPDH

Images in Figure 14 G

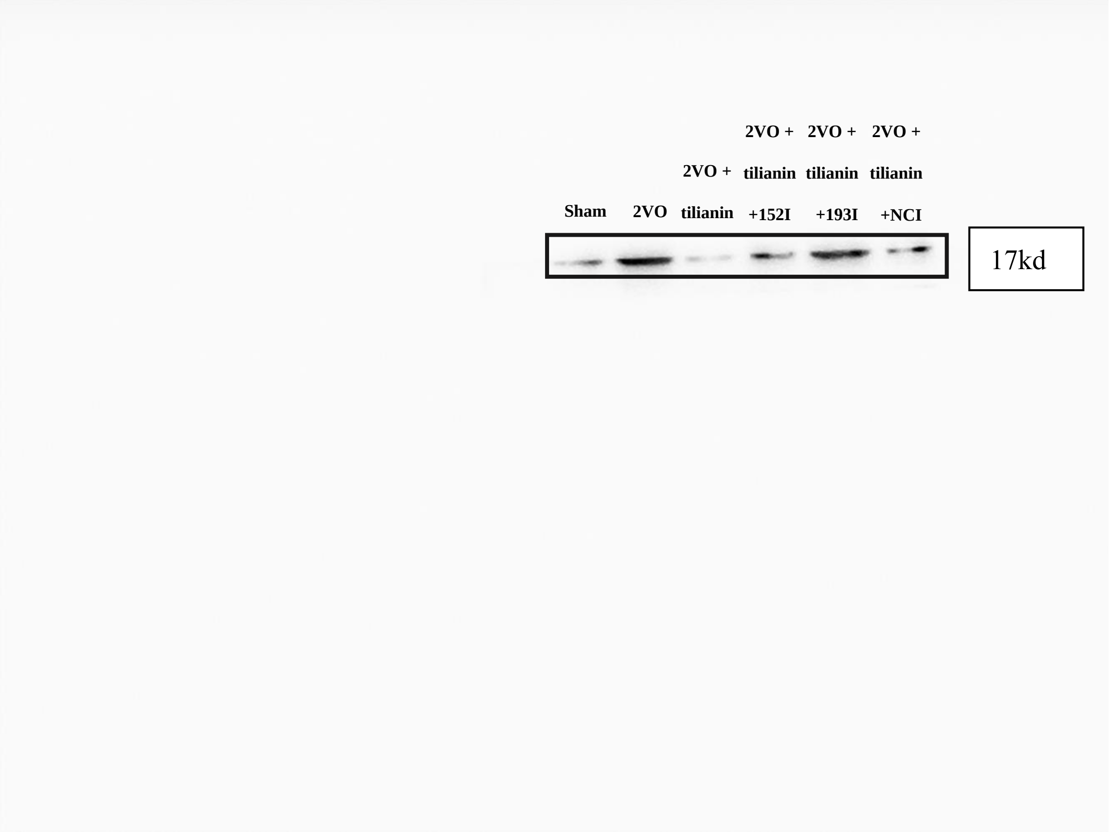

CaM

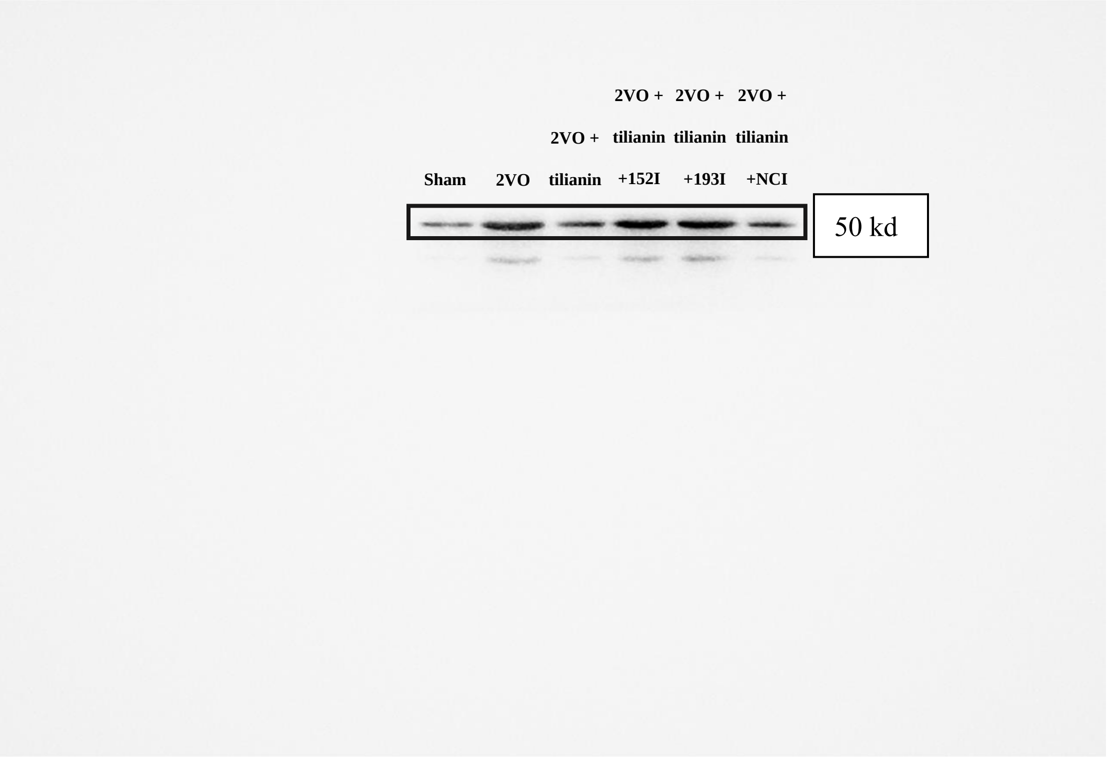

ox-CaMKIIα

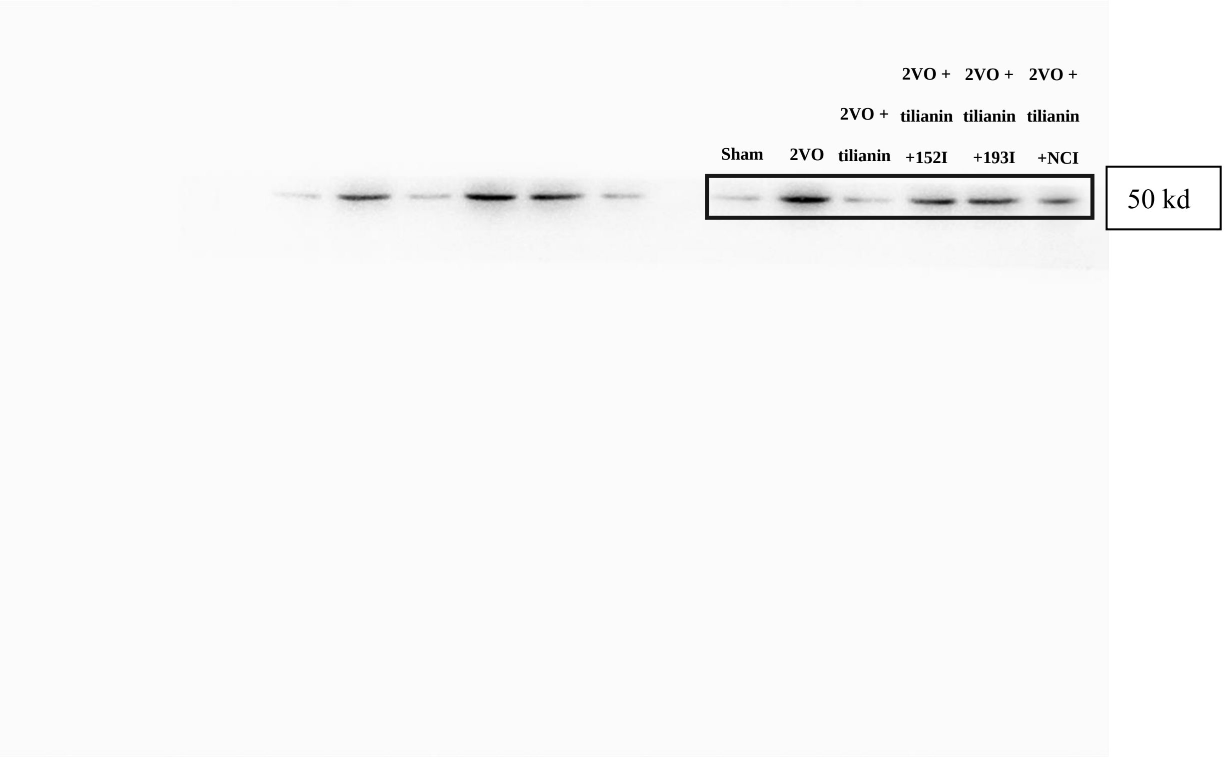

p-CaMKIIα

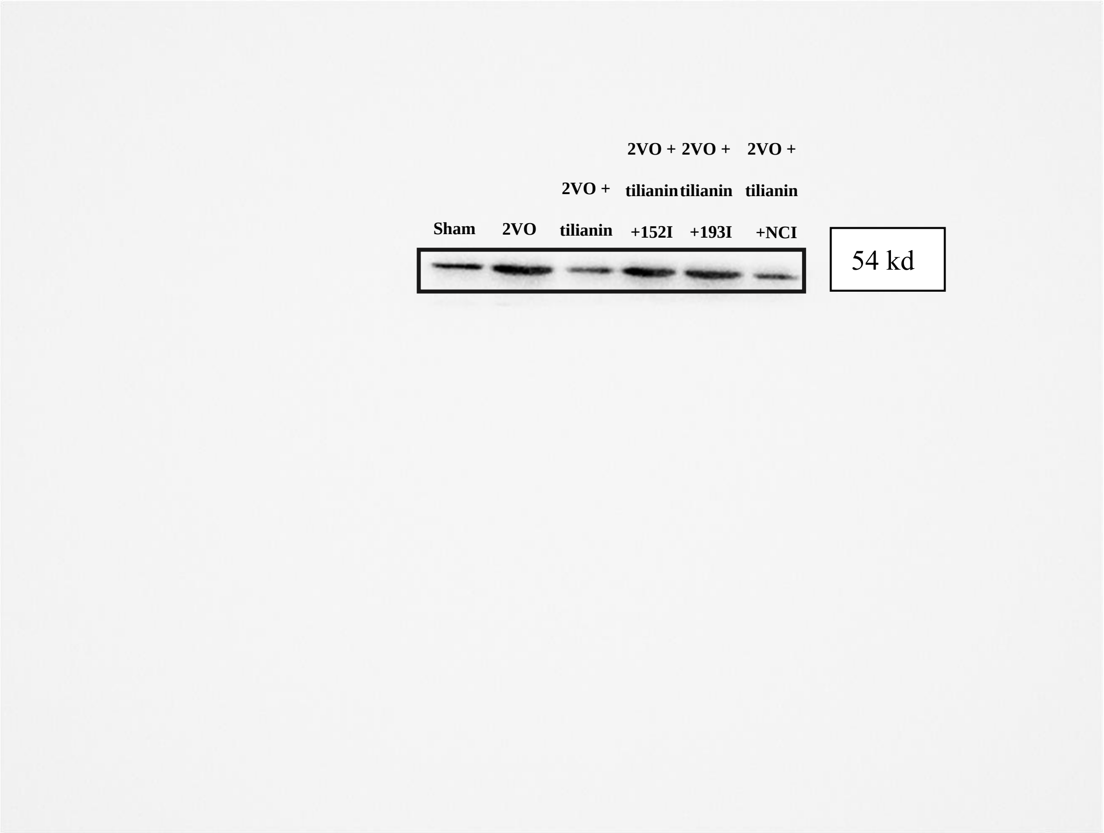

CaMKIIα

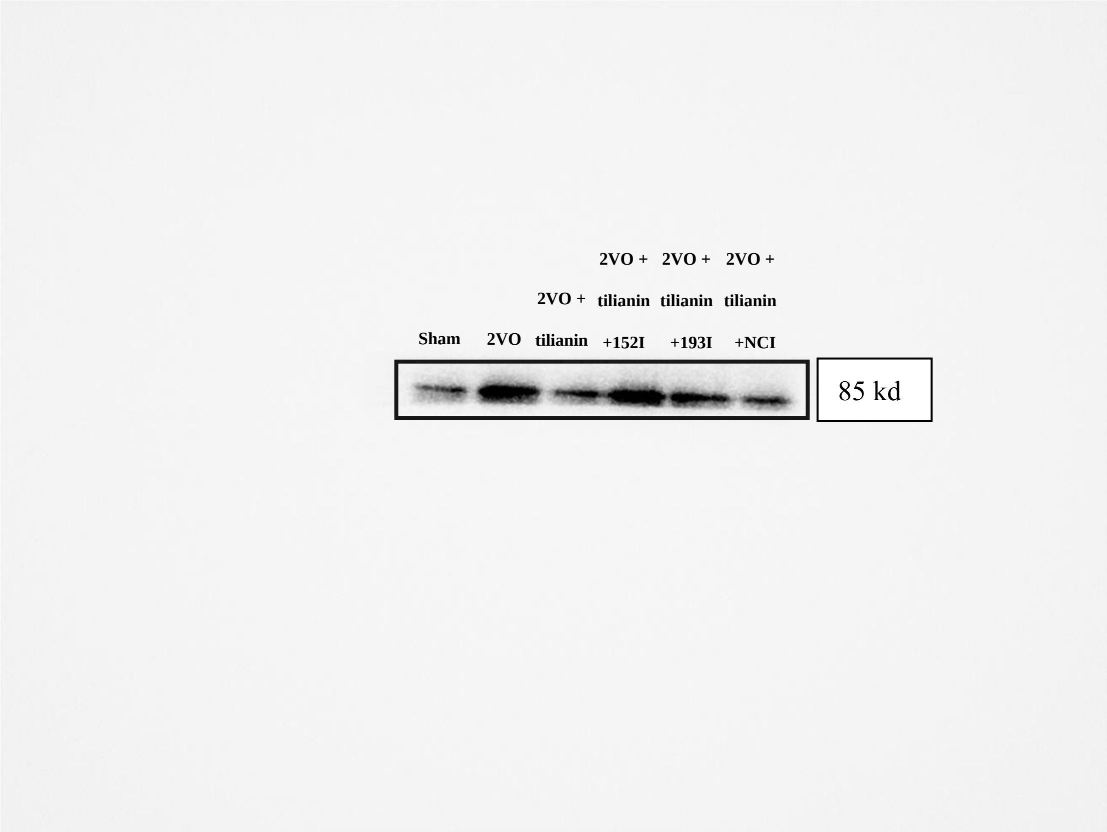
Sham
2VO
2VO + tilianin
2VO + tilianin +152I
2VO + tilianin +193I
2VO + tilianin +NCI
85 kd

c-PARP

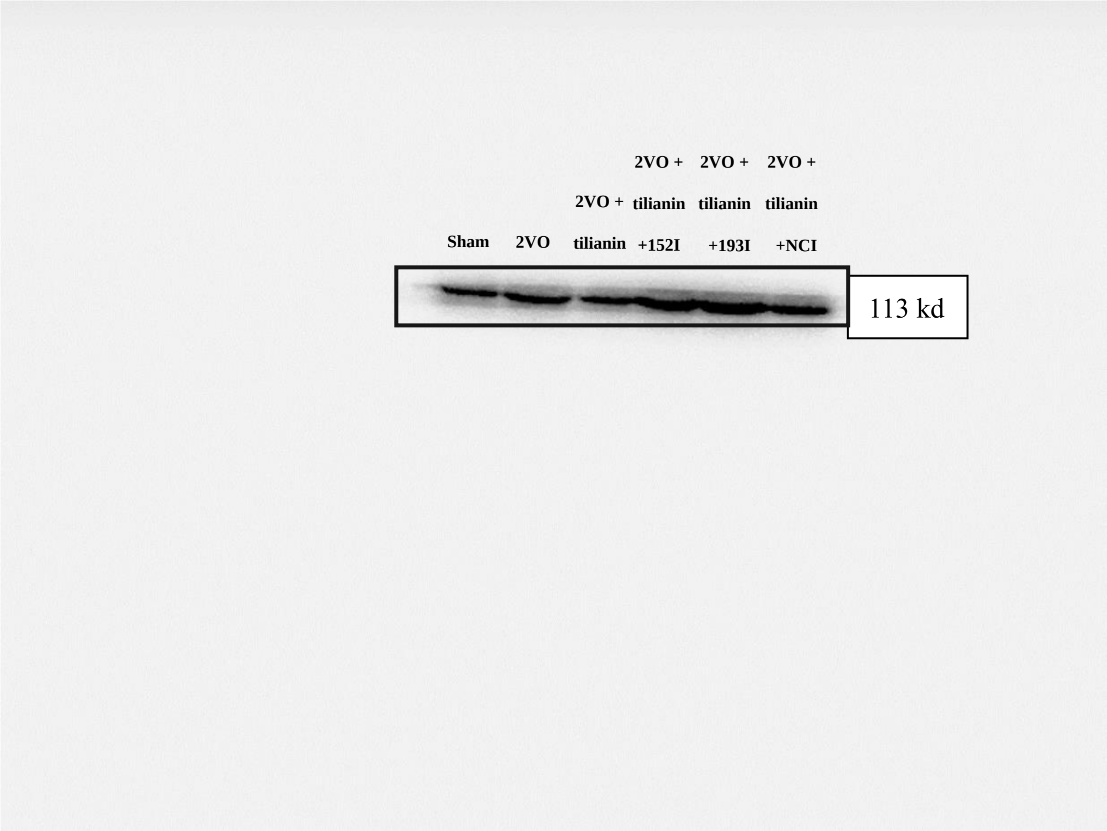
Sham
2VO
2VO + tilianin
2VO + tilianin +152I
2VO + tilianin +193I
2VO + tilianin +NCI
113 kd

PARP

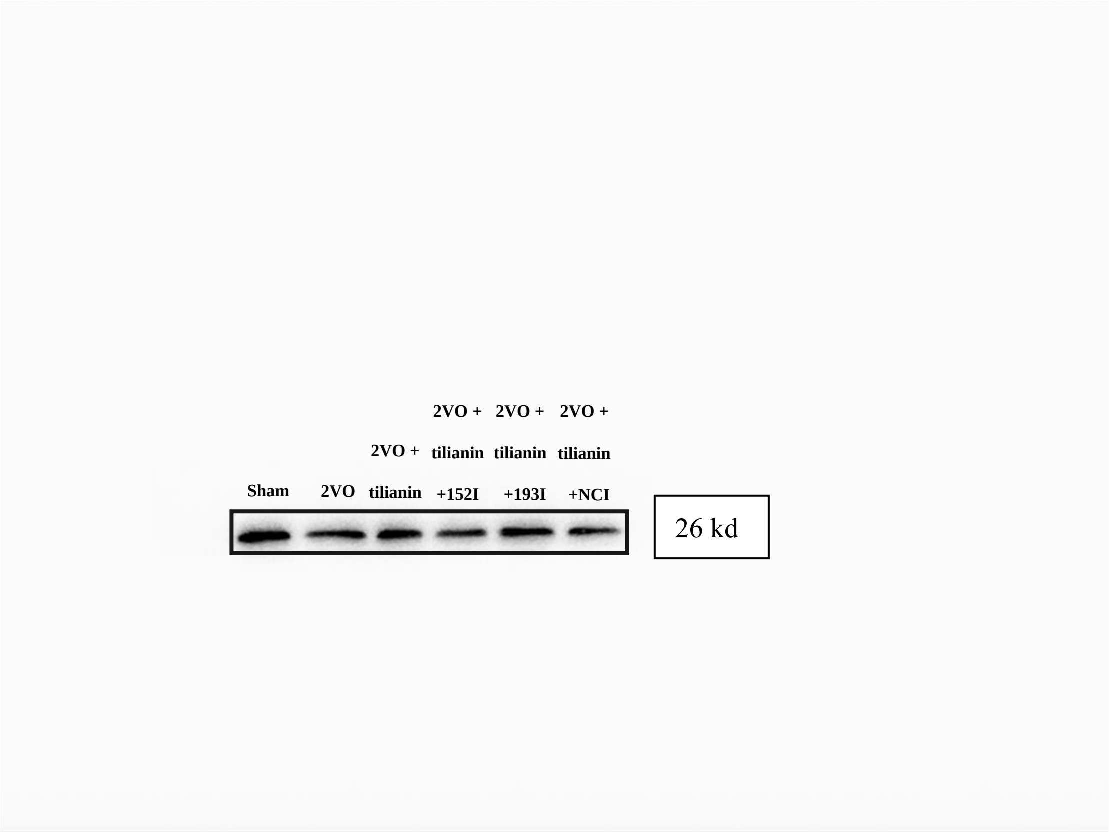
2VO +
2VO +
2VO +
2VO +
Sham
2VO
tilianin
tilianin +152I
tilianin +193I
tilianin +NCI
26 kd

Bcl-2

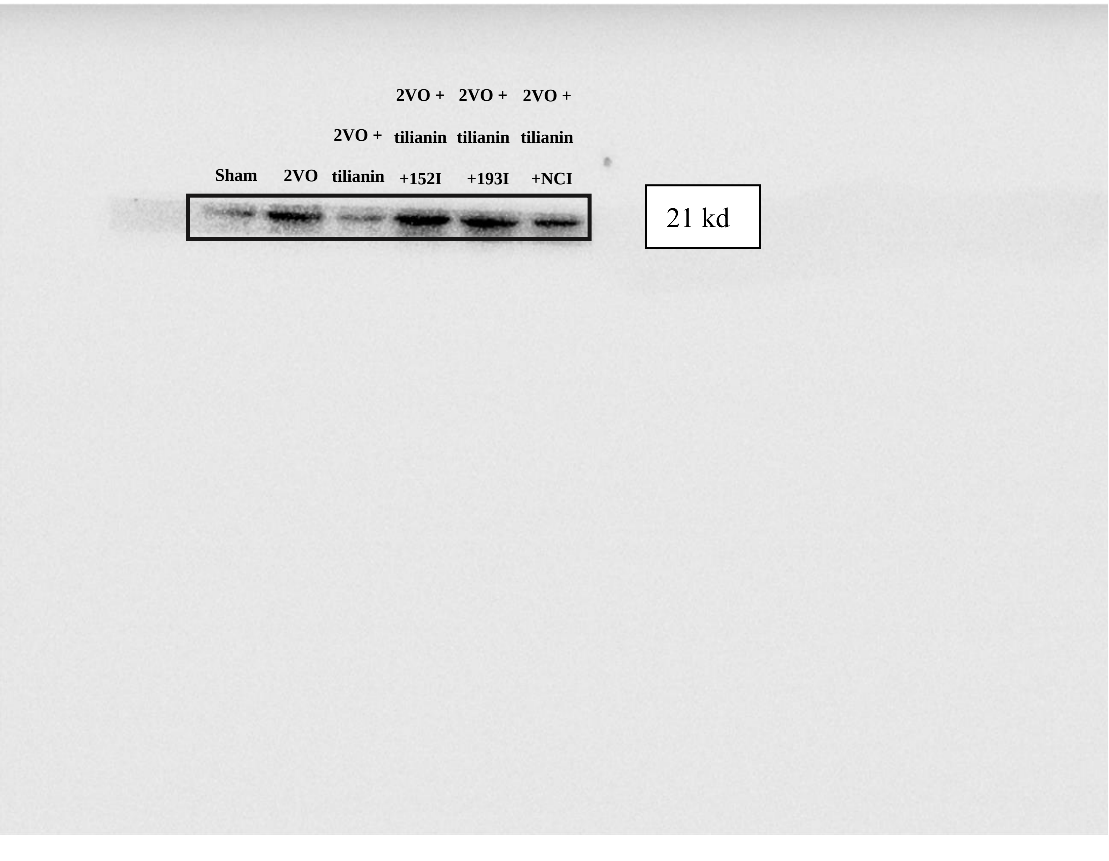
2VO +
2VO +
2VO +
2VO +
Sham
2VO
tilianin
tilianin +152I
tilianin +193I
tilianin +NCI
21 kd

Bax

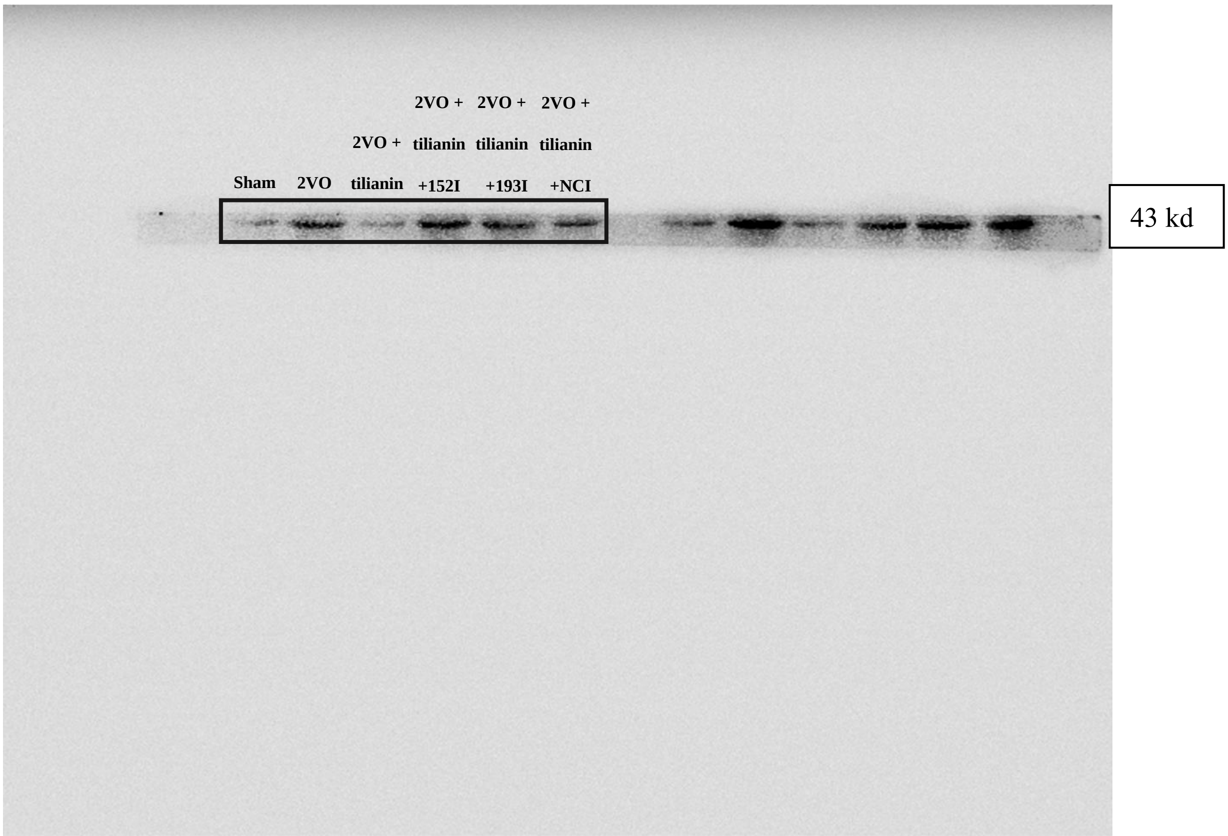
2VO +
tilianin
+152I
2VO +
tilianin
+193I
2VO +
tilianin
+NCI
Sham
2VO
2VO +
tilianin
43 kd

p-p38

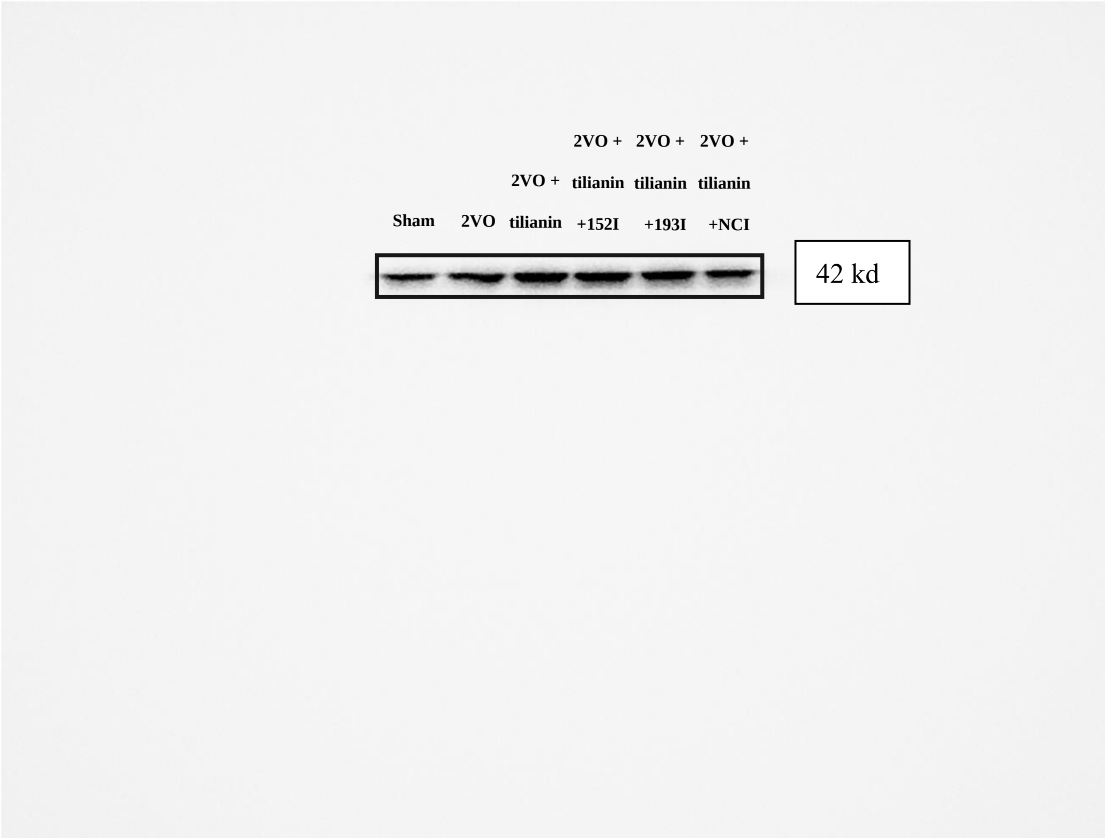
Sham
2VO
2VO +
tilianin
2VO +
tilianin
+152I
2VO +
tilianin
+193I
2VO +
tilianin
+NCI
42 kd

p38

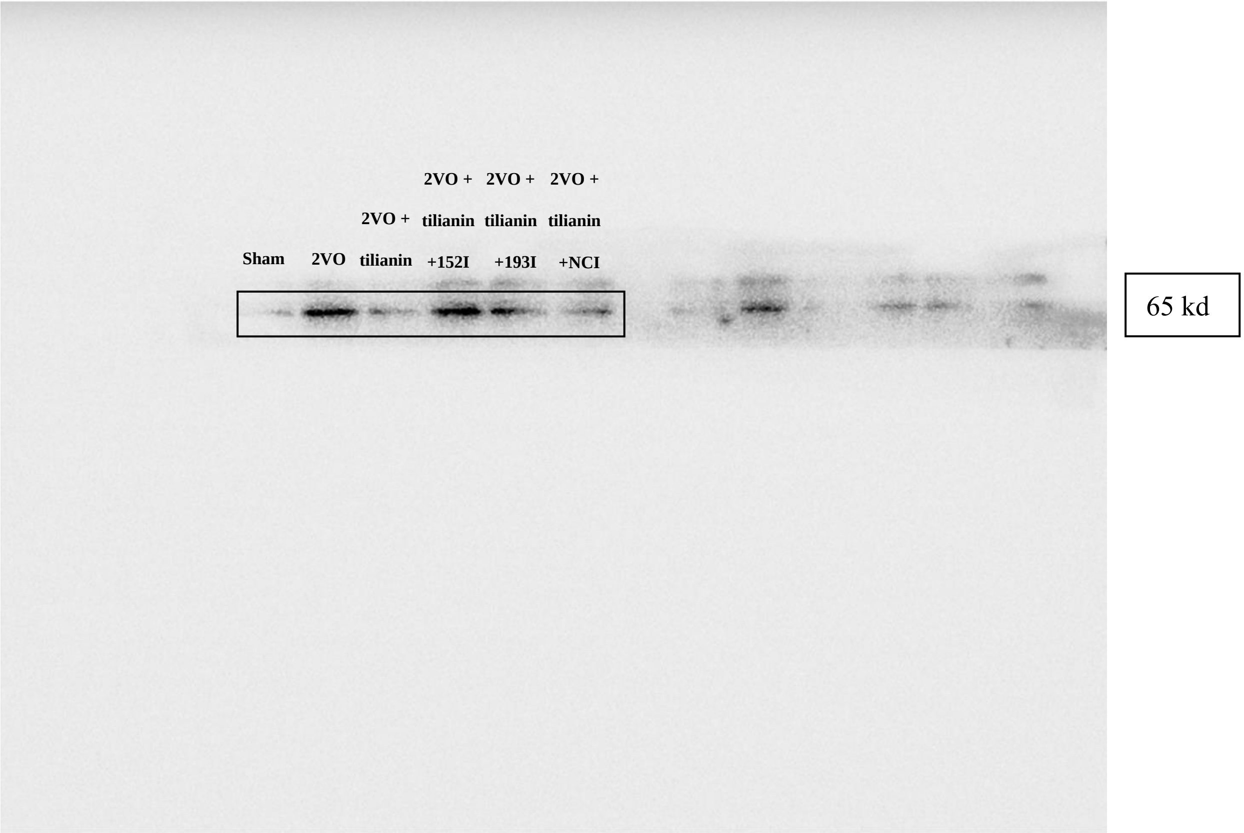
2VO + 2VO + 2VO +
2VO + tilianin tilianin tilianin
Sham 2VO tilianin +152I +193I +NCI
65 kd

p-p65

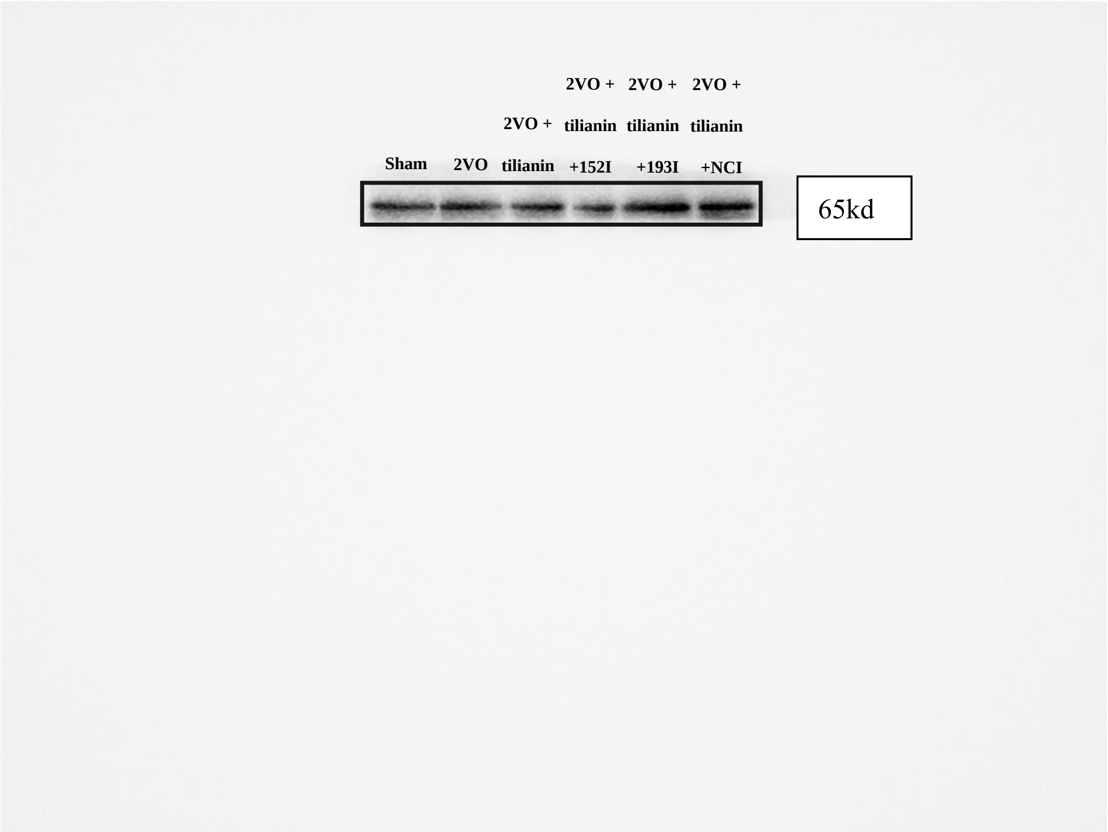
2VO + 2VO + 2VO +
2VO + tilianin tilianin tilianin
Sham 2VO tilianin +152I +193I +NCI
65kd

p65

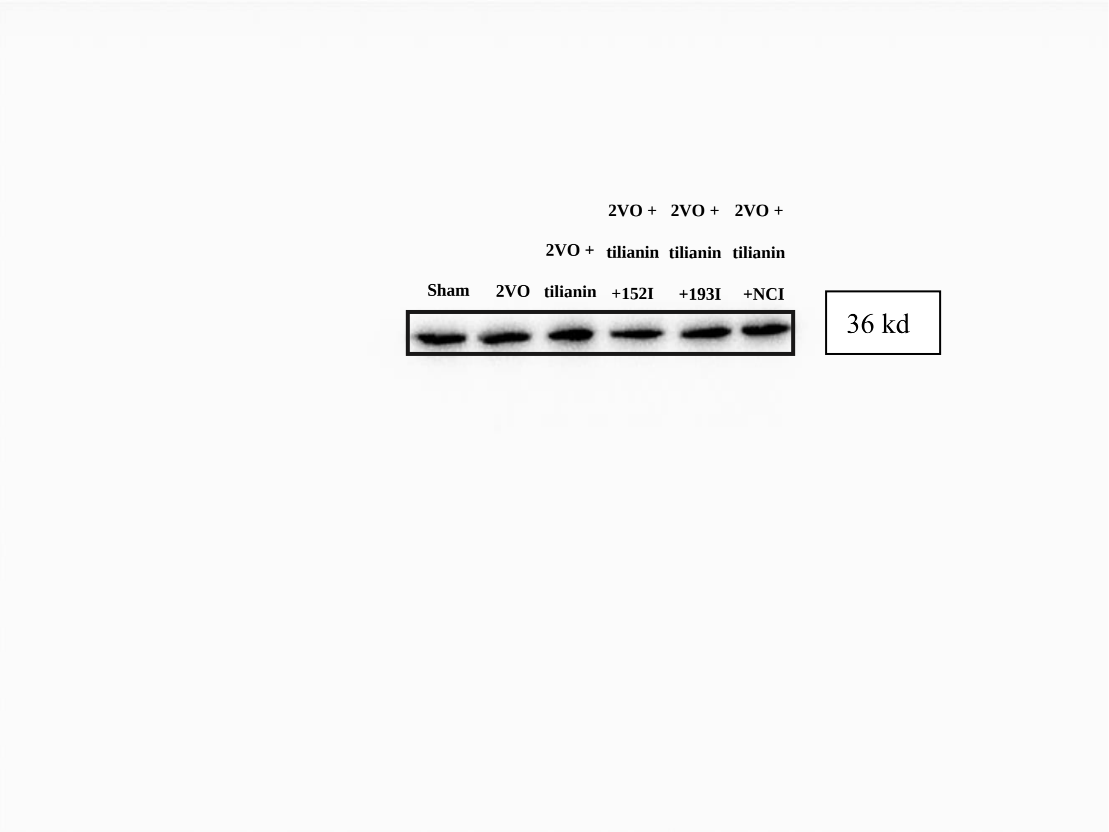
Sham
2VO
2VO + tilianin
2VO + tilianin +152I
2VO + tilianin +193I
2VO + tilianin +NCI
36 kd

GAPDH

Images in Figure 15 B

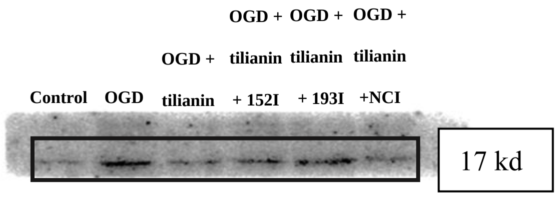

CaM

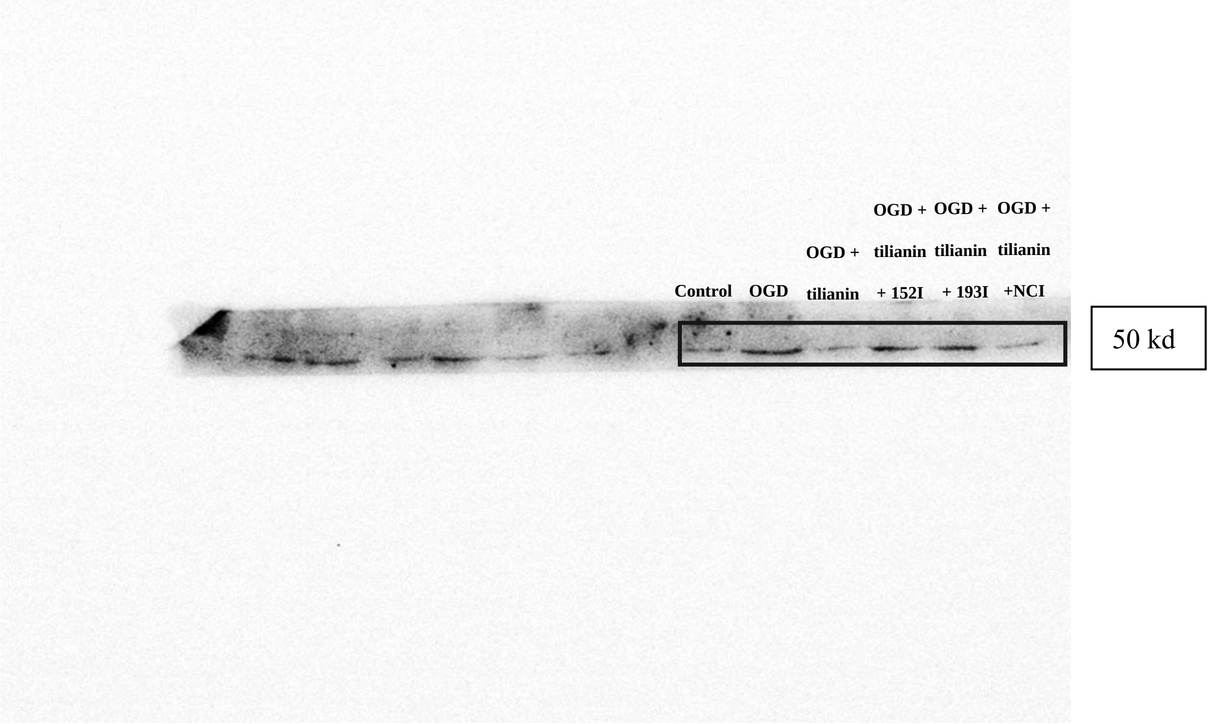

ox-CaMKIIα

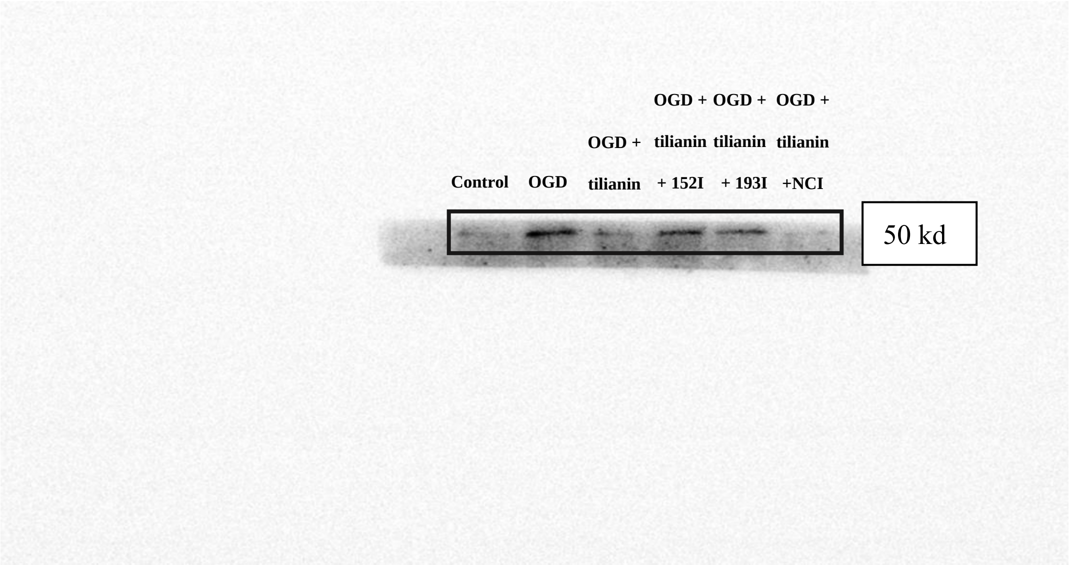

p-CaMKII

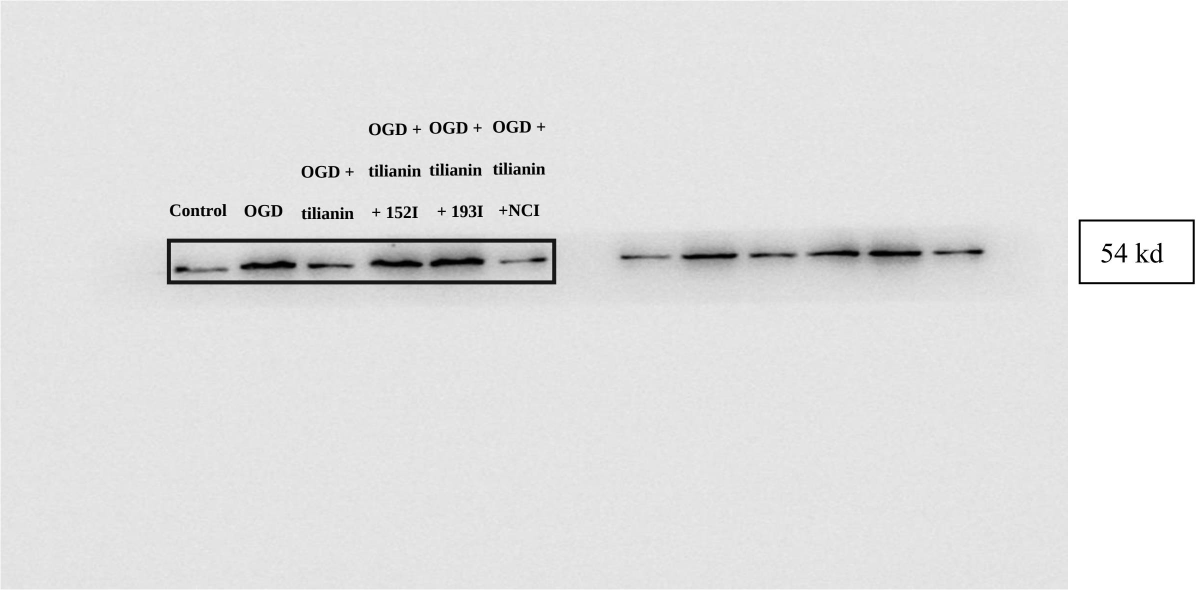

CaMKIIα

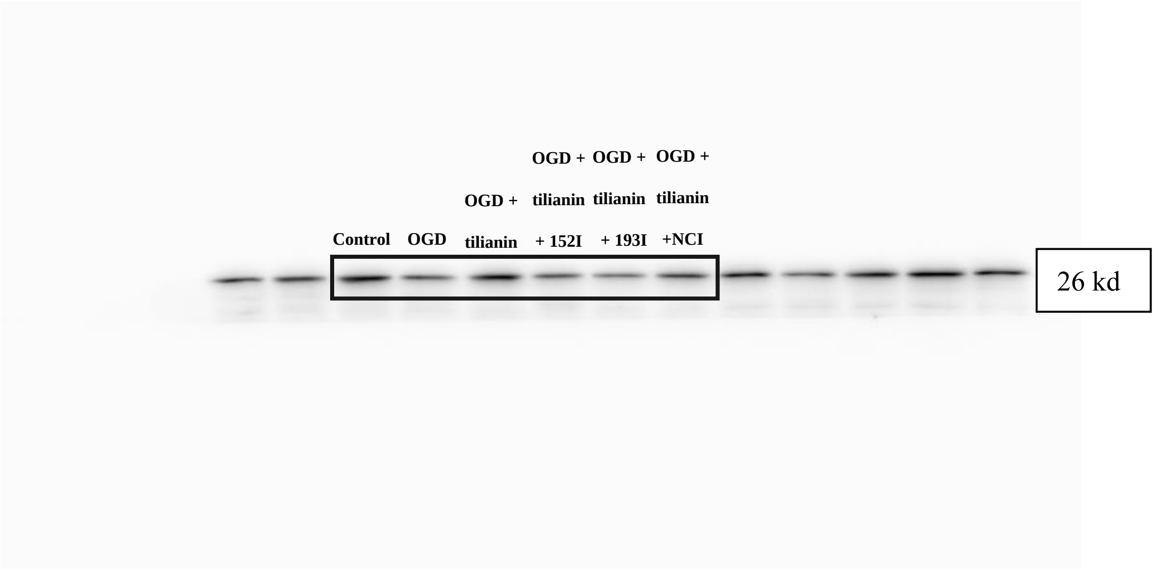
Control
OGD
OGD + tilianin
OGD + tilianin + 152I
OGD + tilianin + 193I
OGD + tilianin +NCI
26 kd

Bcl-2

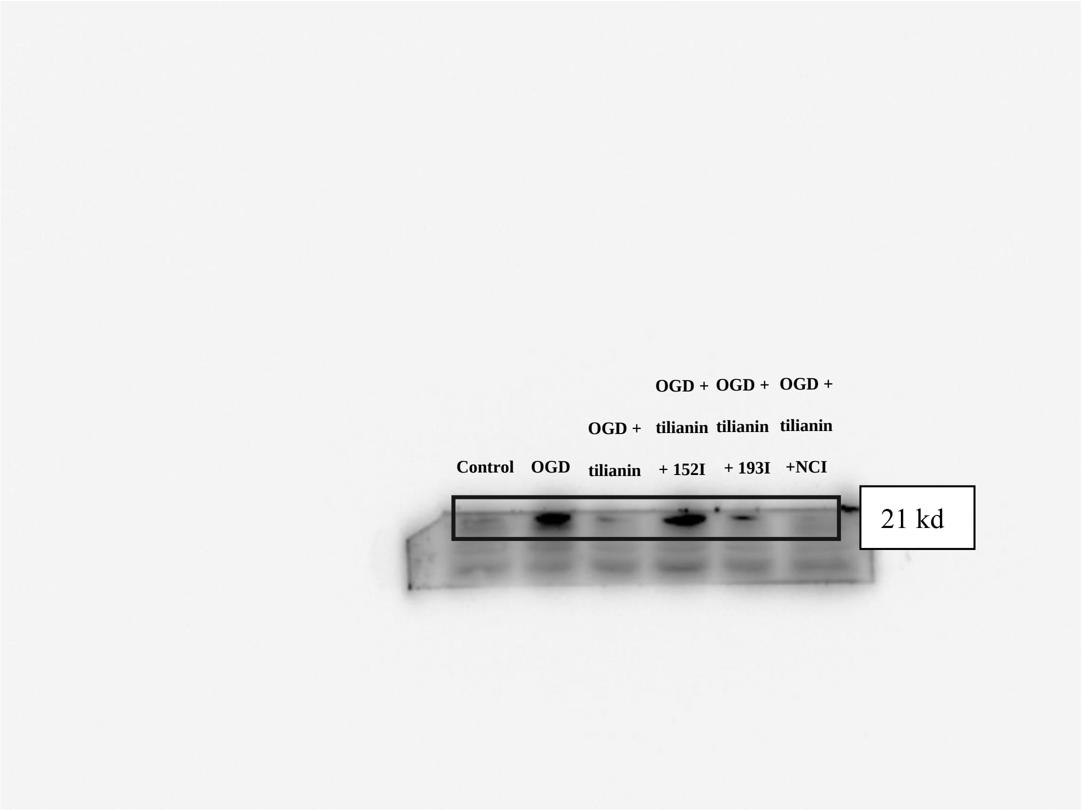
Control
OGD
OGD + tilianin
OGD + tilianin + 152I
OGD + tilianin + 193I
OGD + tilianin +NCI
21 kd

Bax

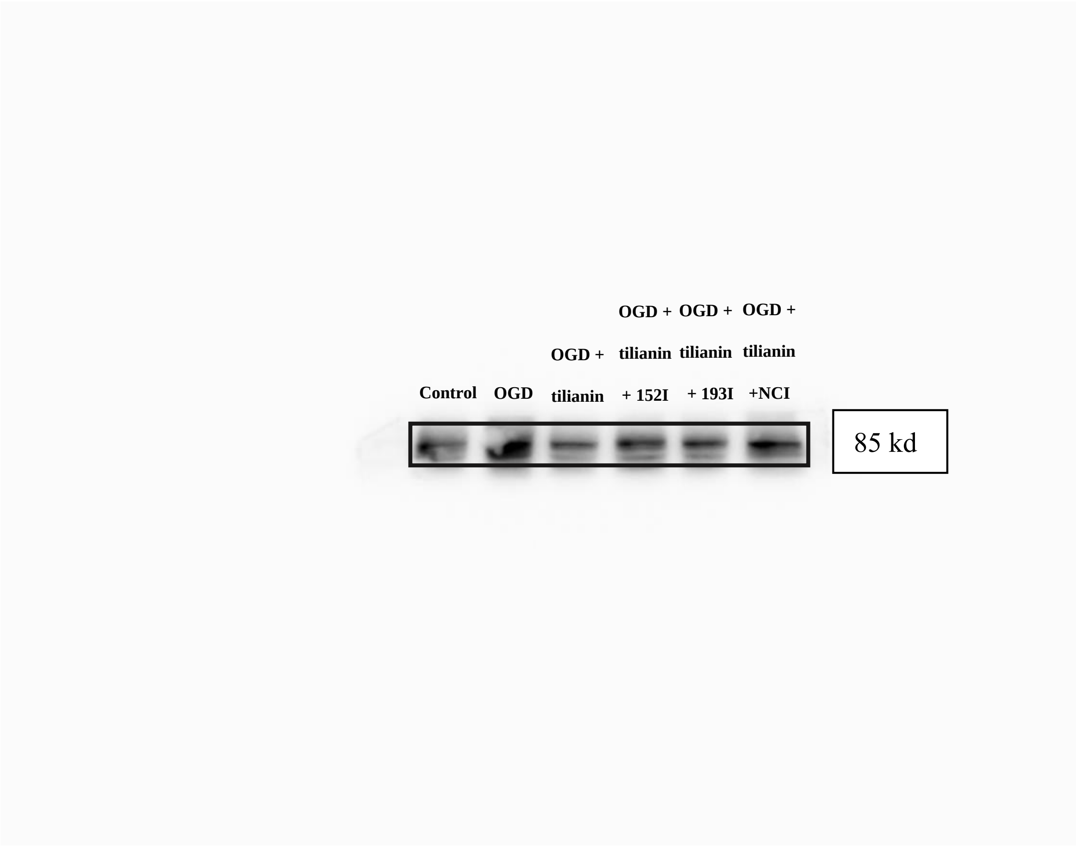
OGD +
OGD +
OGD +
OGD +
tilianin
tilianin
tilianin
Control
OGD
tilianin
+ 152I
+ 193I
+NCI
85 kd

c-PARP

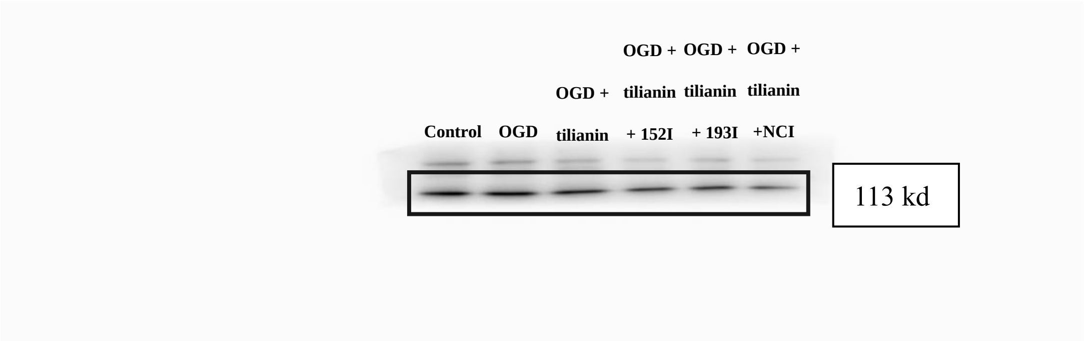
OGD +
OGD +
OGD +
OGD +
tilianin
tilianin
tilianin
Control
OGD
tilianin
+ 152I
+ 193I
+NCI
113 kd

PARP

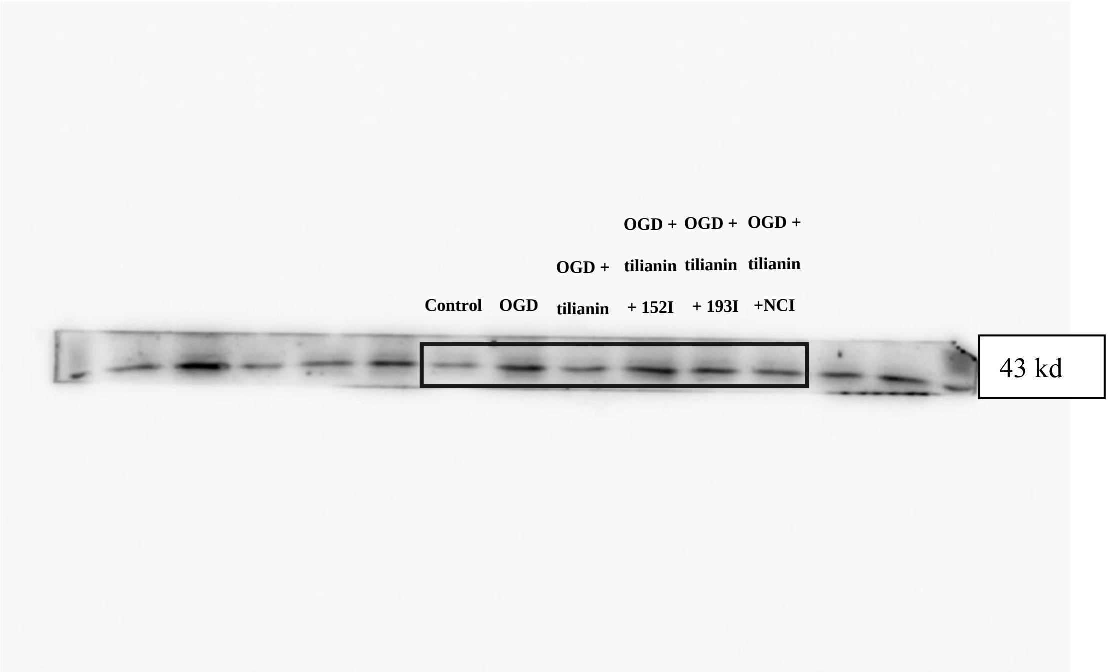
Control
OGD
OGD + tilianin
OGD + tilianin + 152I
OGD + tilianin + 193I
OGD + tilianin +NCI
43 kd

p-p38

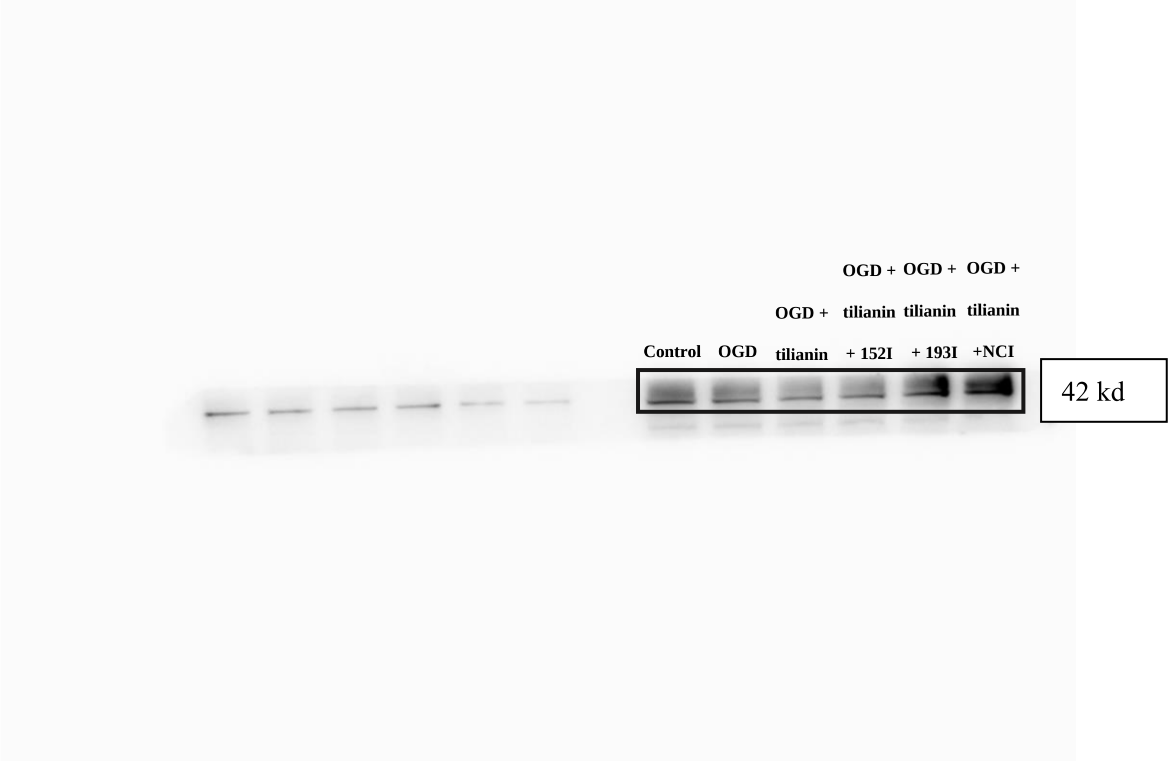
Control
OGD
OGD + tilianin
OGD + tilianin + 152I
OGD + tilianin + 193I
OGD + tilianin +NCI
42 kd

p38

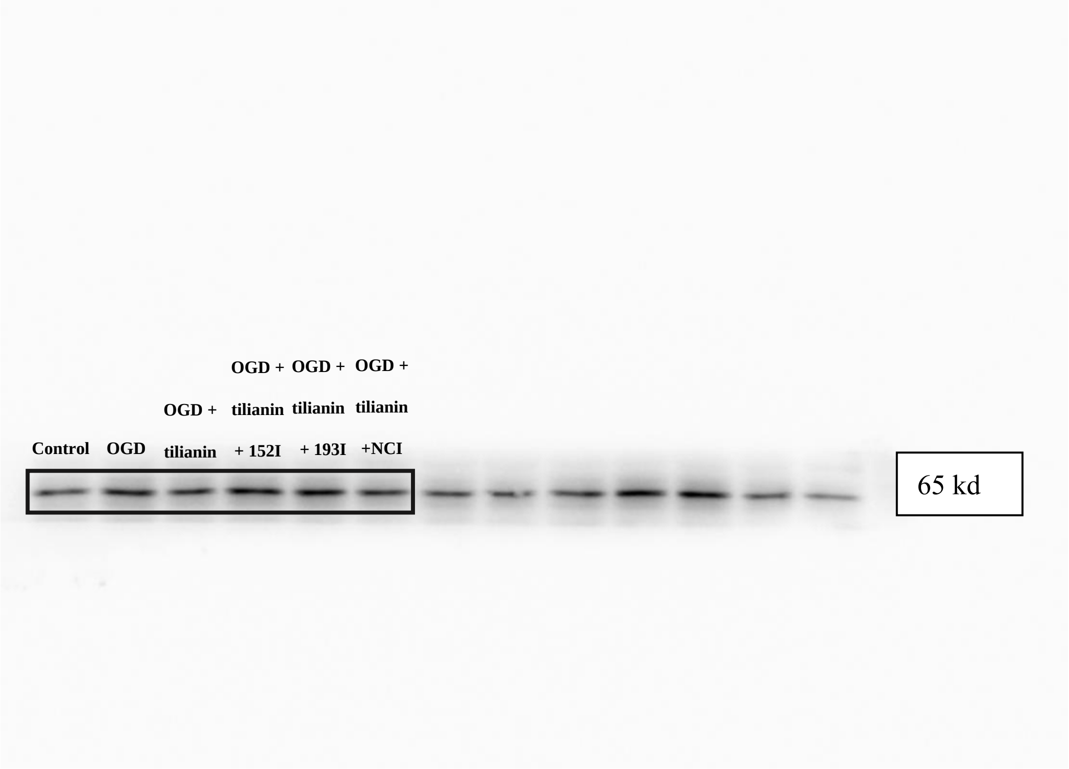
Control
OGD
OGD + tilianin
OGD + tilianin + 152I
OGD + tilianin + 193I
OGD + tilianin +NCI
65 kd

p-p65

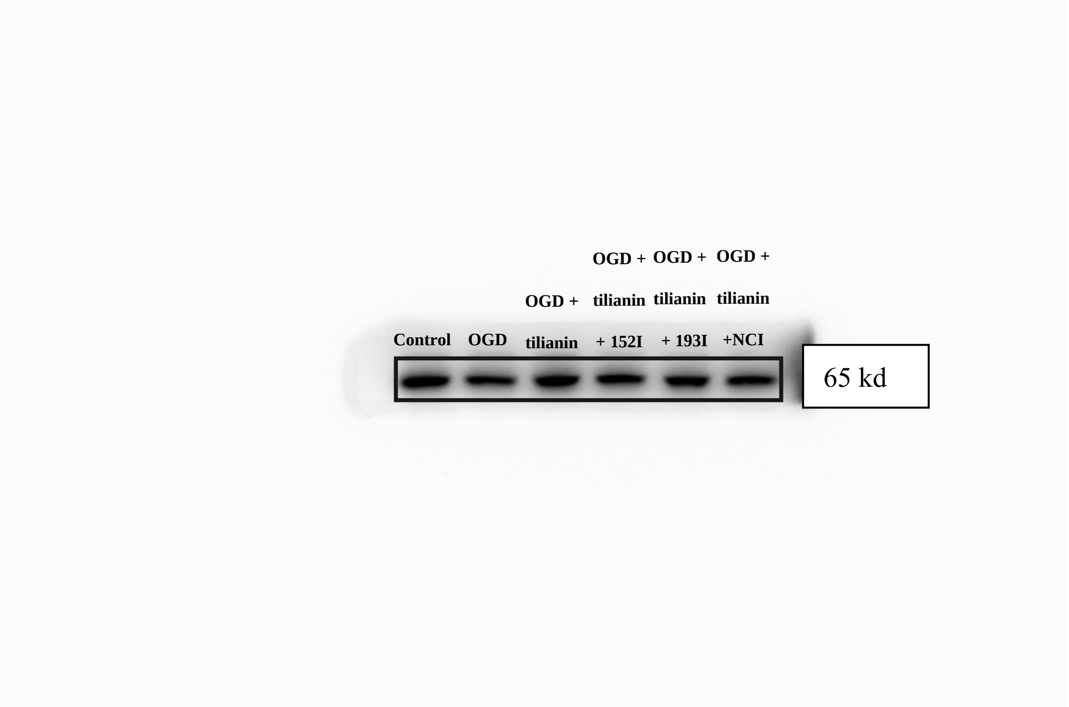
Control
OGD
OGD + tilianin
OGD + tilianin + 152I
OGD + tilianin + 193I
OGD + tilianin +NCI
65 kd

p65

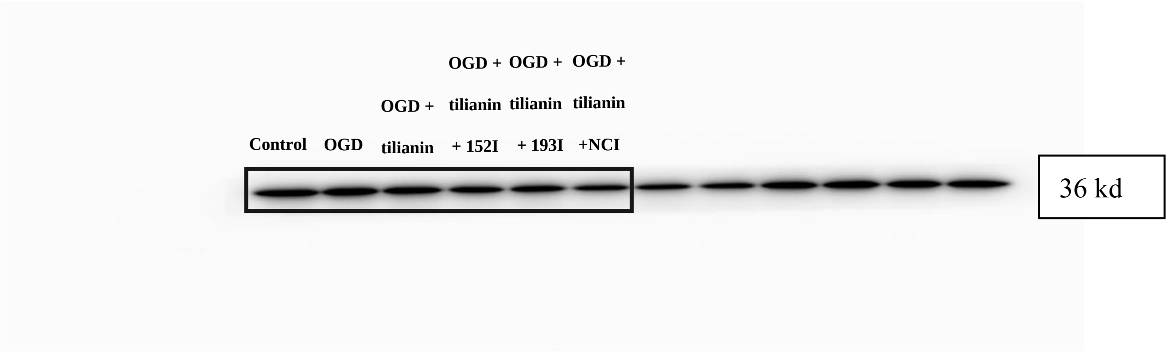
Control
OGD
OGD + tilianin
OGD + tilianin + 152I
OGD + tilianin + 193I
OGD + tilianin +NCI
36 kd

GAPDH

Images in Figure 15 I

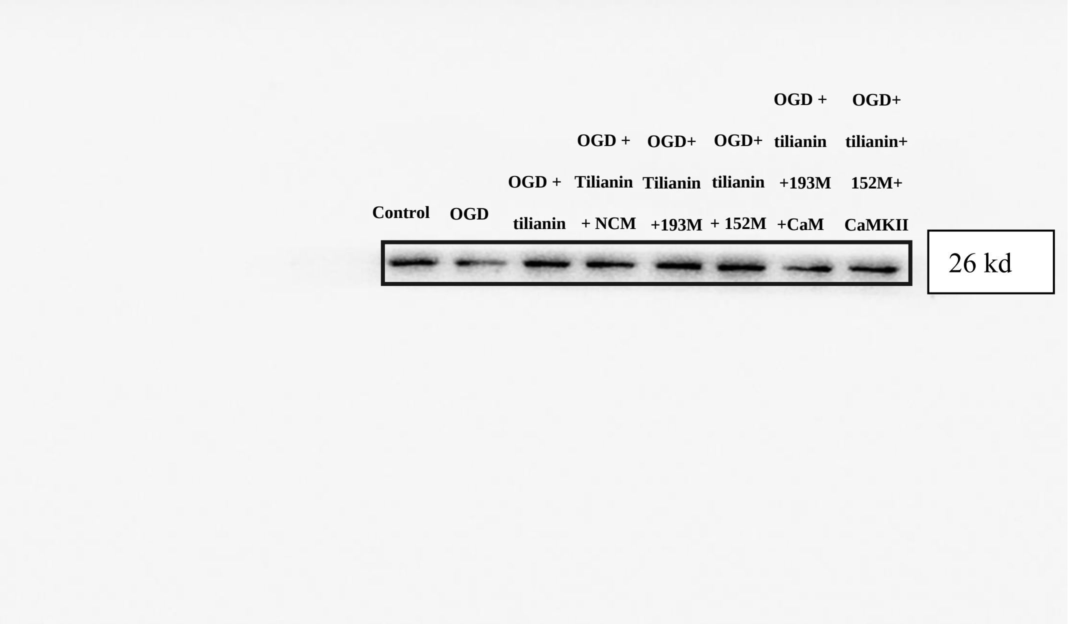

Bcl-2

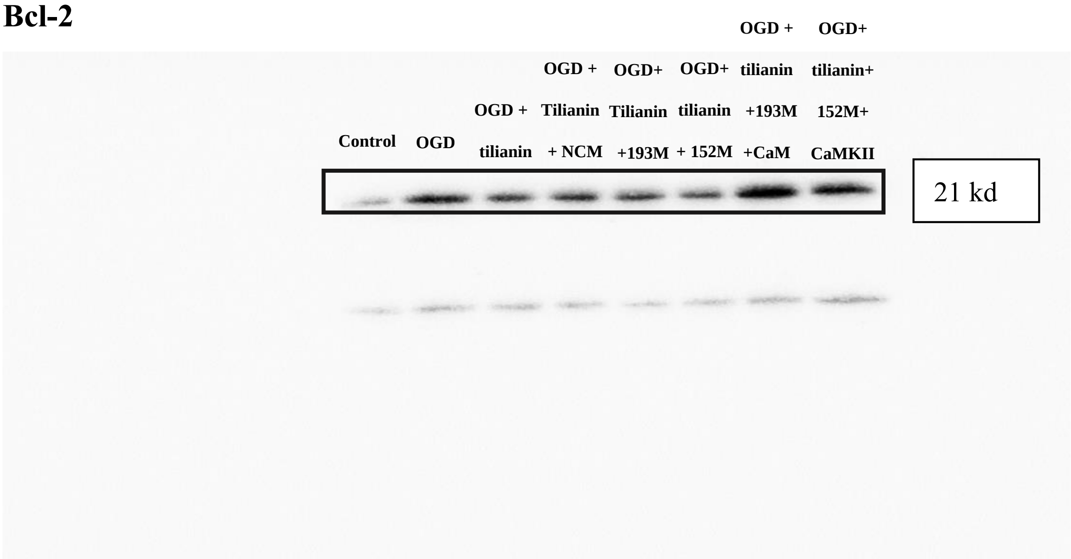

Bax

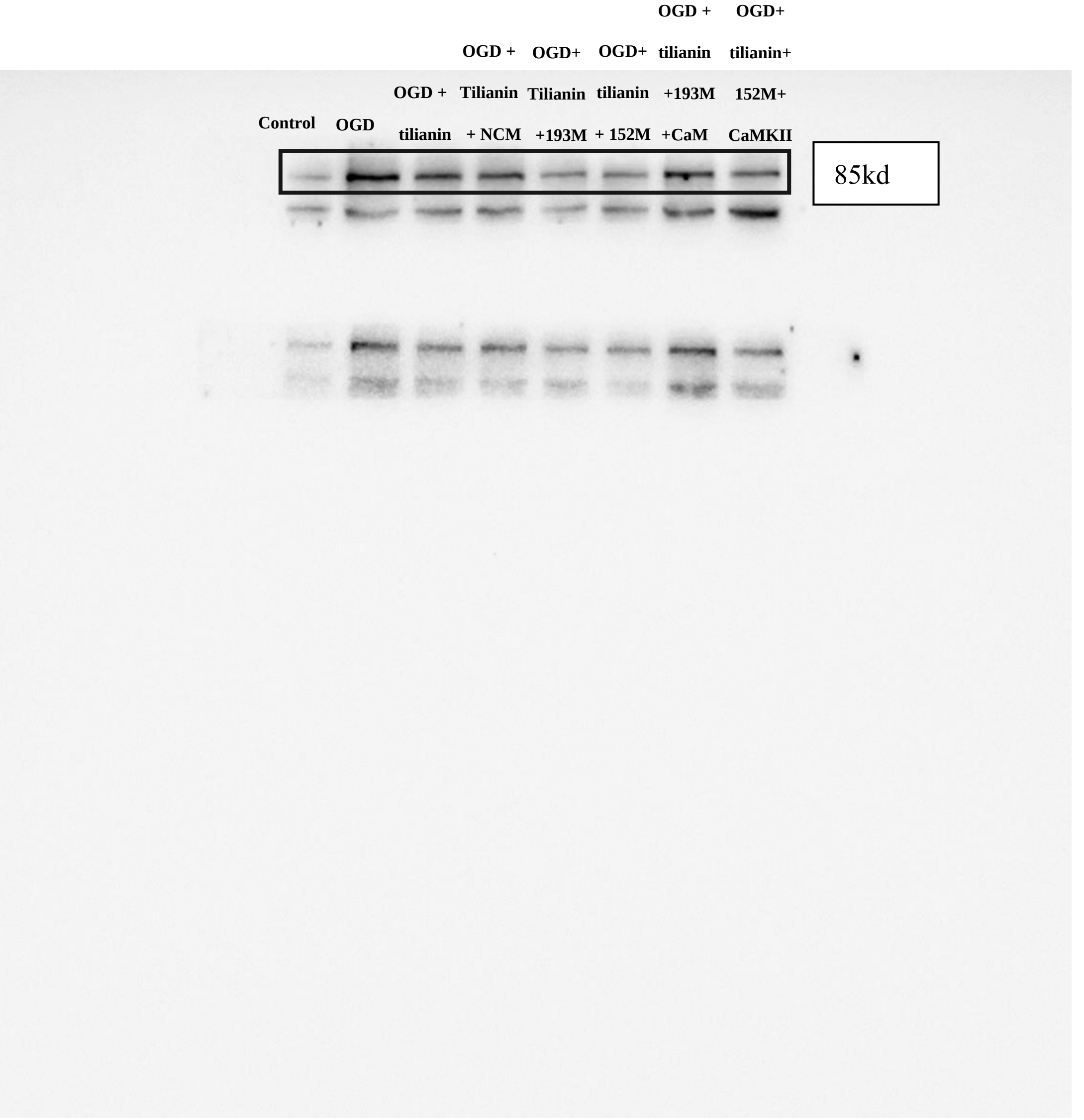
Control
OGD
OGD + tilianin
OGD + Tilianin + NCM
OGD+ Tilianin +193M
OGD+ tilianin + 152M
OGD + tilianin +193M +CaM
OGD+ tilianin+ 152M+ CaMKII
85kd

c-PARP

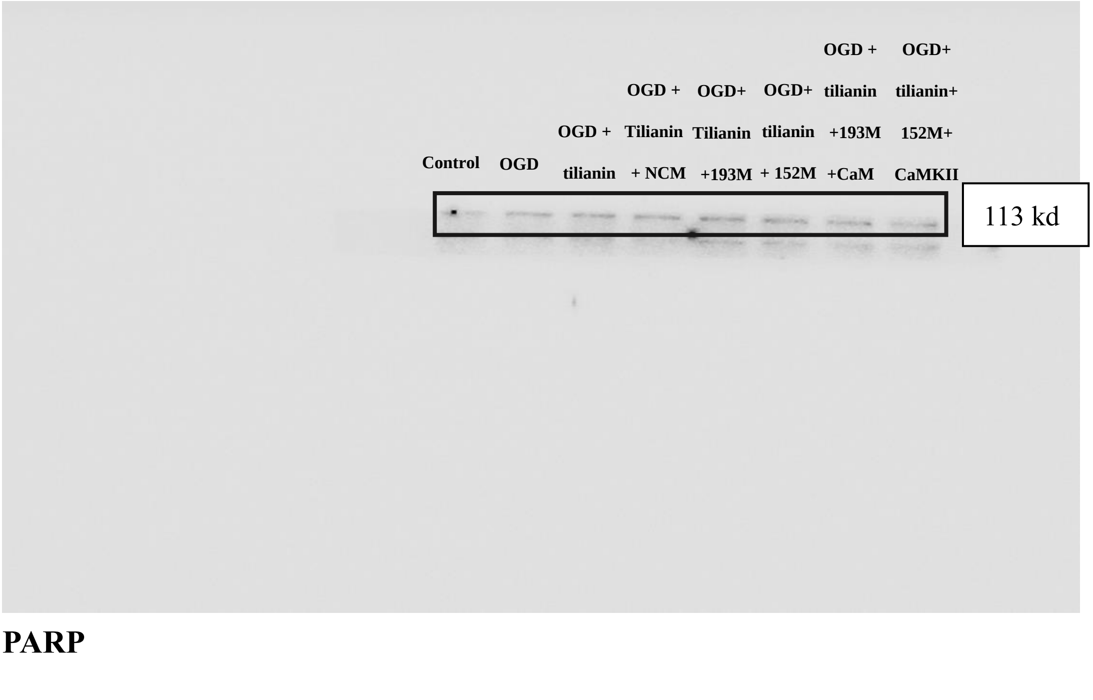
Control
OGD
OGD + tilianin
OGD + Tilianin + NCM
OGD+ Tilianin +193M
OGD+ tilianin + 152M
OGD + tilianin +193M +CaM
OGD+ tilianin+ 152M+ CaMKII
113 kd

PARP

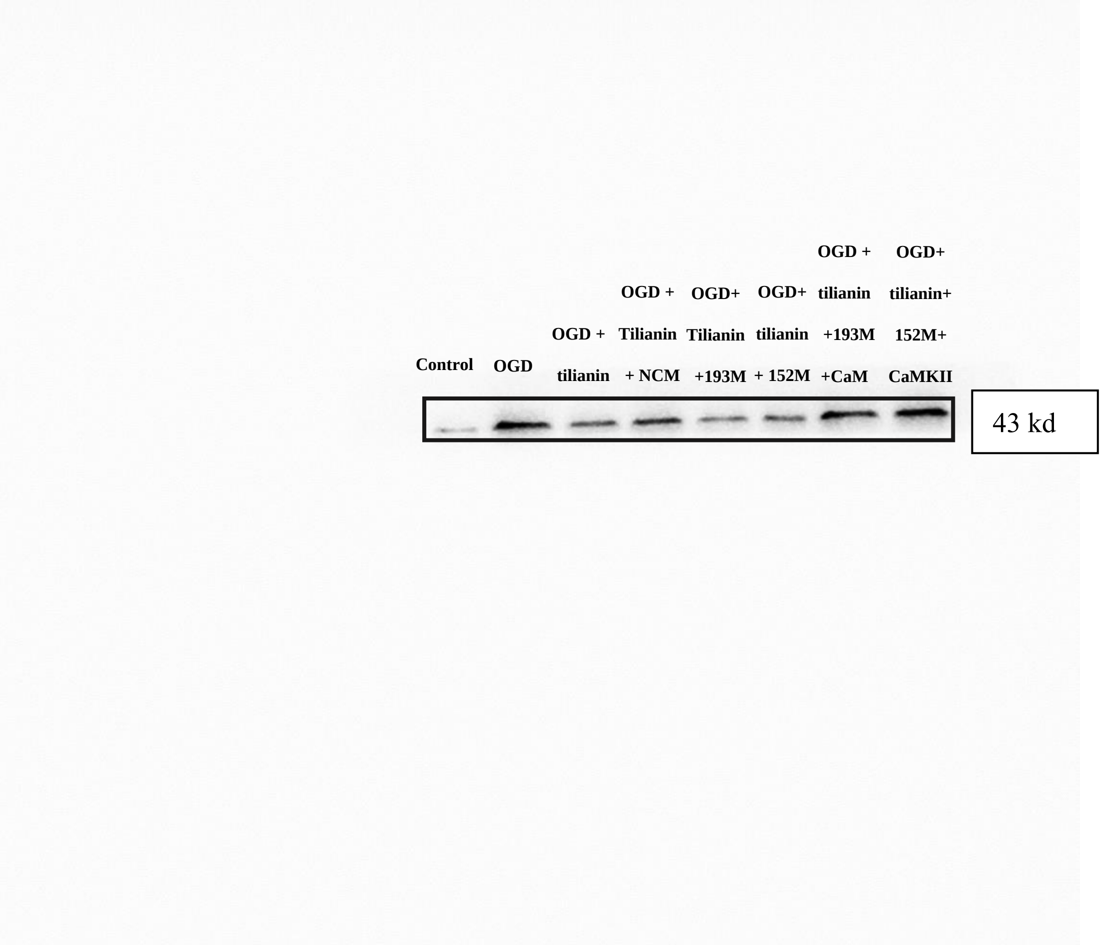
Control
OGD
OGD + tilianin
OGD + Tilianin + NCM
OGD+ Tilianin +193M
OGD+ tilianin + 152M
OGD + tilianin +193M +CaM
OGD+ tilianin+ 152M+ CaMKII
43 kd

p-p38

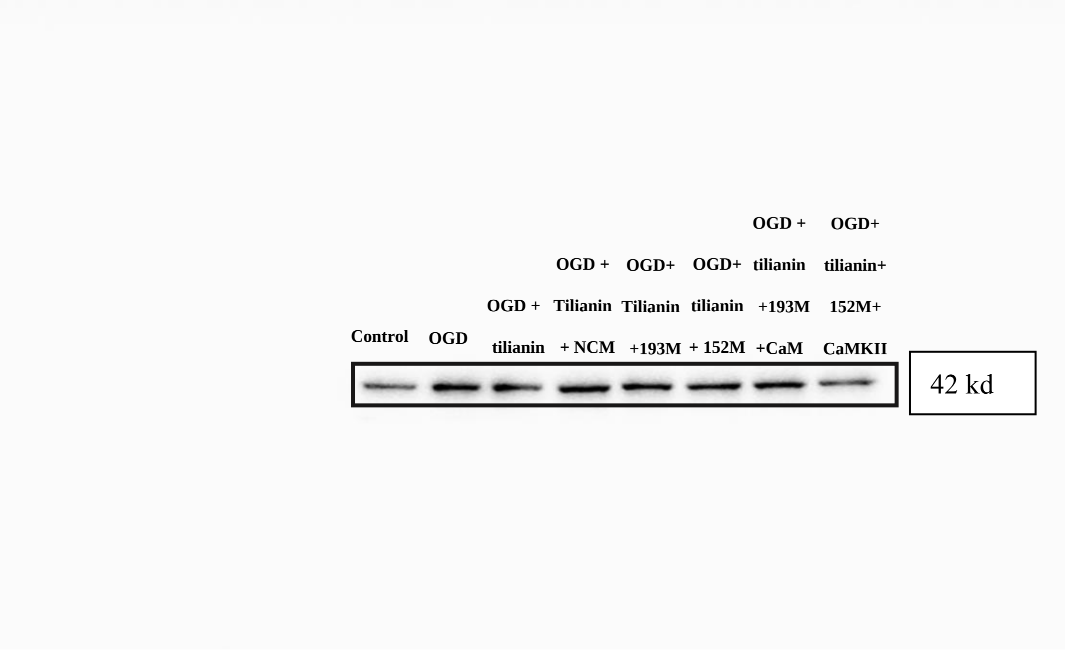
Control
OGD
OGD + tilianin
OGD + Tilianin + NCM
OGD+ Tilianin +193M
OGD+ tilianin + 152M
OGD + tilianin +193M +CaM
OGD+ tilianin+ 152M+ CaMKII
42 kd

p38

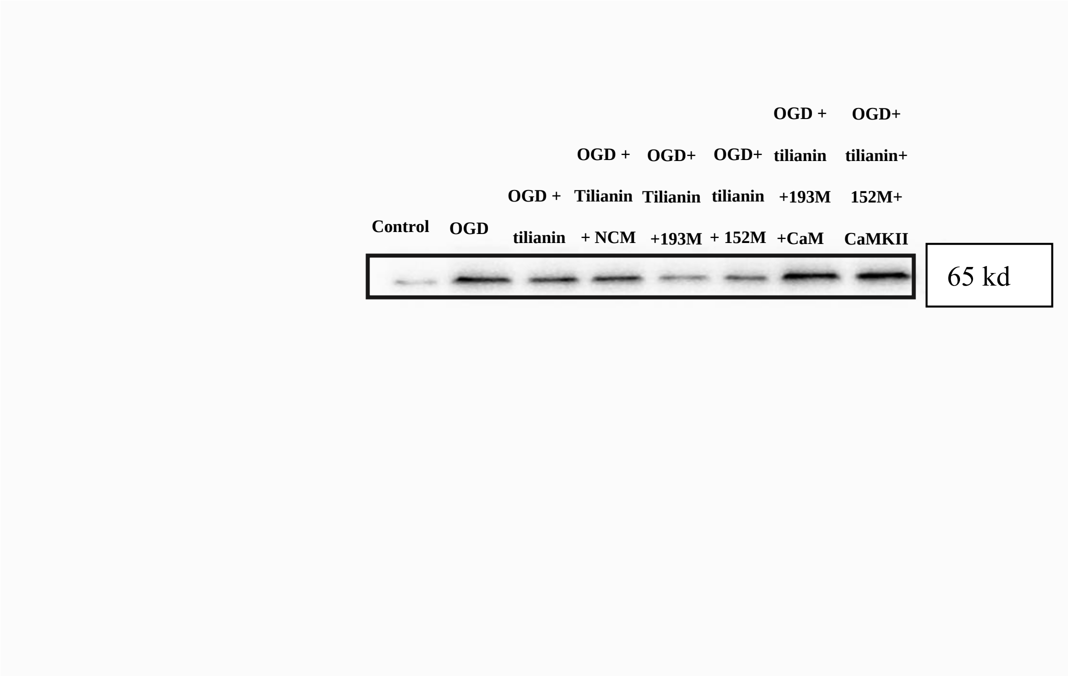
Control
OGD
OGD + tilianin
OGD + Tilianin + NCM
OGD+ Tilianin +193M
OGD+ tilianin + 152M
OGD + tilianin +193M +CaM
OGD+ tilianin+ 152M+ CaMKII
65 kd

p-p65

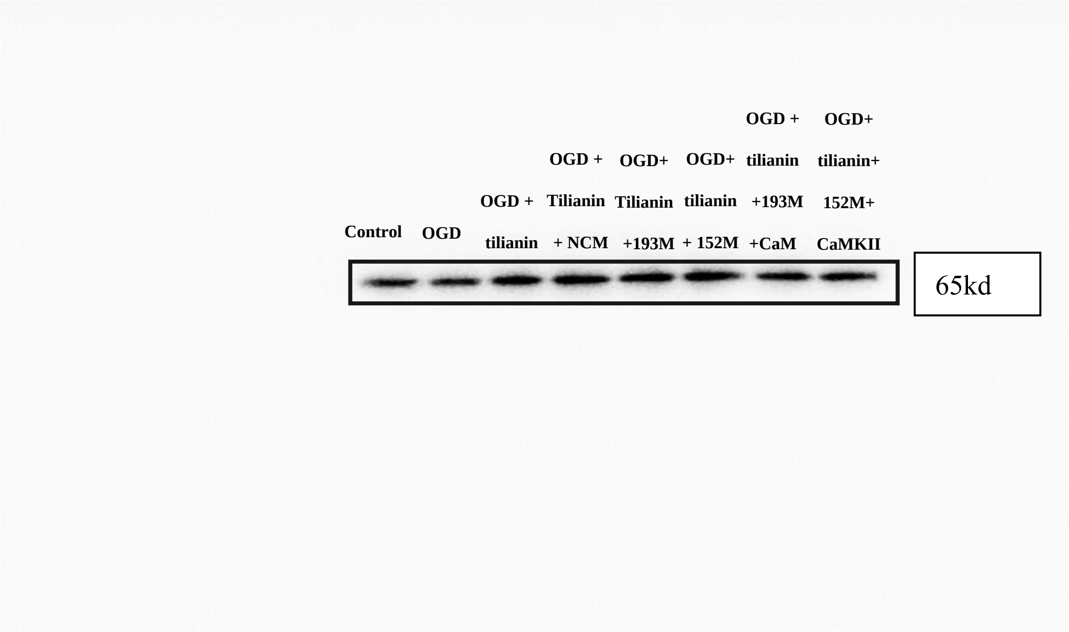
Control
OGD
OGD + tilianin
OGD + Tilianin + NCM
OGD+ Tilianin +193M
OGD+ tilianin + 152M
OGD + tilianin +193M +CaM
OGD+ tilianin+ 152M+ CaMKII
65kd

p65

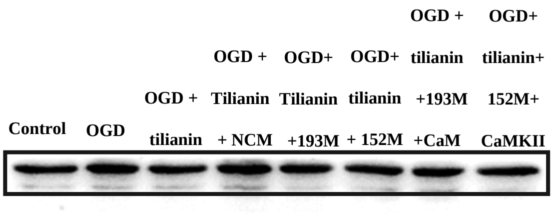
Control
OGD
OGD + tilianin
OGD + Tilianin + NCM
OGD+ Tilianin +193M
OGD+ tilianin + 152M
OGD + tilianin +193M +CaM
OGD+ tilianin+ 152M+ CaMKII

36 kd

GAPDH
